# Integrating cotyledon-based virus-induced gene silencing with visual marker promises a rapid, highly effective validation of gene functions in *Nepeta cataria*

**DOI:** 10.3389/fpls.2024.1514614

**Published:** 2025-01-21

**Authors:** Zongxia Yu, Ruo Lv, Bo Hong, Lei Yang

**Affiliations:** ^1^ Jiangxi Key Laboratory for Sustainable Utilization of Chinese Materia Medica Resources, Lushan Botanical Garden, Chinese Academy of Sciences, Jiujiang, Jiangxi, China; ^2^ Shanghai Key Laboratory of Plant Functional Genomics and Resources, Shanghai Chenshan Botanical Garden, Shanghai, China

**Keywords:** VIGS, cotyledon infiltration, visual marker, G8H, nepetalactone, catmint

## Abstract

*Nepeta* spp. generate volatile nepetalactone iridoids that have cat-attractant and insect-repellent activities. They differ from typical mint family (Lamiaceae) iridoids, which are non-volatile glucosides, and also vary from other species in the Nepetoideae sub-family, which do not generate iridoids. The chemistry and evolution of *Nepeta* make it suitable for further investigation. However, the lack of transgenic technology hampers the molecular and genetic investigations in *Nepeta*. Virus-induced gene silencing (VIGS) is a powerful tool to detect gene functions *in vivo*. Here, we constructed a modified VIGS method in *Nepeta cataria*, using cotyledon infiltration, with the gene silencing effect spreading to the first two pairs of true leaves. The VIGS efficiency reached as high as 84.4%, and the procedure takes only 3 weeks. We employed this method to validate the role of geraniol 8-hydroxylase in nepetalactone biosynthesis with *ChlH* as a visual marker in *N. cataria*. The method is also applicable to *Nepeta mussinii*. Thus, we developed an easy and effective VIGS approach, which will be advantageous for endogenous gene studies in two *Nepeta* species and holds the potential for application in other plants.

## Introduction

Virus-induced gene silencing (VIGS) is an easy and quick tool for genetic and functional validations of genes in non-model plants. VIGS makes use of the plant’s natural antiviral immune system to knock down endogenous gene levels by posttranscriptional gene silencing (PTGS) or transcriptional gene silencing (TGS). When plant viruses invade plants, they replicate and transcribe viral RNAs, which are processed into short interfering RNA (siRNA) by plant DICER-LIKE (DCL) and HUA ENHANCER 1 (HEN1) proteins. Then, siRNAs are equipped with ARGONAUTE (AGO) nucleases and other particles of RNA-induced silencing complex (RISC) to degrade the complementary gene transcripts or methylate the target gene (Matzke and Mosher, 2014; Carbonell and Carrington, 2015; Taochy et al., 2017). Beyond gene silencing, the derived toolbox of VIGS has broadly expanded to encompass virus-induced overexpression (VOX), virus-induced genome editing (VIGE), and host-induced gene silencing (HIGS) (Cheuk and Houde, 2019; Jiang et al., 2021; Lei et al., 2021), expanding the applications from clarifying gene functions to revealing molecular mechanisms, protecting crops from biological and abiotic stresses (Wu et al., 2020; Yao et al., 2020).

The viruses used for VIGS are mostly single-stranded DNA viruses like the African cassava mosaic virus (ACMV) or RNA viruses like the foxtail mosaic virus for monocots and tobacco rattle virus (TRV) for dicots (Wu et al., 2011; Liu et al., 2016; Rössner et al., 2022). TRV is commonly used, as it has a wide host range and the ability to infect meristematic tissue (Li et al., 2018). The genome of TRV has been disassembled and constructed into two vectors, named TRV1 and TRV2. TRV1 comprises replicases, a transmission protein, and a cysteine-rich protein, while TRV2 contains a coat protein and a multiple cloning site for the insertion of the target gene’s fragment (Shi et al., 2021). The fragment is selected from the coding sequence of the target gene with a length of 200–400 bp. The viral genome harboring the target gene’s fragment is then delivered to the plants mediated by *Agrobacterium tumefaciens* in diverse ways such as syringe injection, vacuum infiltration, spraying, and rubbing.

*Nepeta* spp. generate iridoids, a type of monoterpenoid. These are widespread in the Lamiaceae family but exclusive to the *Nepeta* genus within the Nepetoideae sub-family. This characteristic renders *Nepeta* a model genus for investigating iridoid evolution within the Lamiaceae family (Boachon et al., 2018). Nepetalactone represents the major iridoid of *Nepeta* spp. and is well-known for attracting cats; this is why *Nepeta* spp. are also called catmint or catnip (Uenoyama et al., 2021). The biosynthesis of nepetalactone starts with geranyl pyrophosphate (GPP), which is converted into geraniol, 8-hydroxygeraniol, and 8-oxogeranial under the catalysis of geraniol synthase (GES), geraniol 8-hydroxylase (G8H), and 8-hydroxygeraniol oxidoreductase (HGO), respectively. Then, iridoid synthase (ISY) reduces 8-oxogeranial to form nepetalactol, the precursor to iridoids in plants. Nepetalactol-related short-chain dehydrogenases (NEPSs) and a major latex protein-like (MLPL) enzyme can contribute to the stereoselectivity of cyclization during the conversion of 8-oxogeranial to nepetalactol (Lichman et al., 2019; Lichman et al., 2020). Then, NEPSs convert nepetalactol isomers to nepetalactone (Lichman et al., 2020; Smit and Lichman, 2022). However, the lack of a mutant pool and transgenic tools in *Nepeta* spp. impedes the verification of the gene function *in planta*.

VIGS is an alternative for elucidating gene function in non-model plants recalcitrant to stable transformation including the Solanaceae, Cruciferae, Malvaceae, and Gramineae families (Shi et al., 2021). Within Lamiaceae, it has been successfully utilized to resolve pentacyclic triterpene biosynthesis in *Ocimum basilicum* (Misra et al., 2017). Palmer et al. successfully applied VIGS in *Nepeta cataria* by inoculating the nodes in *N. cataria* cuttings together with visual markers and thereby validated the functions of *GES*, *ISY*, and *MLPL* (Palmer et al., 2022). Herein, we introduce a more rapid and effective VIGS method applicable to *N. cataria* and *Nepeta mussinii* using cotyledon infiltration alongside a visual marker. Through this approach, we provide *in vivo* validation of *G8H* participating in nepetalactone biosynthesis.

## Methods

### Plant growth conditions

The seeds of *N. cataria* and *N. mussinii* were bought from SeedCorner Kings Seeds (https://www.kingsseedsdirect.com/). The seeds were sowed in 1-cm depth under the compost by toothpicks and cultured under 16/8-h light and 25°C/22°C day/night regime. For material collection, the chlorotic area of the infected leaves from three plants was cut and collected as one sample and immediately frozen in liquid nitrogen (LN2); at least three biological samples were prepared for each vector. The sample was ground into powder by TissueLyser II (QIAGEN, Valencia, CA, USA), separated, and weighed for qRT-PCR and gas chromatography–mass spectrometry (GC–MS) analysis.

### Vector construction

**Figure 1:**
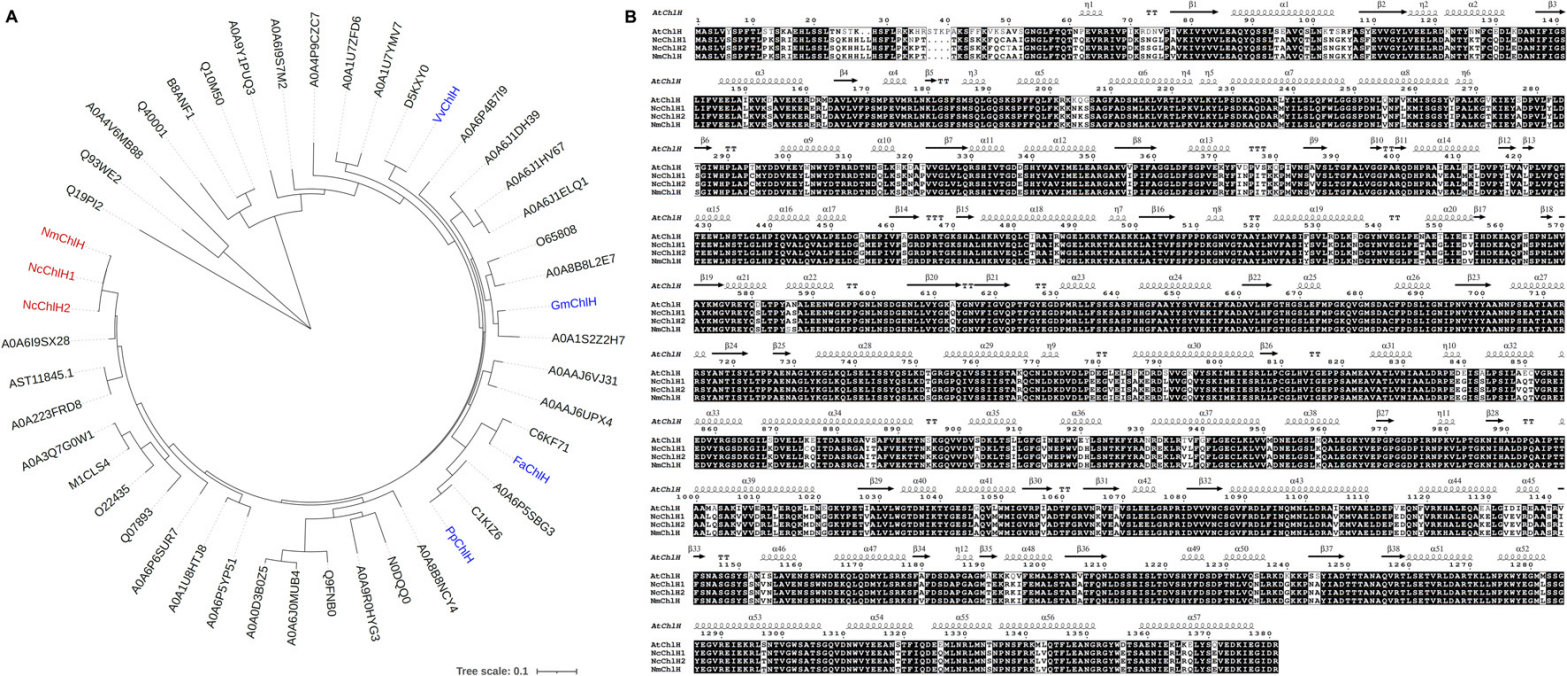
Phylogenetic analysis of *Nepeta* ChlHs. **(A)** Phylogenetic tree of *Nepeta* ChlHs with their homologs. The homologs (**Supplementary Data Sheet S3**) were identified from the UniProt database (https://www.uniprot.org/) using VvChlH, GmChlH, PpChlH, and FaChlH protein sequences as the Basic Local Alignment Search Tool-protein (BLASTP) queries, which were marked in blue color. The protein sequences of NcChlH1, NcChlH2, and NmChlH1 were directly obtained from *Nepeta cataria* and *Nepeta mussinii* transcriptome databases and were marked in red color. The tree was constructed using the maximum likelihood algorithm in TBtools (Chen et al., 2023). Branch point bootstrap values were calculated with 1,000 replicates. The tree was drawn to scale, with branch lengths measured in the number of substitutions per site. **(B)** Alignment of the protein sequences of *Nepeta* ChlHs with AtChlH. *Nepeta* ChlH sequences were aligned with the amino acid sequence and secondary structure of AtChlH (Q9FNB0) on the ESPript 3.0 website (https://espript.ibcp.fr/ESPript/ESPript/).

*ChlH*s from *N. cataria* and *N. mussinii* were picked by alignment with reported *ChlH*s from other plants (**Figure 1**). The amino acid sequences of *Vitis vinifera* (VvChlH, NP_001268078.1), *Glycine max* (GmChlH, AXB26707.1), *Prunus persica* (PpChlH, ACO57443.1), and *Fragaria* × *ananassa* (FaChlH, AEN74910.1) were used as queries to search the homologous genes from the database of catmint (https://datadryad.org/stash/dataset/doi:10.5061/dryad.88tj450). The *ChlH* (329 bp) fragment was designed from the conserved region of *NcChlH* and *NmChlH* in order to work across both species with one vector, as the identities of *ChlH*s were high. *GES* (366 bp) and *G8H* (288 bp) were designed from the specific region of the coding sequences (CDSs) of the genes (**Supplementary Data Sheet S1**). All fragments were searched throughout the genomes of *N. cataria* and *N. mussinii* to avoid off-target effects and then amplified with overhangs homologous to the TRV2 vector using Phusion Plus DNA Polymerase (Thermo Fisher, Waltham, MA, USA; F630S). The *ChlH* fragment was inserted into TRV2 between *Eco*RI and *Bam*HI, and *GES* and *G8H* were cloned between *Xho*I and *Sma*I using homologous recombination (Vazyme, Nanjing, China; C112-00).

### RNA extraction, reverse transcription, and qRT-PCR analysis

Powdered tissue (~100 mg) was used for RNA extraction according to the instructions of the plant RNA purification kit (Vazyme, RC401-01). Total RNA (~1 μg) was used for reverse transcription to generate cDNA following the instructions of M-MLV Reverse Transcriptase (Invitrogen, Carlsbad, CA, USA; 28025-013). The primers for qRT-PCR analysis were designed behind the VIGS fragment. The cDNAs were diluted 10 times before performing the qRT-PCR using SYBR Green qPCR Master Mix (Selleckchem, Houston, TX, USA; B21202) on Bio-Rad CFX96. The method of 2^−ΔΔCt^ was used to measure gene expression level with ubiquitin as the internal standard. The primers used for vector construction and qRT-PCR analysis are listed in **Supplementary Data Sheet S2**.

### Cotyledon-based virus-induced gene silencing in *N. cataria* and *N. mussinii*

The TRV bipartite system was selected for VIGS, which includes two vectors. TRV1 ensures the replication and movement of viral functions, and TRV2 encodes the coat protein and the fragment of the target gene for VIGS. TRV1 and TRV2 were transformed into *A. tumefaciens* GV3101 using the freeze–thaw method separately. The positive clones were inoculated in 1 mL Luria-Bertani (LB) medium containing kanamycin and gentamycin (50 mg/L) antibiotics and cultured overnight at 28°C, shaking at 220 rpm; 200-μL overnight cultures were inoculated in 10 mL LB with antibiotics, 10 mM MES, and 20 μM acetosyringone and shaken overnight at 28°C and 220 rpm. The cultures were centrifuged at 3,500 ×*g* for 15 min and resuspended in 2 mL fresh prepared infiltration buffer (10 mM MES, pH 5.8, 10 mM MgCl_2_, and 200 μM acetosyringone). The OD_600_ was adjusted to 2.5, and TRV1 with TRV2 were mixed at the volume ratio of 1:1. The mixture was shaken for 3 h at 28°C and 100 rpm in the dark before being infiltrated into the cotyledons of seedlings 7 days after sowing using a 1-mL syringe. Fifteen to 40 seedlings were infiltrated for each group. The chlorotic phenotype appeared as early as 6 days after infiltration. The chlorotic parts from the first and second true leaves were collected 2 weeks post infiltration, or the whole first and second true leaves were collected when visual marker was lacking in the control, flash frozen in LN2, and stored in −80°C freezer for further qRT-PCR and GC–MS analysis.

### Metabolites extraction and GC–MS analysis

Tissue powder (~50 mg) was weighed and transferred into a 2.0-mL tube, and 600 μL MeOH was added, vortexed for 10 s, and shaken for 10 min; 600 μL of hexane containing 30 ng/μL nonyl acetate as internal standard was added, vortexed, and shaken for 20 min. The mix was centrifuged at room temperature and top speed (14,600 ×*g*) for 10 min; 400 μL of the upper hexane layer was transferred into a new 1.5-mL tube and centrifuged again, and the supernatant was collected for GC–MS analysis.

For GC–MS analysis, the samples were injected in split mode (2 μL, split ratio 5:1) at an inlet temperature of 220°C. The column of Zebron ZB-5HT-INFERNO (30 m × 250 μm × 0.1 μm) was used for separation. Helium was used as carrier gas with a flow rate of 1.2 mL/min. The program was as follows: hold at 80°C for 5 min, increase to 110°C at a rate of 2.5°C/min, enhance to 280°C at 50°C/min, and hold for 4 min. Nonyl acetate (30 ng/μL, Sigma-Aldrich Corp., St. Louis, MO, USA) was used as the internal standard, and the contents of metabolites were calculated by comparing them to the internal standard.

## Results

### Cotyledon-based VIGS with rapid process and high efficiency stands out from other infection approaches in *N. cataria*

Magnesium chelatase subunit H (ChlH) participating in chlorophyll biosynthesis was chosen as the visual marker, as it will cause the chlorotic phenotypes to appear when knocked down by VIGS (Palmer et al., 2022). Four reported ChlHs (VvChlH, GmChlH, PpChlH, and FaChlH) were employed as queries to identify the native ChlHs from the databases of both *N. cataria* and *N. mussinii*, which were designated as NcChlH1, NcChlH2, and NmChlH1. *Nepeta* ChlHs exhibit high amino acid sequence identities with their homologs and share the highest homology with *Sesamum indicum* (**Figure 1**), suggesting that ChlH genes are highly conserved throughout evolution. The transcription levels of these *Nepeta ChlH*s are the most abundant in immature leaves, followed by mature leaves and closed flower buds (**Supplementary Figure S1**), which is in accordance with their roles in chlorophyll biosynthesis.

**Figure 2:**
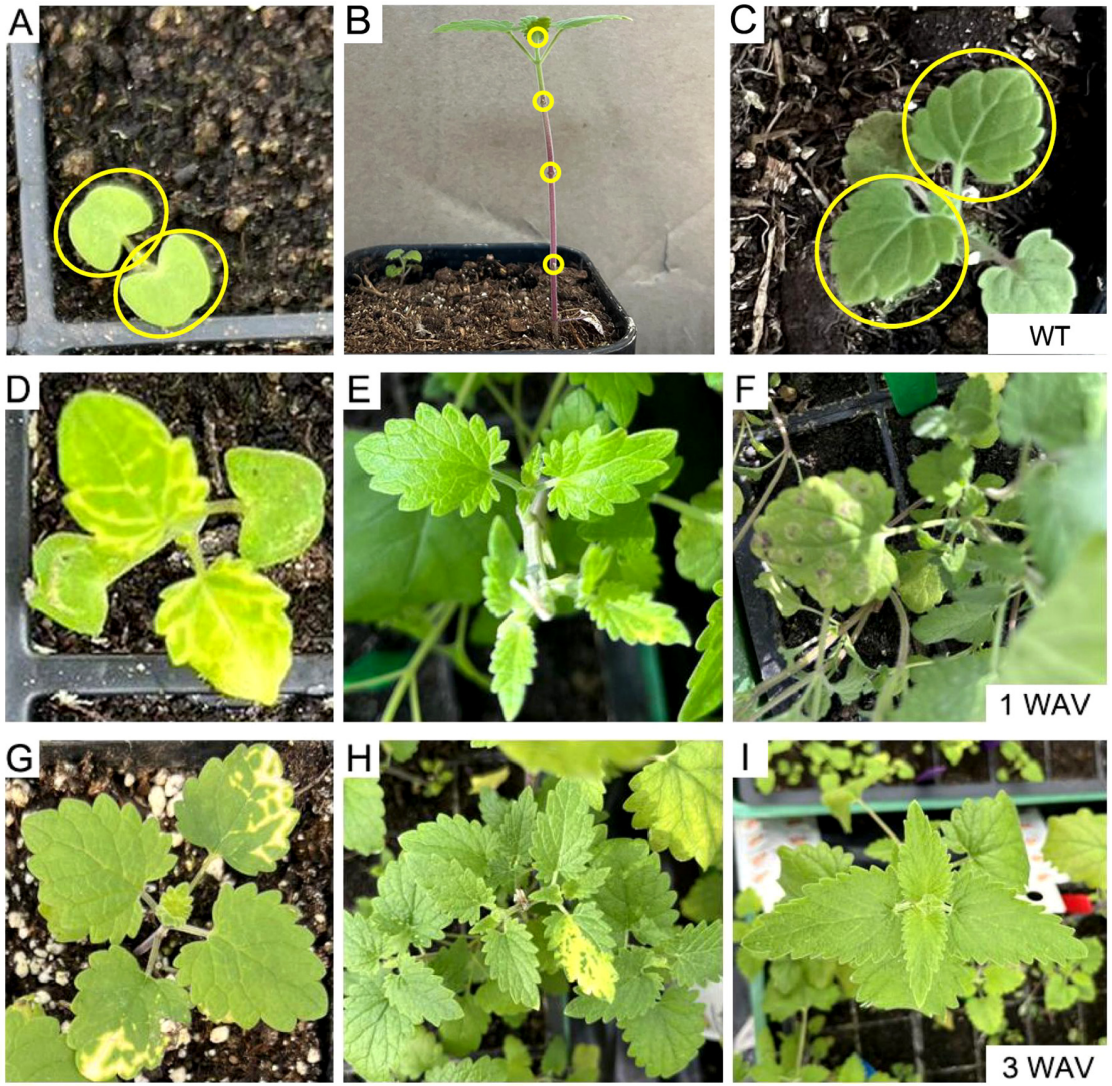
Comparison of different virus-induced gene silencing (VIGS) approaches in *Nepeta cataria*. **(A, D, G)** Cotyledon-based VIGS was used. **(B, E, H)** The wounded node method. **(C, F, I)** The true-leaf VIGS. **(A–C)** The wild type (WT) before treatment; the infiltration parts are circled in yellow. **(D–F)** One week after VIGS. **(G–I)** Three weeks later.

Three convenient VIGS infection methods (independent of vacuum devices) were compared in *N. cataria*: 1) cotyledon-based, which involved the cotyledons of the 7-day-old seedlings being infiltrated with the VIGS infection solution using a syringe; 2) wounded nodes, which was the approach described by Palmer et al (Palmer et al., 2022), wherein the leaves under the shoot of 1-month-old plants were removed from each node, both the nodes and the shoot apical meristem were punctured by toothpick and soaked with infection solution; 3) true-leaf VIGS, wherein the abaxial side of the leaves of 2-week-old plants was infiltrated with a syringe as described in many other plants. The chlorotic phenotype manifested in both cotyledon-based and wounded nodes approaches and could be observed as early as 1 week after VIGS (WAV) in the newly emerging leaves instead of the infiltrated ones (**Figure 2**). However, true-leaf VIGS did not work in the trials in *N. cataria*, considering that the second approach needed approximately 1 month for plant growth to provide sufficient nodes (≥3 nodes) and performed lower VIGS efficiency in our trials. Consequently, the quicker cotyledon-based VIGS was selected for further investigation.

### The chlorotic phenomenon spread only to the first and second pairs of true leaves in both *N. cataria* and *N. mussinii*

**Figure 3:**
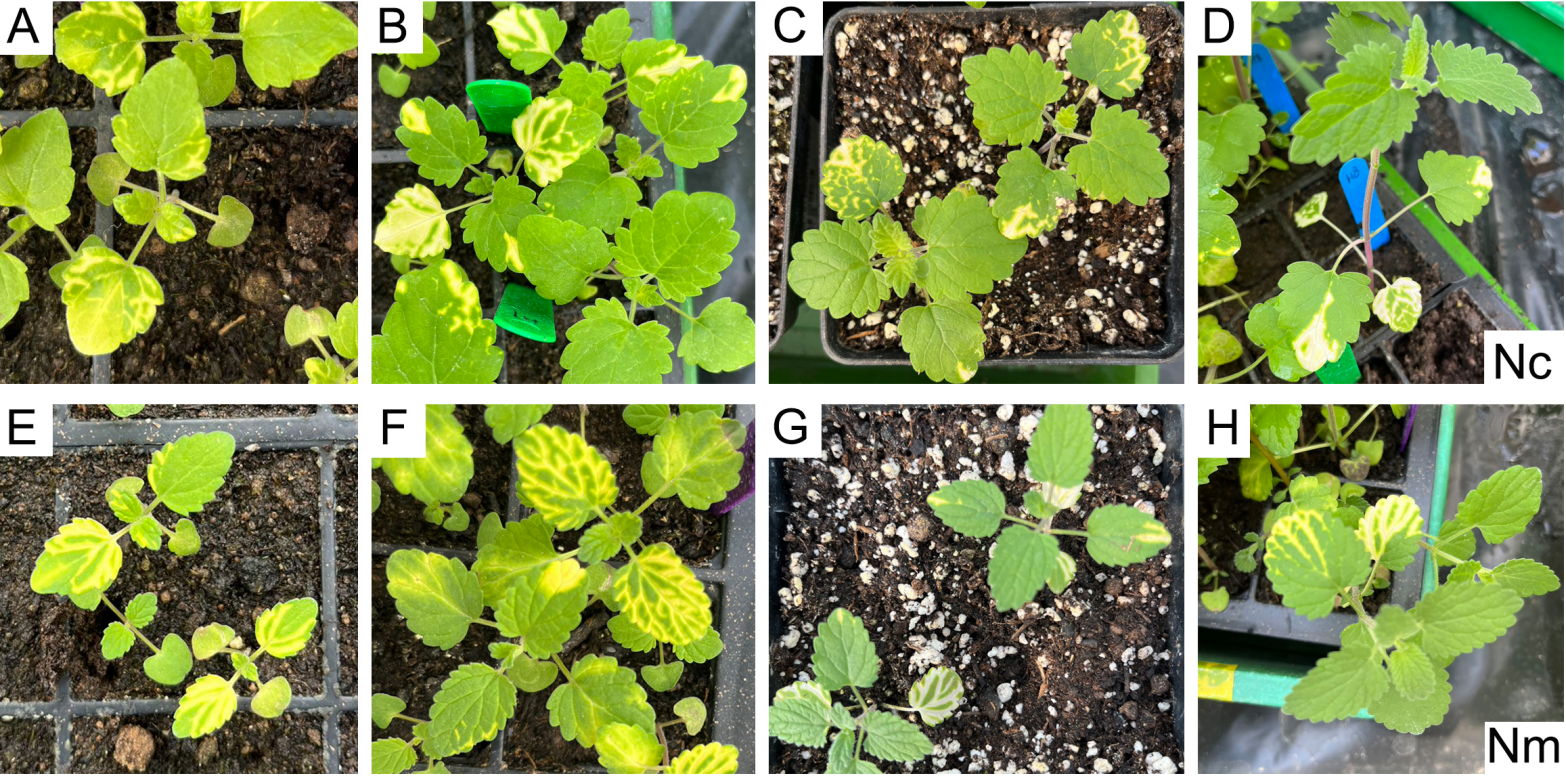
Evaluation of the spreading range and period. **(A–D)** *Nepeta cataria* (Nc). **(E–H)** *Nepeta mussinii* (Nm). **(A–H)** represent 1, 2, 3, and 4 weeks after Virus-induced gene silencing (VIGS), respectively.

The spread extent of chlorosis varies significantly in different plant species, ranging from a single pair of leaves to the whole plant. The VIGS plants were constantly monitored to determine the optimal time for collecting VIGS materials. After infiltration of the cotyledons, the proliferation of chlorosis was only observed in the first and second pairs of true leaves in *N. cataria* (Nc), but not the subsequently emerging leaves during three to four WAVs (**Figures 3A–D**). A closely related species, *N. mussinii* (Nm), was also investigated to see whether cotyledon-based VIGS is applicable to other plant species. Similar results were gained: chlorosis occurred in the first pair of true leaves in one WAV and then extended to the second pair in two WAVs but ceased to expand in three to four WAVs (**Figures 3E–H**). These results illustrated that the VIGS-induced repression of gene expression could propagate from cotyledon to the first and second pairs of true leaves without any further expansion in *N. cataria* and *N. mussinii*. The entire process of cotyledon-based VIGS could be completed within 3 weeks from seed germination to sample collection. Additionally, the cotyledon-based VIGS functioned effectively in both *N. cataria* and *N. mussinii*, indicating its potential application in a diverse range of plant species.

### The VIGS efficiency in *N. cataria* was higher than in *N. mussinii*

To detect the efficiency of the cotyledon-based VIGS, the experiments were conducted with different infiltration times in *N. cataria* and *N. mussinii*. In *N. cataria*, when the infiltration was performed in the cotyledons of seedlings 7 days after sowing (DAS), the mean VIGS efficiency was 84.4% by comparing the chlorotic ones divided by the total infiltration amount. However, when the infiltration was performed on nine DAS, the VIGS efficiency decreased. In comparison, the mean efficiency was 47.7% in seven DAS batches and 36.7% in nine DAS batches in *N. mussinii*, which was lower than that of *N. cataria* (**Table 1**). Therefore, the VIGS efficiency was diverse among different plant species, even when they shared a close phylogenetic relationship. Seven DAS was recommended as a better infiltration time since the VIGS efficiency showed a decreasing trend in nine DAS batches.

**Table 1:** Assessment of cotyledon-based VIGS efficiency.

Species	Infiltration time	Chlorosis no.	Infiltration no.	VIGS efficiency %
*Nepeta cataria* (Nc)	7 DAS	13	15	84.4 ± 2.8
		23	27	
		18	22	
	9 DAS	15	19	75.1 ± 3.8^a^
		20	28	
		18	24	
*Nepeta mussinii* (Nm)	7 DAS	20	40	47.7 ± 2.9
		17	35	
		12	27	
	9 DAS	6	15	36.7 ± 3.4^a^
		7	19	
		7	21	

VIGS efficiency indicates mean ± SD.

VIGS, virus-induced gene silencing; DAS, days after sowing.

a Relative to the corresponding VIGS efficiency of 7 DAS and *p*-value <0.05.

### The visual marker *ChlH* aided in gene function validation

**Figure 4:**
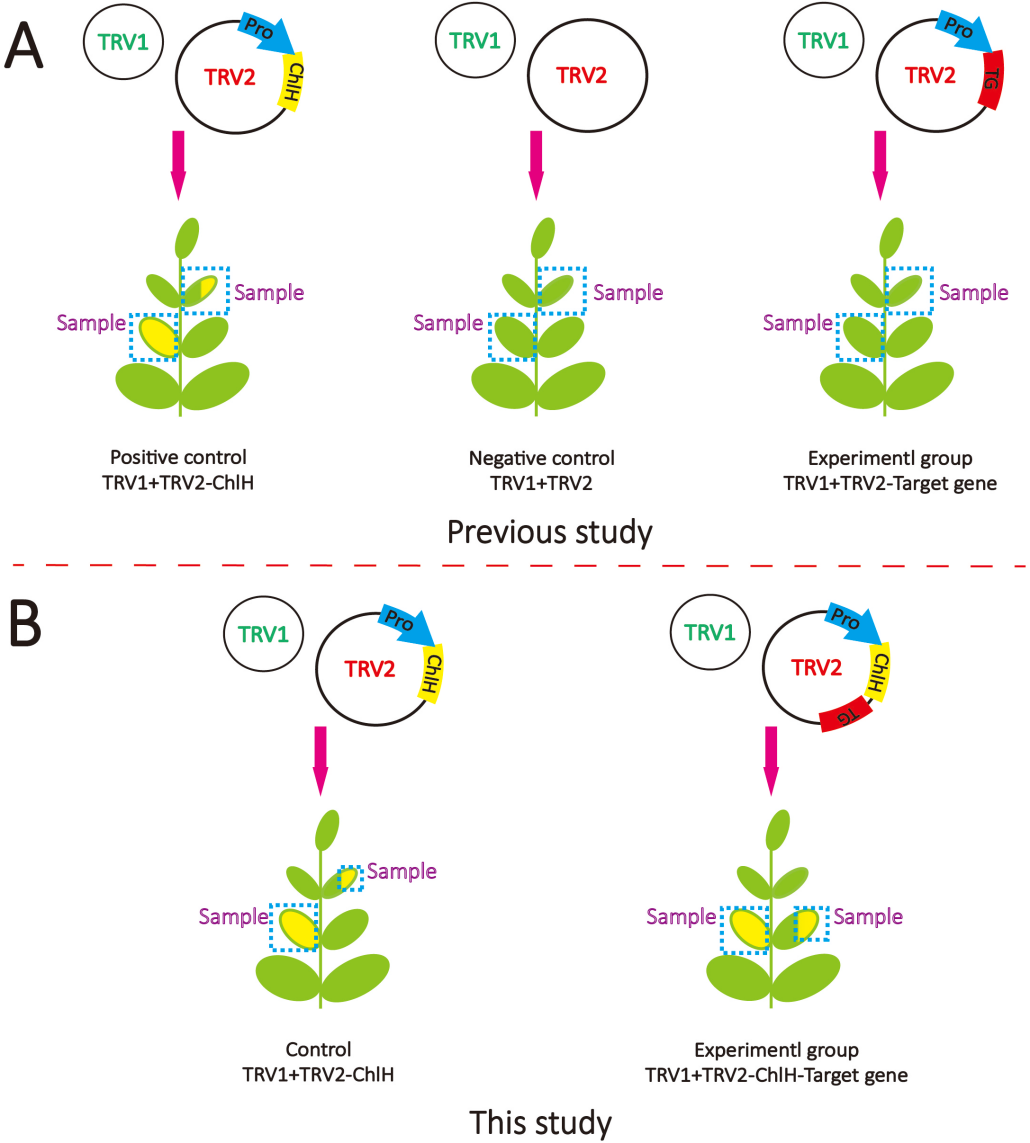
Schematic diagram of virus-induced gene silencing (VIGS) methods. **(A)** The VIGS method is commonly used in previous studies. TRV1 contains essential components for virus assembly, which is always co-transformed with TRV2, which contains the fragments of target genes. **(B)** The VIGS method was used in this study. The fragments of *ChlH* and target gene (TG) were inserted in TRV2 separately under the driving of one promoter (Pro).

The area and time period of gene silencing of VIGS exhibit variability among species and methods. Take *ChlH* for example; chlorosis can appear in the different phyllotaxes or leaf parts (**Figure 4B**). A marker can be useful to guide sampling for the identification of gene function. Previously, *ChlH* was independently employed as a positive control to validate the VIGS function well, and sampling positions of target genes were roughly approximated based on the positive control (**Figure 4A**). In this study, fragments of the target genes and the visual marker *ChlH* were successively co-constructed into TRV2 (**Figure 4B**). The silencing of the target gene and *ChlH* would be expected to happen in the same area, thus enabling the use of chlorosis of *ChlH* as a guiding indicator. GES and G8H are two key enzymes in nepetalactone biosynthesis. The role of *GES* has been characterized both *in vitro* and *in vivo* and was chosen as a positive control.

**Figure 5:**
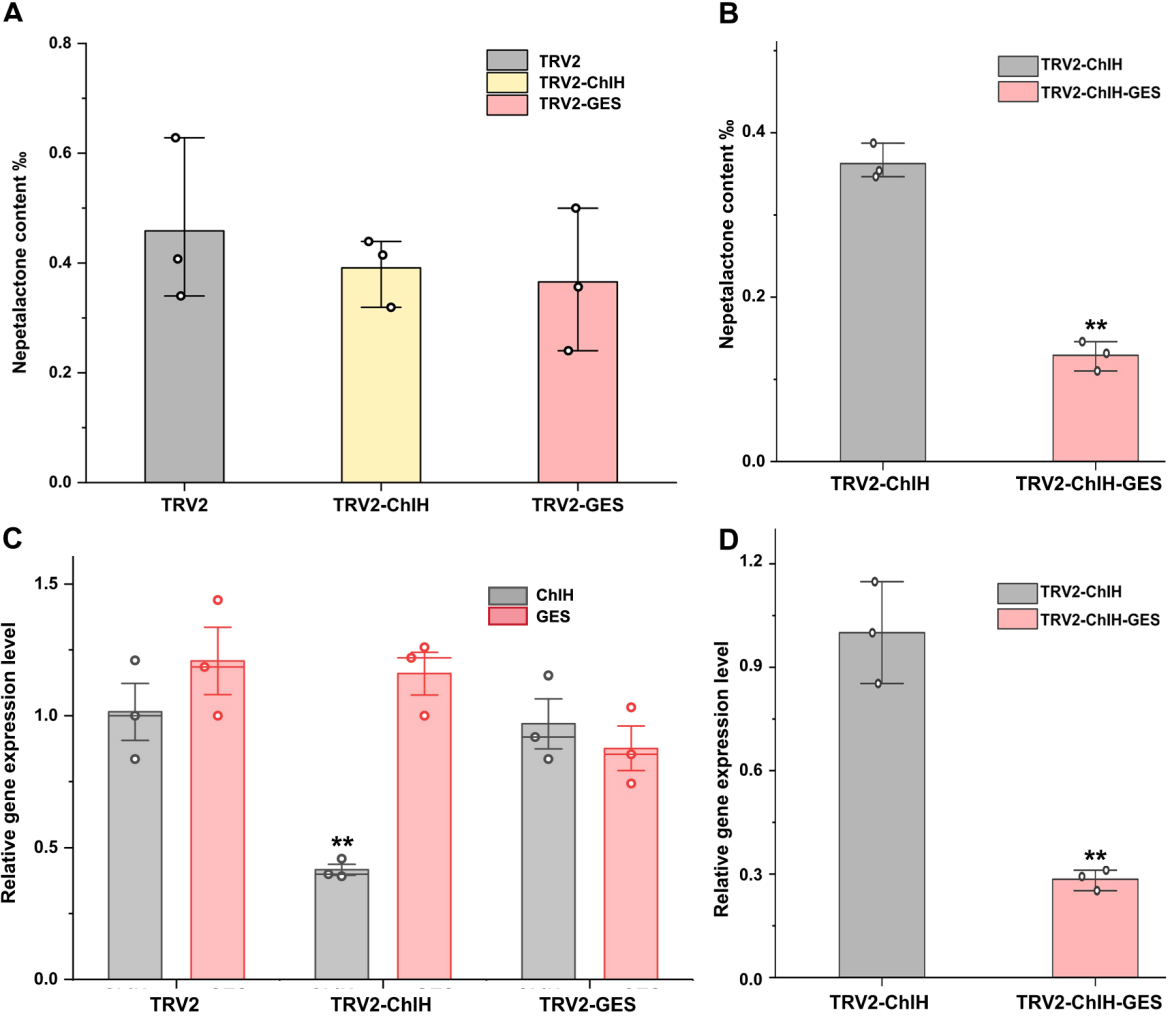
Assessment of the impact of visual marker on elucidating gene function in virus-induced gene silencing (VIGS). Panels **(A, C)** were without indicator; the content of *trans*–*cis* nepetalactone **(A)** and the expression level of *ChlH* and *GES* **(C)** in the first and second true leaves were measured. TRV2 empty vector was used as the negative control group. In panels **(B, D)**, the target gene’s fragment was co-expressed with the visual marker *ChlH*. After *GES* gene interference by VIGS, the content of *trans*–*cis* nepetalactone **(B)** and *GES* expression level **(D)** in the chlorosis part were detected. TRV2-ChlH was used as the control group. Error bars were the standard error (n = 3 or 4). ** above the bracket indicates *p*-value <0.01 in *t*-test.

To assess the impact of the visual marker on elucidating gene function, the entire leaves of the first and second true leaves were sampled following the method of a previous study (**Figures 4A**, **5A, C**). In contrast, only the chlorotic regions were harvested in the visual-marker batch (**Figures 4B**, **5B, D**). Although the expression level of *GES* and the content of *trans*–*cis* nepetalactone slightly decreased in comparison to the control, no significant difference was observed (**Figures 5A, C**). Conversely, with the assistance of *ChlH* during sampling, both *GES* expression level and *trans*–*cis* nepetalactone amount were substantially reduced (**Figures 5B, D**). This indicates that the visual marker is a great “chaperone” in uncovering gene function.

**Figure 6:**
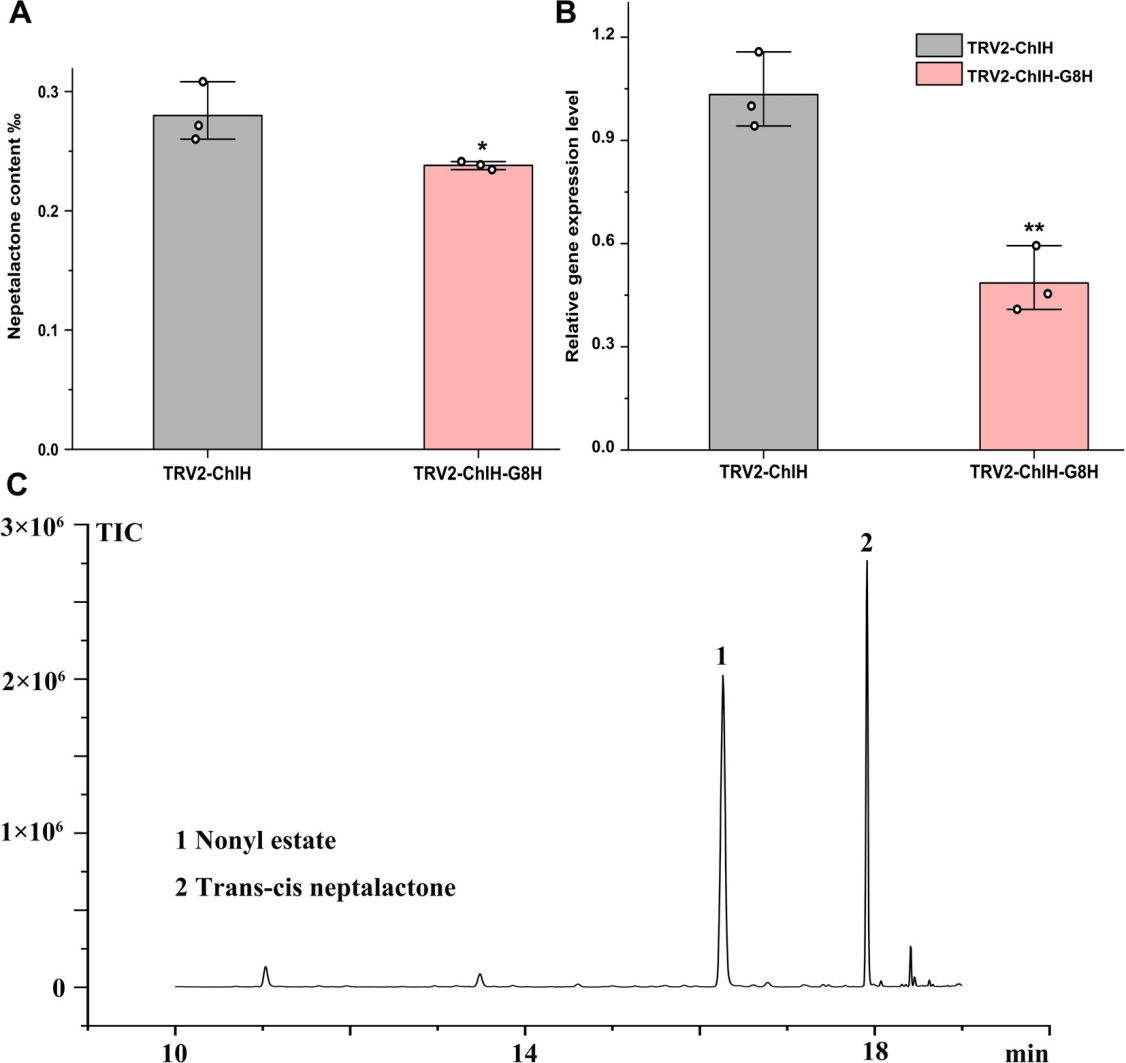
Characterization of the function of *G8H in vivo* by virus-induced gene silencing (VIGS). The content of *trans*–*cis* nepetalactone **(A)** and *G8H* expression level **(B)** were measured in the cotyledon-based visual VIGS experiment. **(C)** Representative gas chromatography–mass spectrometry (GC–MS) diagram from the control TRV2-ChlH. Nonyl acetate was used as the internal standard. Error bars were the standard error (n = 3). * and **above the bracket indicate *p* value < 0.05 and 0.01 respectively in *t* test.

*G8H* is proposed to mediate the conversion from geraniol to 8-hydroxygeraniol on the nepetalactone biosynthesis pathway; however, its endogenous role has not yet been validated. After interfering with the expression of *G8H* by VIGS, the content of *trans*–*cis* nepetalactone and the expression level of *G8H* were slightly diminished by 15% and 54%, respectively (**Figure 6**). Although the decrease in nepetalactone content was not large, the reduction in *G8H* expression and nepetalactone production was statistically significant (p < 0.05). These findings corroborated the involvement of *G8H* in nepetalactone biosynthesis in plants and demonstrated that cotyledon-based visual VIGS was valid in elucidating gene function in *N. cataria*.

## Discussion

Virus-induced gene silencing is a remarkable tool for gene function studies in plants recalcitrant to genetic transformation. Numerous methods, such as leaf injection, spray inoculation, and vacuum infiltration, have been devised to conduct agrobacteria-mediated VIGS in diverse plant species (Ratcliff et al., 2001; Liu et al., 2002; Zhang et al., 2017), but limitations still exist, including the reliance on equipment in spray inoculation and vacuum infiltration and long waiting period for plant growth. Here, we presented a cotyledon-based visual VIGS system in *N. cataria* with the advantages of a short VIGS period, good VIGS efficiency, highly effective gene function validation through precise sampling with visual indicator, and adaptable potential in other plant species like *N. mussinii*.

The period of VIGS varies significantly according to the different methods. It can take some time for the plant to grow to be fit for infiltration. Leaf injection is usually executed on true leaves like the fully expanded leaves of 3-week-old *Nicotiana benthamiana* or *Solanum lycopersicum* (Ratcliff et al., 2001; Liu et al., 2002). The onset and persistence of chlorosis also differ. In tobacco, the chlorotic phenotype emerged 10 days post infiltration (dpi) and ended in the new leaves after 28 dpi (Ratcliff et al., 2001). Similarly, in tomato, the phenotype appeared at 10 dpi and lasted for approximately 1 month (Liu et al., 2002). In *Euphorbia peplus*, chlorosis sustained from 5 to 40 dpi (Czechowski et al., 2022), while in cotton, the phenotype lasted from 2 to 3 weeks after infiltration to the flowering stage (Si et al., 2022). Palmer established the wounded node VIGS method in *N. cataria*, which encompassed steps like cutting branches, rooting, infection by wounding nodes, and sampling. The whole procedure was estimated to span 8–11 weeks including 5 weeks for plant growth (cutting and rooting) and 3–6 weeks for chlorosis appearance before sample collection. The approach here took only 1 week for germination and 2 weeks for chlorosis and sampling. Consequently, the entire cotyledon-based VIGS system in *N. cataria* was 3 weeks, which was considerably faster than the previous methods and independent of facilities.

The efficiency is critical to the applications of VIGS like gene function validation and pest defense. The concentrations of the infiltration solution contribute much to the VIGS efficiency. When the optical density at 600 nm (OD_600_) was 1.2, efficiency was the highest in tea (*Camellia sinensis* [L.] O. Kuntze) (Li et al., 2023), whereas in *N. benthamiana*, the optimal efficiency was observed with an OD_600_ of 0.8 and the lowest with OD_600_ of 0.1 (Li et al., 2024). The VIGS efficiency varies much across different plant species even utilizing the same infiltration approach. As in our case, the efficiency was lower in *N. mussinii* compared to *N. cataria*. Similar results were reported in 19 soybean genotypes, where the silencing rate was 100% in PI 567301B, while it was 0% in 10 other genotypes such as Williams (Gedling et al., 2018).

The VIGS methods are continuously being refined to attain higher efficiency. A root wounding immersion method was applied in *N. benthamiana* and tomato, resulting in a silencing rate of 95%–100% (Li et al., 2024). The extracts from agroinfiltrated *N. benthamiana* leaves were developed for soybean VIGS infiltration; the apical puncture inoculation method achieved 60% efficiency compared with 12.5% by rub-inoculation method in the Jack genotype soybean (Gedling et al., 2018). In *Nepeta*, the wounded node method was established, but the efficiency remained relatively low (between 25% and 32%), and the chlorosis phenotype was often confined to the vicinity of the infection site (Palmer et al., 2022). In our case, the cotyledon-based VIGS achieved an average efficiency of 84.4%, and the phenotype reliably spread to the first two pairs of true leaves. Overall, we provided an easy yet effective cotyledon-based VIGS and verified the role of *G8H* in nepetalactone synthesis in *N. cataria*, which will significantly facilitate the gene functional analysis in *Nepeta* and holds the potential to be applied for other purposes in many plants.

## Data Availability

The original contributions presented in the study are included in the article/**Supplementary Material**. Further inquiries can be directed to the corresponding authors.

## Supplementary material

Supplementary Figure 1 Tissue expression pattern of *CHIH* reporter genes in *Nepeta*. The samples from ‘mature leaf, closed flower buds, immature leaf, open flowers, petiole, root, stem’ were shown as ‘ML, CFB, IML, OF, P, R, S’ for short.

Supplementary Data Sheet 1 The cDNA sequences of Nepeta genes.

Supplementary Data Sheet 2 Primers for vector construction and qRT-PCR analysis.

Supplementary Data Sheet 3 The protein sequences of ChlHs used for phylogenetic analysis.

## References

[B1] Boachon B. Buell C. R. Crisovan E. Dudareva N. Garcia N. Godden G.. (2018). Phylogenomic mining of the mints reveals multiple mechanisms contributing to the evolution of chemical diversity in Lamiaceae. Mol. Plant 11, 1084–1096. doi: 10.1016/j.molp.2018.06.002 29920355

[B2] Carbonell A. Carrington J. C. (2015). Antiviral roles of plant ARGONAUTES. Curr. Opin. Plant Biol. 27, 111–117. doi: 10.1016/j.pbi.2015.06.013 26190744 PMC4618181

[B3] Chen C. Wu Y. Li J. Wang X. Zeng Z. Xu J.. (2023). TBtools-II: A “one for all, all for one” bioinformatics platform for biological big-data mining. Mol. Plant 16, 1733–1742. doi: 10.1016/j.molp.2023.09.010 37740491

[B4] Cheuk A. Houde M. (2019). A new barley stripe mosaic virus allows large protein overexpression for rapid function analysis. Plant Physiol. 180, 2306–2311. doi: 10.1104/pp.19.00759 29269575 PMC5841696

[B5] Czechowski T. Forestier E. Swamidatta S. H. Gilday A. D. Cording A. Larson T. R.. (2022). Gene discovery and virus-induced gene silencing reveal branched pathways to major classes of bioactive diterpenoids in Euphorbia peplus. Proc. Natl. Acad. Sci. United States America 119, e2203890119. doi: 10.1073/pnas.2203890119 PMC9173813 35584121

[B6] Gedling C. R. Ali E. M. Gunadi A. Finer J. J. Xie K. Liu Y.. (2018). Improved apple latent spherical virus-induced gene silencing in multiple soybean genotypes through direct inoculation of agro-infiltrated Nicotiana benthamiana extract. Plant Methods 14, 19–29. doi: 10.1186/s13007-018-0286-7 29527233 PMC5838930

[B7] Jiang Z. Q. Zhao Q. Q. Bai R. Y. Yu R. N. Diao P. F. Yan T.. (2021). Host sunflower-induced silencing of parasitism-related genes confers resistance to invading Orobanche cumana. Plant Physiol. 185, 424–440. doi: 10.1093/plphys/kiaa018 33721890 PMC8133596

[B8] Lei J. F. Dai P. H. Li Y. Zhang W. Q. Zhou G. T. Liu C.. (2021). Heritable gene editing using FT mobile guide RNAs and DNA viruses. Plant Methods 17, 20. doi: 10.1186/s13007-021-00719-4 33596981 PMC7890912

[B9] Li T. D. Yang X. P. Yu Y. Si X. M. Zhai X. W. Zhang H. W.. (2018). Domestication of wild tomato is accelerated by genome editing. Nat. Biotechnol. 36, 1160–1163. doi: 10.1038/nbt.4273 30272676

[B10] Li G. D. Li Y. Yao X. Z. Lu L. T. (2023). Establishment of a virus-induced gene-silencing (VIGS) system in tea plant and its use in the functional analysis of CsTCS1. Int. J. Mol. Sci. 24, 392–406. doi: 10.3390/ijms24010392 PMC9820744 36613837

[B11] Li X. Y. Tao N. Xu B. Xu J. Q. Yang Z. A. Jiang C. Q.. (2024). Establishment and application of a root wounding-immersion method for efficient virus-induced gene silencing in plants. Front. Plant Sci. 15. doi: 10.3389/fpls.2024.1336726 PMC11066161 38708388

[B12] Lichman B. R. Kamileen M. O. Titchiner G. R. Saalbach G. Stevenson C. E. M. Lawson D. M.. (2019). Uncoupled activation and cyclization in catmint reductive terpenoid biosynthesis. Nat. Chem. Biol. 15, 71–79. doi: 10.1038/s41589-018-0185-2 30531909 PMC6513753

[B13] Lichman B. R. Godden G. T. Hamilton J. P. Palmer L. Kamileen M. O. Zhao D. Y.. (2020). The evolutionary origins of the cat attractant nepetalactone in catnip. Sci. Adv. 6, eaba0721. doi: 10.1126/sciadv.aba0721 32426505 PMC7220310

[B14] Liu N. Xie K. Jia Q. Zhao J. P. Chen T. Y. Li H. G.. (2016). Foxtail mosaic virus-induced gene silencing in monocot plants. Plant Physiol. 171, 1801–1807. doi: 10.1104/pp.16.00010 27225900 PMC4936545

[B15] Liu Y. L. Schiff M. Dinesh-Kumar S. P. (2002). Virus-induced gene silencing in tomato. Plant J. 31, 777–786. doi: 10.1046/j.1365-313X.2002.01394.x 12220268

[B16] Matzke M. A. Mosher R. A. (2014). RNA-directed DNA methylation: an epigenetic pathway of increasing complexity. Nat. Rev. Genet. 15, 394–408. doi: 10.1038/nrg3683 24805120

[B17] Misra R. C. Sharma S. Sandeep Garg A. Chanotiya C. S. Ghosh S. (2017). Two CYP716A subfamily cytochrome P450 monooxygenases of sweet basil play similar but nonredundant roles in ursane- and oleanane-type pentacyclic triterpene biosynthesis. New Phytol. 214, 706–720. doi: 10.1111/nph.2017.214.issue-2 28967669

[B18] Palmer L. Chuang L. Siegmund M. Kunert M. Yamamoto K. Sonawane P.. (2022). *In vivo* characterization of key iridoid biosynthesis pathway genes in catnip. Planta 256. doi: 10.1007/s00425-022-04012-z PMC9556426 36222913

[B19] Ratcliff F. Martin-Hernandez A. M. Baulcombe D. C. (2001). Tobacco rattle virus as a vector for analysis of gene function by silencing. Plant J. 25, 237–245. doi: 10.1046/j.0960-7412.2000.00942.x 11169199

[B20] Rössner C. Lotz D. Becker A. (2022). VIGS goes viral: how VIGS transforms our understanding of plant science. Annu. Rev. Plant Biol. 73, 703–728. doi: 10.1146/annurev-arplant-102820-020542 35138878

[B21] Shi G. Y. Hao M. Y. Tian B. M. Cao G. Q. Wei F. Xie Z. Q. (2021). A methodological advance of tobacco rattle virus-induced gene silencing for functional genomics in plants. Front. Plant Sci. 12, 671091–671106. doi: 10.3389/fpls.2021.671091 34149770 PMC8212136

[B22] Si Z. F. Wu H. T. Tian Y. Zhang Z. Y. Zhang T. Z. Hu Y. (2022). Visible gland constantly traces virus-induced gene silencing in cotton. Front. Plant Sci. 13. doi: 10.3389/fpls.2022.1020841 PMC9523728 36186026

[B23] Smit S. J. Lichman B. R. (2022). Plant biosynthetic gene clusters in the context of metabolic evolution. Natural Product Rep. 39, 1465–1482. doi: 10.1039/D2NP00005A PMC9298681 35441651

[B24] Taochy C. Gursanscky N. R. Cao J. L. Fletcher S. J. Dressel U. Mitter N.. (2017). A genetic screen for impaired systemic RNAi highlights the crucial role of DICER-LIKE 2. Plant Physiol. 175, 1424–1437. doi: 10.1104/pp.17.01181 28928141 PMC5664484

[B25] Uenoyama R. Miyazaki T. Hurst J. L. Beynon R. J. Adachi M. Murooka T.. (2021). The characteristic response of domestic cats to plant iridoids allows them to gain chemical defense against mosquitoes. Sci. Adv. 7, eabd9135. doi: 10.1126/sciadv.abd9135 33523929 PMC7817105

[B26] Wu H. H. Li B. S. Iwakawa H. O. Pan Y. J. Tang X. L. Ling-hu Q. Y.. (2020). Plant 22-nt siRNAs mediate translational repression and stress adaptation. Nature 581, 89–93. doi: 10.1038/s41586-020-2231-y 32376953

[B27] Wu C. J. Jia L. L. Goggin F. (2011). The reliability of virus-induced gene silencing experiments using tobacco rattle virus in tomato is influenced by the size of the vector control. Mol. Plant Pathol. 12, 299–305. doi: 10.1111/j.1364-3703.2010.00669.x 21356001 PMC6640492

[B28] Yao M. Q. Chen W. W. Kong J. H. Zhang X. L. Shi N. N. Zhong S. L.. (2020). METHYLTRANSFERASE1 and ripening modulate vivipary during tomato fruit development. Plant Physiol. 183, 1883–1897. doi: 10.1104/pp.20.00499 32503901 PMC7401104

[B29] Zhang J. Yu D. S. Zhang Y. Liu K. Xu K. D. Zhang F. L.. (2017). Vacuum and co-cultivation agroinfiltration of (germinated) seeds results in tobacco rattle virus (TRV) mediated whole-plant virus-induced gene silencing (VIGS) in wheat and maize. Front. Plant Sci. 8. doi: 10.3389/fpls.2017.00393 PMC5360694 28382049

