# Corrigendum: Integrating cotyledon-based virus-induced gene silencing with visual marker promises a rapid, highly effective validation of gene functions in *Nepeta cataria*

**DOI:** 10.3389/fpls.2025.1596651

**Published:** 2025-04-03

**Authors:** Zongxia Yu, Ruo Lv, Bo Hong, Lei Yang

**Affiliations:** ^1^ Jiangxi Key Laboratory for Sustainable Utilization of Chinese Materia Medica Resources, Lushan Botanical Garden, Chinese Academy of Sciences, Jiujiang, Jiangxi, China; ^2^ Shanghai Key Laboratory of Plant Functional Genomics and Resources, Shanghai Chenshan Botanical Garden, Shanghai, China

**Keywords:** VIGS, cotyledon infiltration, visual marker, G8H, nepetalactone, catmint

In the published article, there was an error in the **Funding** statement. The funding statement for Science Fund for Distinguished Young Scholars of Jiangxi Province was displayed as “No. 20224ACB21500”. The correct **Funding** statement appears below.

“The author(s) declare financial support was received for the research, authorship, and/or publication of this article. ZY acknowledges the National Natural Science Foundation of China (No. 32360111), Program of China Scholarship Council (No. 202008210144), Science Fund for Distinguished Young Scholars of Jiangxi Province (No. 20224ACB215004), Double Thousand Plan of Jiangxi Province (No. JXSQ2023101106), Key Research and Development Program of Jiangxi Province (No. 20243BBI91010), and Open Project Program of Shanghai Key Laboratory of Plant Functional Genomics and Resources (No. PFGR202303). LY acknowledges Yunnan Revitalization Talent Support Program “Top Team” Project (Grant No. 202305AT350001), Science and Technology Commission of Shanghai Municipality (YDZX20223100001003 and YDZX20243100004002), and the Special Fund for Shanghai Landscaping Administration Bureau Program (Grant No. G232401).”

The authors apologize for this error and state that this does not change the scientific conclusions of the article in any way. The original article has been updated.

