# Supplementary figures and images for: Integrating cotyledon-based virus-induced gene silencing with visual marker promises a rapid, highly effective validation of gene functions in Nepeta cataria
Source: Front Plant Sci. 2025 Jan 21;15:1514614. doi: 10.3389/fpls.2024.1514614 (PMC11790630; doi:10.3389/fpls.2024.1514614)

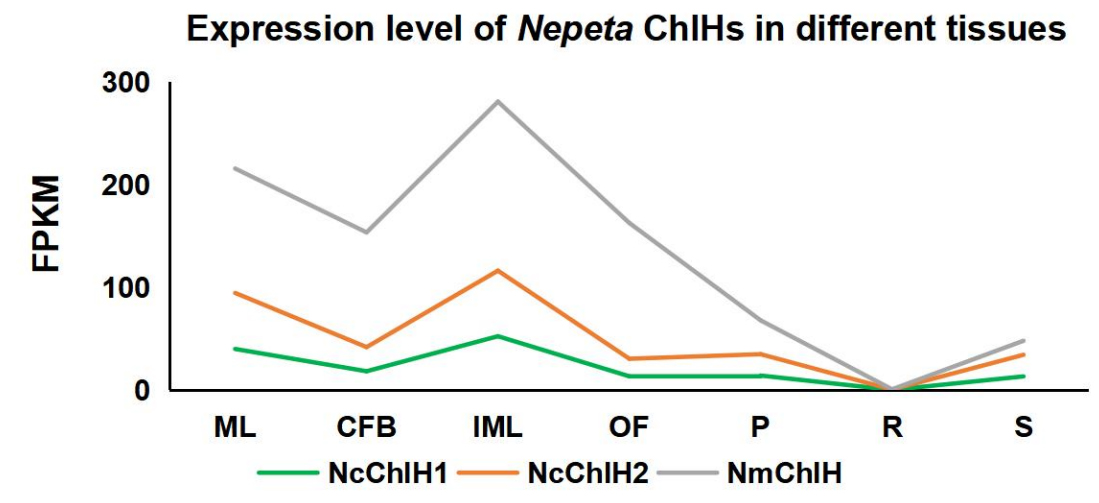

Supplement: Supplementary Figure 1 — Tissue expression pattern of CHIH reporter genes in Nepeta. The samples from ‘mature leaf, closed flower buds, immature leaf, open flowers, petiole, root, stem’ were shown as ‘ML, CFB, IML, OF, P, R, S’ for short. [file Image1.jpeg]
