# Supplementary material for: Integrating cotyledon-based virus-induced gene silencing with visual marker promises a rapid, highly effective validation of gene functions in Nepeta cataria
Source: Front Plant Sci. 2025 Jan 21;15:1514614. doi: 10.3389/fpls.2024.1514614 (PMC11790630; doi:10.3389/fpls.2024.1514614)
Supplement: Supplementary Data Sheet 1 — The cDNA sequences of Nepeta genes. [file DataSheet1.docx]

>NcChlH1

ATGGCTGCTTCTTTGGTTTCATCGCCATTTACACTTCCAAAATCCAAAACAGTAAACCTTTCATCACTCTCACAAAAGCATTACCTCCTCCACTCTTTCCTYCCCAAGAGAATCACCAAAACCAACATAAATTCATCCCAGAAATTCAAATGCAATGCCATTGGCAACGGTCTCTTCACTCAGACCACGCAAGAAGTCCGTAGAATCGTGCCCGAGAAATCGAACCTCACAACGGTTAAGGTTGTCTACGTGGTGCTAGAAGCTCAATACCAATCATCCCTCACAGCTGCAGTTCAAGCTCTCAACAAAAATGGAGAGTATGCTTCATTTGAGCTTGTGGGCTACTTGGTTGAGGAGCTGAGAGATAAGGACAACTACGAGAGCTTCTGCAAGGATCTCGAAGACGCCAATATCTTCATCGGATCACTCATTTTCGTGGAGGAGTTGGCGTTGAAGGTCAAGGCTGCTGTGGAGAAGGAGAGAGAGAGGCTTGATGCTGTGCTGGTTTTCCCATCAATGCCTGAGGTGATGAGATTGAACAAGCTTGGATCTTTCAGCATGTCGCAGTTGGGGCAGTCGAAAAGCCCCTTCTTTCAGCTGTTCAAGAAGAAGGGCAAATCTAATGCAGCCTTTGCAGACAGCATGTTGAAGCTTGTAAGAACTTTACCTAAAGTCTTGAAGTATTTGCCCAGTGATAAGGCTCAAGATGCAAGGTTGTACATTCTCAGCCTCCAGTTTTGGCTTGGTGGATCGCCTGATAACCTGATGAACTTCTTGAAGATGATCTCTGGATCATATGTGCCAGCGTTGAAACAGGMGAAAATCGAGTATTCTGATCCAGTTCTGTATTTTGATAATGGGATTTGGCACCCTTTGGCTCCCTGTATGTATGATGATGTGAAGGAGTATTTGAATTGGTATGCAACGAGAAGGGATGCTAATGAGCAGCTCAAGAGCAAGAATGCACCTGTGGTTGGGCTTGTTTTGCAGAGGAGTCATATTGTGACTGGTGATGATAGTCACTATGTTGCTGTGATCATGGAGCTCGAAGCTAAAGGGGCGAAGGTGATCCCGATCTTTGCTGGTGGCCTTGAYTTCTCTGGGCCGGTGGAGAAGTACTTCATTGATCCGATCACCAAGAAACCRATGATAAACTCAGTTGTGTCGTTGACTGGTTTTGCTCTTGTGGGAGGGCCCGCTAGGCAGGACCATCCGAGGGCAATCGAGGCCTTGATGAAGCTAGATGTGCCTTACATTGTGGCGCTGCCGTTGGTGTTCCAGACAACGGAGGAGTGGCTGAACAGCACGTTGGGATTGCACCCCATTCAGGTTGCTCTGCAAGTGGCTCTTCCTGAGCTTGATGGAGGCATGGAGCCTATTGTTTTCTCGGGGCGAGATCCAAGAACAGGGAAATCACATGCTCTTCACAAGAGGGTGGAGCAGCTCTGCACCAGAGCTATCAGATGGGCTGAACTCAAGAGGAAAACAAAGGCGGAAAAGAAGCTAGCAATCACCGTATTTAGCTTCCCACCAGACAAAGGCAATGTAGGCACTGCTGCTTACCTCAATGTCTTCTCTTCCATCTACTCTGTGCTCAAAGAGCTCAAGAAAGATGGCTACAACGTTGATGGCCTTCCAGAAACTTCAGAAGCCTTGATCGAAGAARTCATYCACGATAAGGAGGCCCAATTCAACAGCCCCAAYCTCAACGTAGTTCACAAGATGAGCGTCCGGGAATACCAGARTCTGACTCCTTACTCTACTGCTCTAGAAGAAAACTGGGGAAAGCCTCCAGGCAACTTGAACTCCGATGGTGAAAAYCTTCTTGTCTATGGCAAACAGTATGGAAACATCTTCATTGGTGTTCAGCCTACTTTTGGCTACGAGGGTGATCCTATGCGTCTTCTCTTCTCCAAATCCGCCAGCCCACACCACGGATTTGCTGCATACTACTCATATGTGGAGAAGATCTTTAAGGCYGACGCAGTTCTGCACTTTGGAACTCATGGTTCTCTTGAATTCATGCCTGGGAAACAAGTTGGCATGAGTGATGCCTGCTTCCCCGACAGTCTTATTGGAAACATCCCCAACATCTACTACTACGCCGCTAACAACCCGTCTGAGGCCACTGTTGCAAAGAGGCGTAGCTATGCGAATACTATCAGTTACCTGACTCCTCCAGCTGAGAATGCAGGTCTCTACAAAGGTCTGAAGCAGTTGAGCGAGCTTATTGCTTCTTACCAATCTCTCAAAGATTCTGGCCGTGGCCCTCAAATTGTGAGCTCTATTATCAGTACTGCTAGGCAGTGCAATCTTGATAAAGATGTGGTTCTTCCTGAAGACGGAGTCGAGCTCTCTCAAAAAGAGCGTGACTCTGTAGTGGGACAAGTGTATTCCAAGATTATGGAGATCGAATCGAGATTGCTACCTTGTGGCCTTCATGTCATTGGTGARCCTCCAACAGCAATGGAGGCAGTGGCTACACTGGTCAAYATCGCWGCATTGGATCGTGAAGAGGAACAGATTTCGTCTCTTCCTTCTATATTAGCKCAGACTGTGGGGAGAGARATTGAAGATATCTACAGAGGAAGTGATAAGGGTGTTCTRCGCGATGTGGAGCTCCTTAAACAGATCACTGAAGTATCTCGAGGTGCAATCAGTGCTTTTGTGGAAAGAAGCACCAACAGCAAAGGGCAAGTTGTTGAAGTAGCTGATAAGCTCACATCAATYCTTGGTTTTGGTGTAAACGAGCCATGGATTCAATACTTGTCGAACACCAAGTTTTACCGGGCTGACAGAGAAAAACTCAGAGTCTTGTTCGGATACTTAGGTGAATGCTTGAGACTGATTGTRRCTGATAACGAGGTGGGAAGCTTRAAACAAGCTTTGGAAGGGAAGTAYGTCGAGCCAGGGCCAGGTGGGGACCCRATCAGAAACCCGAAGGTGCTGCCTACAGGAAAGAACATCCATGCCTTGGACCCGCAGTCCATTCCAACAACTGCAGCTATGCAGAGTGCTAAAGTGGTGGTGGAGAGGCTACTCGAGAGGCAGAAGATCGATAATGGTGGAAAATATCCCGAGACAGTTGCTTTAGTACTATGGGGTACTGATAACATCAAGACATATGGTGAATCACTAGCTCAAGTTCTTTGGATGATTGGAGTTACACCGGTAACTGATGGGCTTGGGCGTGTTAACCGTGTGGAGATCGTGAGCCTTGAAGAGCTCGGAAGGCCGAGAATTGATGTCGTTGTCAACTGCTCCGGTGTCTTCAGAGACCTCTTCATCAATCAGATGAATCTTCTTGATAGRGCAGTGAAGATGGTAGCTGAGCTGGAYGAGCCGGAGGAGCAGAACTACGTGAGGAAACATGCATTGGAGCAAGCCAAAGAGCTGGGAGTTGAAGTCAGAGAAGCTGCCTCACGTATTTTCTCAAACGCCTCMGGCTCATACTCCTCCAACATCAATCTTGCTGTTGAGAACTCATCTTGGAATGATGARAAGCAGCTACAGGACATGTACTTGAGCAGAAAGTCGTTTGCATTCGACTCTGATGCCCCTGGTGTAGGCATGACGGAGAAACGAAAGATCTTTGAGATGGCTCTCAGCACAGCCGATGCCACCTTCCAGAATCTTGATTCCTCGGAGATATCTCTCACCGATGTCAGTCACTACTTCGATTCAGACCCGACTAATCTCGTACAAGGCCTCAGGAARGACGGGAAGAAGCCGAGTGCATACATTGCAGACACCACCACAGCTAATGCACAGGTGCGGACGCTGTCTGAGACAGTGAGGCTCGATGCAAGGACCAAGCTGTTGAATCCCAAGTGGTACGAGGGCATGCTGTCCAGTGGCTATGAGGGTGTTCGTGAGATTGAGAAACGTCTCACAAACACTGTGGGTTGGAGCGCAACTTCAGGACAAGTCGACAACTGGGTGTACGAGGAGGCCAACACAACATTCATCCAAGACGAGGAGATGCTGAACARGCTGATGAGCACGAACCCGAACTCGTTCAGGAAATTGCTWCAGACTTTCTTGGAGGCTAATGGAAGAGGATACTGGGAGACTAGTGAAGAAAACATTGAGAGGTTAAAGCAGTTGTACTCTGAAGTTGAAGACAAGATTGAAGGAATCGATCGTTAG

>NcChlH2

ATGGCTGCTTCTTTGGTTTCATCGCCATTTACACTTCCAAAATCCAAAACAGTAAACCTTTCATCACTCTCACAAAAGCATTACCTCCTCCACTCTTTCCTYCCCAAGAGAATCACCAAAACCAACATAAATTCATCCCAGAAATTCAAATGCAATGCCATTGGCAACGGTCTCTTCACTCAGACCACGCAAGAAGTCCGTAGAATCGTGCCCGAGAAATCGAACCTCACAACGGTTAAGGTTGTCTACGTGGTGCTAGAAGCTCAATACCAATCATCCCTCACAGCTGCAGTCCAAGCTCTCAACAAAAATGGAGAGTATGCTTCATTTGAGCTTGTGGGCTACTTGGTTGAGGAGCTGAGAGATAAGGACAACTACGAGAGCTTCTGCAAGGATCTCGAAGACGCCAATATCTTCATCGGATCACTCATTTTCGTGGAGGAGTTGGCGTTGAAGGTCAAGGCTGCTGTGGAGAAGGAGAGAGAGAGGCTTGATGCTGTGCTGGTTTTCCCATCAATGCCTGAGGTGATGAGATTGAACAAGCTTGGATCTTTCAGCATGTCGCAGTTGGGGCAGTCGAAAAGCCCCTTCTTTCAGCTGTTCAAGAAGAAGGGCAAATCTAATGCAGCCTTTGCAGACAGCATGTTGAAGCTTGTAAGAACTTTACCTAAAGTCTTGAAGTATTTGCCCAGTGATAAGGCTCAAGATGCAAGGTTGTACATTCTCAGCCTCCAGTTTTGGCTTGGTGGATCGCCTGATAACCTGATGAACTTCTTGAAGATGATCTCTGGATCATATGTGCCAGCGTTGAAACAGGMGAAAATCGAGTATTCTGATCCAGTTCTGTATTTTGATAATGGGATTTGGCACCCTTTGGCTCCCTGTATGTATGATGATGTGAAGGAGTATTTGAATTGGTATGCAACGAGAAGGGATGCTAATGAGCAGCTCAAGAGCAAGAATGCACCTGTGGTTGGGCTTGTTTTGCAGAGGAGTCATATTGTGACTGGTGATGATAGTCACTATGTTGCTGTGATCATGGAGCTCGAAGCTAAAGGGGCGAAGGTGATCCCGATCTTTGCTGGTGGCCTTGAYTTCTCTGGGCCGGTGGAGAAGTACTTCATTGATCCGATCACCAAGAAACCRATGATAAACTCAGTTGTGTCGTTGACTGGTTTTGCTCTTGTGGGAGGGCCCGCTAGGCAGGACCATCCGAGGGCAATCGAGGCCTTGATGAAGCTAGATGTGCCTTACATTGTGGCACTGCCGTTGGTGTTCCAGACAACGGAAGAGTGGCTGAACAGCACGTTGGGGTTGCATCCCATTCAGGTTGCTCTGCAAGTGGCTCTCCCTGAGCTTGATGGAGGCATGGAGCCTATTGTTTTCTCGGGGCGAGATCCAAGAACAGGGAAATCACATGCTCTTCACAAGAGGGTGGAGCAGCTCTGCACCAGAGCTATCAGATGGGCTGAACTCAAGAGGAAAACAAAGGCGGAAAAGAAGCTAGCAATCACTGTATTTAGCTTCCCACCAGACAAAGGCAATGTAGGAACTGCTGCTTACCTCAATGTCTTCTCTTCCATCTACTCTGTGCTCAAAGAGCTCAAGAAAGATGGCTACAACGTTGATGGCCTTCCAGAAACTTCAGAAGCCTTGATCGAAGAARTCATYCACGATAAGGAGGCCCAATTCAACAGCCCCAAYCTCAACGTAGTTCACAAGATGAGCGTCCGGGAATACCAGARTCTGACTCCTTACTCTACTGCTCTAGAAGAAAACTGGGGAAAGCCTCCAGGCAACTTGAACTCCGATGGTGAAAAYCTTCTTGTCTATGGCAAACAGTATGGAAACATCTTCATTGGTGTTCAGCCTACTTTTGGCTACGAGGGTGATCCTATGCGTCTTCTCTTCTCCAAATCCGCCAGCCCACACCACGGATTTGCTGCATACTACTCATATGTGGAGAAGATCTTTAAGGCYGACGCAGTTCTGCACTTTGGAACTCATGGTTCTCTTGAATTCATGCCTGGGAAACAAGTTGGCATGAGTGATGCCTGCTTCCCCGACAGTCTTATTGGAAACATCCCCAACATCTACTACTACGCYGCTAACAACCCGTCTGAGGCCACTGTTGCAAAGAGGCGTAGCTATGCGAATACTATCAGTTACCTGACTCCTCCAGCCGAGAATGCAGGCCTCTACAAAGGGCTGAAGCAGTTGAGCGAGCTGATTGCTTCTTACCAATCTCTCAAAGATTCGGGCCGTGGCCCTCAAATTGTGAGCTCTATTATCAGTACTGCTAGGCAGTGCAATCTTGATAAAGATGTGGTTCTTCCTGAAGAYGGAGTCGAGCTCTCTCAAAAAGAGCGTGACTCTGTAGTGGGACAAGTGTATTCCAAGATTATGGAGATCGAATCGAGATTGCTACCTTGTGGCCTTCATGTCATTGGTGARCCTCCAACAGCAATGGAGGCAGTGGCTACACTGGTCAAYATCGCWGCATTGGATCGTGAAGAGGAACAGATTTCGTCTCTTCCTTCTATATTAGCKCAGACTGTGGGGAGAGARATTGAAGATATCTACAGAGGAAGTGATAAGGGTGTTCTRCGCGATGTGGAGCTCCTTAAACAGATCACTGAAGTATCTCGAGGTGCAATCAGTGCTTTTGTGGAAAGAAGCACCAACAGCAAAGGGCAAGTTGTTGAAGTAGCTGATAAGCTCACATCAATYCTTGGTTTTGGTGTAAACGAGCCATGGATTCAATACTTGTCGAACACCAAGTTTTACCGGGCTGACAGAGAAAAACTCAGAGTCTTGTTCGGATACTTAGGTGAATGCTTGAGACTGATTGTRRCTGATAACGAGGTGGGAAGCTTRAAACAAGCTTTGGAAGGGAAGTAYGTCGAGCCAGGGCCAGGTGGGGACCCRATCAGAAACCCGAAGGTGCTGCCTACAGGAAAGAACATCCATGCCTTGGACCCGCAGTCCATTCCAACAACTGCAGCTATGCAGAGTGCTAAAGTGGTGGTGGAGAGGCTACTCGAGAGGCAGAAGATCGATAATGGTGGAAAATATCCCGAGACAGTTGCTTTAGTACTATGGGGTACTGATAACATCAAGACATATGGTGAATCACTAGCTCAAGTTCTTTGGATGATTGGAGTTACACCGGTAACTGATGGGCTTGGGCGTGTTAACCGTGTGGAGATCGTGAGCCTTGAAGAGCTCGGAAGGCCGAGAATTGATGTCGTTGTCAACTGCTCCGGKGTCTTCAGAGACCTCTTCATCAATCAGATGAATCTTCTTGATAGRGCAGTGAAGATGGTAGCTGAGCTGGAYGAGCCGGAGGAGCAGAACTACGTGAGGAAACATGCATTGGAGCAAGCCAAAGAGCTGGGAGTTGAAGTCAGAGAAGCTGCCTCACGTATTTTCTCAAACGCCTCMGGCTCATACTCCTCCAACATCAATCTTGCTGTTGAGAACTCATCTTGGAATGATGARAAGCAGCTACAGGACATGTACTTGAGCAGAAAGTCGTTTGCATTCGACTCTGATGCCCCCGGCGTAGGCATGACGGAGAAACGAAAGATCTTTGAGATGGCTCTCAGCACAGCAGATGCCACCTTCCAGAATCTTGATTCCTCAGAGATATCTCTCACCGATGTCAGTCACTACTTCGATTCAGACCCAACTAATCTCGTACAAGGCCTCAGGAARGACGGGAAGAAGCCGAGTGCATACATTGCAGACACCACCACAGCTAATGCACAGGTGCGGACGCTGTCTGAGACAGTGAGGCTCGATGCAAGGACCAAGCTGTTGAATCCCAAGTGGTACGAGGGCATGCTGTCCAGTGGCTATGAGGGTGTTCGTGAGATTGAGAAACGTCTCACAAACACTGTGGGTTGGAGCGCAACTTCAGGACAAGTCGACAACTGGGTGTACGAGGAGGCCAACACAACATTCATCCAAGACGAGGAGATGCTGAACAAGCTGATGAGCACGAACCCGAACTCGTTCAGGAAATTGCTWCAGACTTTCTTGGAGGCTAATGGAAGAGGATACTGGGAGACTAGTGAAGAAAACATTGAGAGGTTAAAGCAGTTGTACTCTGAAGTTGAAGACAAGATTGAAGGAATCGATCGTTAG

>NmChlH

ATGGCTGCTTCTTTGGTTTCATCGCCATTTACACTTCCAAAATCCAAAACAGTAAACCTTTCATCACTCTCACAAAAGCATTACCTCCTCCACTCTTTCCTCCCCAAGAGAATCGCCAAAACCAACATAAATTCATCCCAGAAATTCAAATGCAATGCCATTGGCAACGGTCTCTTCACTCAGACCACGCAAGAAGTYCGTAGAATCGTGCCCGAGAAATCGAACCTCACAACGGTTAAGGTTGTCTACGTGGTGCTAGAAGCTCAATAYCARTCATCCCTCACAGCTGCWGTCCAAGCTCTCAACAAAAATGGAGAGTATGCTTCATTTGAGCTTGTGGGMTACTTGGTTGAGGAGCTGAGAGATAAGGACAACTACGAGAGCTTCTGCAAGGATCTCGAAGACGCCAATATCTTCATCGGATCACTCATTTTCGTGGAGGAGTTGGCGTTGAAGGTCAAGGCTGCTGTGGAGAAGGAGAGAGAGAGGCTTGATGCTGTGCTGGTTTTCCCATCAATGCCTGAGGTGATGAGATTGAACAAGCTTGGATCTTTCAGCATGTCGCAGTTGGGGCAGTCGAAAAGCCCCTTCTTTCAGCTGTTCAAGAAGAAGGGCAAATCTAATGCAGCCTTTGCAGACAGCATGTTGAAGCTTGTAAGAACTTTACCTAAAGTCTTGAAGTATTTGCCCAGTGATAAGGCTCAAGATGCAAGGTTGTACATTCTCAGCCTCCAGTTTTGGCTTGGTGGATCGCCTGATAACCTGATGAACTTTTTGAAGATGATCTCTGGATCATATGTGCCAGCGTTGAAACAGGCGAAAATCGAGTATTCTGATCCAGTTCTGTATTTTGATAATGGGATTTGGCACCCTTTGGCTCCCTGTATGTATGATGATGTGAAGGAGTATTTGAATTGGTATGCAACGAGAAGGGATGCTAATGAGCAGCTCAAGAGCAAGAATGCACCTGTGGTTGGGCTTGTTTTGCAGAGRAGTCATATTGTGACTGGTGATGATAGTCACTACGTTGCTGTGATCATGGAGCTCGAAGCTAAAGGGGCGAAGGTGATCCCGATCTTTGCTGGTGGCCTTGACTTCTCTGGGCCGGTGGAGAAGTACTTCATTGATCCGATCACCAAGAAACCGATGATAAACTCRGTTGTGTCGTTGACTGGTTTTGCTCTTGTGGGAGGGCCYGCTAGGCAGGACCATCCSAGGGCAATCGAGGCCTTGATGAAGCTAGATGTGCCTTACATTGTGGCGCTGCCGCTGGTGTTCCAGACAACGGAGGAATGGCTGAACAGCACGTTGGGGTTGCACCCCATTCAGGTTGCTCTGCAAGTGGCTCTCCCTGAGCTTGATGGAGGCATGGAGCCTATTGTTTTCTCGGGGCGAGATCCAAGAACAGGGAAATCACATGCTCTTCACAAGAGGGTGGAGCAGCTCTGCACSAGAGCTATCAGATGGGCTGAACTCAAGAGGAAAACAAAGGCGGAAAAGAAACTAGCAATCACTGTATTTAGCTTCCCACCAGACAAAGGCAATGTAGGAACTGCTGCTTACCTCAATGTCTTCTCTTCCATCTACTCTGTGCTCAAAGAGCTCAAGAAAGATGGCTACAATGTTGATGGCCTTCCAGAAACTTCAGAAGCCTTGATCGAAGAAATCATCCACGATAAGGAGGCCCAATTCAACAGCCCCAATCTCAACGTAGTTCACAAGATGAGCGTCCGGGAATACCAGAGTCTGACTCCTTACTCTACTGCTCTTGAAGAAAACTGGGGGAAGCCTCCAGGCAACTTGAACTCCGATGGTGAAAATCTTCTCGTCTATGGCAAACAGTATGGAAACATCTTCATTGGTGTTCAGCCTACTTTTGGCTACGAGGGTGATCCTATGCGTCTTCTCTTCTCCAAATCCGCCAGCCCACACCACGGATTTGCTGCATACTACTCATATGTGGAGAAGATCTTTAAGGCCGACGCAGTTCTGCACTTTGGAACTCATGGTTCTCTTGAATTCATGCCTGGGAAACAAGTTGGCATGAGTGATGCCTGCTTCCCCGACAGTCTTATTGGAAACATCCCCAACATCTACTACTACGCCGCTAACAACCCGTCTGAGGCCACTGTTGCAAAGAGGCGTAGCTATGCGAATACTATCAGTTACCTGACTCCTCCAGCTGAGAACGCAGGCCTCTACAAAGGGCTGAAGCAGTTGAGCGAGCTGATTGCTTCTTACCAATCTCTCAAAGATTCAGGCCGTGGCCCTCAAATTGTGAGCTCTATTATCAGTACTGCTAGGCAGTGCAATCTTGATAAAGATGTGGTTCTTCCTGAAGACGGAGTCGAGCTCTCTCAAAAAGAGCGTGACTCTGTAGTGGGACAAGTRTATTCCAAGATTATGGAGATCGAATCGAGATTGCTACCTTGTGGCCTTCATGTCATTGGTGAGCCTCCAACAGCAATGGAGGCAGTGGCTACACTGGTCAACATCGCTGCATTGGATCGTGAAGAGGAACAGATTTCGTCTCTTCCTTCTATATTAGCTCAGACTGTGGGGAGAGAGATTGAAGATATCTACAGAGGAAGTGATAAGGGTGTTCTGCGCGATGTGGAGCTCCTTAAACAGATCACCGAAGTATCTCGAGGTGCAATCAGTGCTTTTGTGGAAAGAAGCACCAACAGCAAAGGGCAAGTTGTTGAAGTAGCTGATAAGCTCACATCAATCCTTGGTTTTGGTGTAAACGAGCCATGGATTCAATACTTGTCGAACACCAAGTTTTACCGGGCTGACAGAGAAAAACTCAGAGTCTTGTTCGGATACTTAGGTGAATGCTTGAGACTGATTGTGGCTGATAATGAGGTGGGAAGCTTGAAACAAGCTTTGGAAGGGAAGTACGTCGAGCCAGGGCCAGGTGGGGACCCGATCAGAAACCCGAAGGTGCTGCCTACAGGAAAGAACATCCATGCCTTGGACCCGCAGTCCATTCCAACAACTGCAGCTATGCAGAGTGCTAAAGTGGTGGTGGAGAGGCTACTCGAGAGGCAGAAGATCGATAATGGTGGAAAATATCCCGAGACAGTTGCTTTAGTACTATGGGGTACTGATAACATCAAGACATATGGTGAATCACTAGCTCAAGTTCTTTGGATGATTGGAGTTACACCGGTAACTGATGGGCTTGGGCGTGTTAACCGTGTGGAGATCGTGAGCCTTGAAGAGCTCGGAAGGCCGAGAATTGATGTCGTTGTCAACTGCTCCGGGGTCTTCAGAGATCTCTTCATCAATCAGATGAATCTTCTTGATAGGGCAGTGAAGATGGTAGCTGAGCTGGACGAGCCGGAGGAGCAGAACTACGTGAGGAAACATGCATTGGAGCAAGCCAAAGAGCTGGGAGTTGAAGTCAGAGAAGCTGCCTCACGTATTTTCTCAAACGCCTCCGGCTCATACTCCTCCAACATCAATCTTGCTGTTGAGAACTCATCTTGGAATGATGAGAAGCAGCTACAGGACATGTACTTGAGCAGAAAGTCRTTTGCATTCGACTCTGATGCCCCAGGTGTAGGCATGACGGAGAAACGAAAGATCTTTGAGATGGCTCTCAGCACAGCCGATGCCACCTTCCAGAATCTTGATTCCTCAGAGATATCCCTCACCGATGTCAGTCACTACTTCGATTCAGACCCAACTAATCTCGTACAAGGCCTCAGGAAGGACGGGAAGAAGCCGAGTGCATACATTGCAGACACCACCACAGCTAATGCACAGGTGCGGACGCTRTCTGAGACAGTGAGGCTCGATGCAAGGACCAAGCTGTTGAATCCCAAGTGGTATGAGGGCATGCTGTCCAGTGGCTATGAGGGTGTTCGTGAGATTGAGAAACGTCTCACAAACACTGTGGGTTGGAGCGCAACTTCAGGACAAGTCGACAACTGGGTGTACGAGGAGGCCAACACAACATTCATCCAAGACGAGGAGATGCTGAACAGGCTGATGAGCACGAACCCGAACTCGTTCAGGAAATTGCTTCAGACTTTCTTGGAGGCTAATGGAAGAGGATACTGGGAGACTAGTGAAGAAAACATTGAGAGGTTAAAGCAGTTGTACTCTGAAGTTGAAGACAAGATTGAAGGAATAGATCGTTAG

>NcG8H

ATGGATTTCCTCACAATCTTCATTGGACCATTACTTGCCATCATCGCCTTATTCTATGCACGCTTAACAATCTCCACGAGGGGCAAAAACCTGCCCCCGGGGCCGACGCCGCTTCCCTTCATCGGAAACCTCCATTTGCTCGGCCACCACCCCCACAAGTCCCTCGCCCGTCTGGCCAAGACTTACGGCCCACTCATGCTCCTCCGCCTCGGCTCCGAAAACACCGTCGTTGTCTCCTCCGCCGCCGTCGCTAAAGAAGTCCTCCAGAAGCATGACCTCGCCTTCTCCAGCAGGAGCATCCCCGACGCCGTCCATGCTCACGACCAGTTCAAGTACTCCGTCGTTTGGCTCCCGGTGGCCTCGCGGTGGCGCAGCCTCCGCAAAACCATGAACTCCAACATCTTCTCCGGCAGCTGCCTCGACGCCAATCAGCATCTTAGGAGCAGGAAGGTGGAGGAGCTCATTGCCTACTGCAAGGAAAGCTGCCGGAGGGCGGAAGCCGTCGACGTCGGCCGCGCCGCCTTCAGAACCGCCTTGAACTCGCTCTCCAACACCATATTTTCGAGGGATTTGGCCGATCCGTTGTCGGATTCCGGTAAGGAATTCAAGGATGTGGTGTCGAGTATCATGATGGAGGCAGGGAAGCCCAACTCCGTGGATTACTATCCTTTTTTATCAAGATTTGATCCACAAGGAATACGACGTCGCACCGCTATCTATTTCGACAAATTGATCAATATTTTCAAAGGTTTGATCGATGAAAGGTTGGAGAAAAGAAGATCTCAATACGAAGAAGCAACTGATGTTATCGATATCCTCCTCAACACCATCGAGCAAAACCCCGAAGAGATCGACCGAACTCACATCGAACGAATGTTTTTGGATCTGTTCTTGGCGGGAACGGACACAACTTCGAGCACAGTGGAATGGGCAATGGCGGAGGCGATGAAAAATCCAGAAACAATGAAGAAAGCCAAGGCAGAGCTAGCACAAGTGATCGGAAAAGGAAAAATAGTACAAGAAACCGACATACCGCATCTGCCATATCTGCAATGCATTGTTAAAGAGACGCTGAGACTACACCCGCCGGTTCCCTTCCTCATTCCACGCAGAGTCCACGAAGACGTCGACATCTTGGGCTACGTCGTGCCCAATAACTCGCAGGTCTTCGTGAACATGTGGGCCATCGGGCGGGACCCCGATATGTGGAAAAACCCTCTACAGTTCGACCCCGACCGCTTCCACGATTCGGAGGTGGGGTTGAGGGGGAAAGATTTCGAGCTGATTCCATTCGGCGCGGGCAGAAGAATCTGCCCGGGGCTGCCGCTGGCGATGAGAATGGTGCCCGTAGTGCTGGGGTCGCTTCTGAATTCATTCGACTGGAAAGTGGAAGGAGGGAAGGAATTAGAGATGAATGAGAAGTTTGGAATCACGTTGGAGAAGGCTCAACCACTTATGGCTGTCCCTATCGCTCTT

>NcGES

ATGTCGTGTGCAGGGAGCACCATCTTATCTTTGTCACAATCATCATTTCCCGATCGCTTCCCAAGGACCTCAATTTCATGGCATACAAGATTTTGCCCCGCCTCCGTCTCCCTTCGTCGCTTCTCCGTCTGCAAGCTGCAGGCGGCAACCACGACATCACCCAATTTGGCCCCCGTAGTCCACAACAACGATCATATGCTTGAAATTGTGGAGAGCACAAAGAAGAGAGAATATTTGGTTGAGAAGATTACAGAAAAGCTCACAGAAGCATCATCAGAGAAACTCAAACTCATCGATGAAATCCAACGGCTGGGAATCGGCTACCACTTTGAAGATGTCATCAACAGCATACTGCAGGTCCAGTGCTCCGCTTTCTCTACTGAAGAAGACCTCTTCACCACTGCTCTGCGCTTCCGTCTGCTCCGCCAGGCTGGCTTCCACGTCACCCCCGAGGTGTTTACGAGAGTCAAGGACAAAAGTGGAAATTTCAAAGAATCCTTAGGCGGGGACACACTTGGGTTATTGAGTTTATATGAAGCGTCGCATATAGGAGTTGATGGAGAGAGAATGTTGGAGGATGCTAGACAATTTACAGAGTCTTATTTGAGACAATCACTAGCTAAGCTGGCACCTCGCCTTTGTAGCGAGGTGGGCCAGGCCCTAGAGGTCCCGAGGCATCATAGGATGGTTAGATTGGAAGCAAGAAGATTCGTTCAGGAGTATGGTAAGCAAAGTGGCCATGATCGAGACCTTTTGGAGCTAGCAATATTGGATTATAACCAAGTTCAAGCTCAACACAACATGGAACTCACTGAAATTGTAAGATGGTGGAAAGAGCTAGGTTTGGTGGATAAGTTAAGTTTTGCACGAGATAGACCGTTGGAGTGTTTTTTATGGTCGGTTGGACTTCTTCCCGACCCGAAGTACTCAAATTGCAGAATAGAGCTTGCCAAAAATATAGCTATCTTATTAGTGATCGATGATATTTTCGACACTTATGGCAAGATGGATGATCTAATCCTCTTCACCCATGCAATTCGAAGATGGGATCTTGAAGCAATGGAAACCTTACCCGAGTACATGAAAATATGCTATATGGCATTGTACAATACAACGAACGAGACATGCTATCGAGTGCTTAAGGAAACTGGACGAACTGTCTTCCCATATTTCAAATCCACGTGGATAGACATGATCGAAGGATTCATGGTGGAGGCGAAGTGGTTCAATGGTGGAATTGAACCTAATTTGGAAGAATATATAGAGAATGGAGTGTCTACTGCGGGTGCATACATGGCTTTGGTGTACCTCTTCTTTCTAATTGGAGAAGGTGTAACCGACCAAAACGCCTCACTTTTGATTAGAAAACCCTATCCTAAGCTCTTCTCTGCCGCCGGTCGAATTCTTCGCCTTTGGGATGATCTCGGAACTGCTAAGGAGGAAGAAGAGCGTGGTGATCTGGCGTCAAGCACCCACTTATTCATGAAAGAGGAGAATTTATCAACAGAGGAAGATGCTAGAAGTTGCATTTTGGACGAAATTTTCCGATTGTGGAAAGATTTGAATGGGGAGCTCATATCCGATAATAAAGTGTTGCCATTGTCCATAATCAAAGTCGCACTTGACATGGCACGATCTTCCCAAGTTGTGTACAAGCACGAAGGCGACACTTATTTTTCAAGCGTGGACAATTATGTCGAAGCCCTATTTTTCACTCCTCTTGTTTCATCT
