# Supplementary material for: Integrating cotyledon-based virus-induced gene silencing with visual marker promises a rapid, highly effective validation of gene functions in Nepeta cataria
Source: Front Plant Sci. 2025 Jan 21;15:1514614. doi: 10.3389/fpls.2024.1514614 (PMC11790630; doi:10.3389/fpls.2024.1514614)
Supplement: Supplementary Data Sheet 3 — The protein sequences of ChlHs used for phylogenetic analysis. [file DataSheet3.docx]

>NP_001268078.1 VvChlH [Vitis vinifera]

MASLVSSPFTLPTSKVDQLSSFSQKHYFLHSFLPKKTNQANSKSCLRVKCAAIGSGLFTQTTPEVRRIVP

DNDHGLPTVKVVYVVLEAQYQSALTAAVQTLNSKARYASFQVVGYLVEELRDEATYKTFCKGLEDANIFI

GSLIFVEELALKVKAAVEKERDRLDAVLVFPSMPEVMRLNKLGSFSMSQLGQSKSPFFQLFKKKKSSAGF

ADSMLKLVRTLPKVLKYLPSDKAQDARLYILSLQFWLGGSPDNLMNFLKMISGSYVPALKRTKIEYSDPV

LFLDSGIWHPLAPCMYDDVKEYLNWYGTRRDANEKLKGPNAPVIGLVLQRSHIVTGDESHYVAVIMELEA

RGAKVIPIFAGGLDFSGPVERFLIDPVTKRPFVNSVVSLTGFALVGGPARQDHPRAVEALMKLDVPYIVA

LPLVFQTTEEWLNSTLGLHPIQVALQVALPELDGGMEPIVFAGRDPRTGKSHALHKRVEQLCIRAIRWAE

LKRKSKAEKKLAITVFSFPPDKGNVGTAAYLNVFDSIFSVLKELKRDGYNVEGLPETSESLIEDVLHDKE

AKFSSPNLNIAYKMGVREYQTLTPYATALEESWGKPPGNLNSDGENLLVYGKQYGNVFIGVQPTFGYEGD

PMRLLFSKSASPHHGFAAYYSFVEKIFKADAVLHFGTHGSLEFMPGKQVGMSDVCYPDSLIGNIPNVYYY

AANNPSEATIAKRRSYANTISYLTPPAENAGLYKGLKQLSELISSYQSLKDTGRGPQIVSSIISTAKQCN

LDKDVSLPDEGEEISAKERDLVVGKVYSKIMEIESRLLPCGLHVIGEPPSAMEAVATLVNIAALNRPEEG

ISSLPAILAETVGRNIEDVYRGSDKGILKDVELLRQITDTSRGAISAFVERTTNKKGQVVDVADKLTSVF

GFGLNEPWVQYLSSTKFYQADREKLRTLFAFLGECLKLVVADNELRSLKQALEGKYVEPGPGGDPIRNPK

VLPTGKNIHALDPQSIPTAAALQSAMVVVDRLLERQKADNGGKYPETVALVLWGTDNIKTYGESLAQVLW

MIGVRPVADTFGRVNRVEPVSLEELGRPRIDVVVNCSGVFRDLFINQMNLLDRAVKMVAELDEPADQNYV

RKHALEQAQALGIEVRDAATRVFSNASGSYSSNINLAVENSSWNDEKQLQDMYLSRKSLAFDCDAPGAGM

TEKRKVFEMALSTADATFQNLDSSEISLTDVSHYFDSDPTNLVQGLRKDGKKPNAYIADTTTANAQVRTL

SETVRLDARTKLLNPKWYEGMMSSGYEGVREIEKRLTNTVGWSATSGQVDNWVYEEANSTFIQDEEMLKR

LMNTNPNSFRKLVQTFLEANGRGYWETSEDNIEKLRQLYSEVEDKIEGIDR

>AXB26707.1 GmChlH [Glycine max]

MASLVSSPFTLPNSKVDQLSSLAQRHLFLHSFLPKKANGYASSSKASLRVKCAAMGNGLFTQTTPEVRRI

VPEKNQGLPTVKIVYVVLEAQYQSSLSAAVRVLNSNKKDASFEVVGYLVEELRDESTYKTFCKDLEDANI

FIGSLIFVEELALKVKAVVEKERDRLDAVLVFPSMPEVMRLNKLGSFSMSQLGQSKSPFFQLFKKKKQSS

AGFADSMLKLVRTLPKVLKYLPSDKAQDARLYILSLQFWLGGSPDNLQNFLKMISGSYVPALKGTKMEYS

EPVLYLDSGIWHPLAPCMYDDVKEYLNWYGTRRDANEKLKSPNAPVIGLILQRSHIVTGDDGHYVAVIME

LEARGAKVIPIFAGGLDFSGPVERYLIDPITKKPFVNSVVSLTGFALVGGPARQDHPRAVEALMKLDVPY

IVALPLVFQTTEEWLNSTLGLHPIQVALQVALPELDGGMEPIVFAGRDPKTGKSHALHKRVEQLCTRAIK

WAELKRKTKEEKKLAITVFSFPPDKGNVGTAAYLNVFSSIFSVLKDLQRDGYNVEGLPETSEALIEEVIH

DKEAQFSSPNLNVAYKMNVREYQSLTPYATALEENWGKPPGNLNSDGENLLVYGKQYGNVFIGVQPTFGY

EGDPMRLLFSKSASPHHGFAAYYSFVEKIFKADAVLHFGTHGSLEFMPGKQVGMSDVCYPDSLIGNIPNI

YYYAANNPSEATIAKRRSYANTISYLTPPAENAGLYKGLKQLSELISSYQSLKDTGRGPQIVSSIISTAR

QCNLDKDVELPEEGEEIPAKDRDLVVGKVYAKIMEIESRLLPCGLHVIGEPPSALEAVATLVNIAALDRP

EDGISSLPSILAETVGRSIEEVYRGSDKGILKDVELLRQITEASRGAITSFVQRTTNKKGQVVDVADKLT

SILGFGINEPWVEYLSNTKFYRADREKLRTLFDFLGECLKLVVADNELGSLKQALEGKYVEPGPGGDPIR

NPKVLPTGKNIHALDPQAIPTTAAMQSAKIVVDRLIERQKAENGGKYPETVALVLWGTDNIKTYGESLAQ

VLWMIGVNPVADTFGRVNRVEPVSLEELGRPRIDVVVNCSGVFRDLFINQMNLLDRAVKMVAELDEPAEQ

NFVRKHALEQAQALGIDVREAATRVFSNASGSYSSNINLAVENSSWNDEKQLQDMYLSRKSFAFDCDAPG

AGMTEKRKVFEMALSTADATFQNLDSSEISLTDVSHYFDSDPTNLVQSLRKDGKKPSAYVADTTTANAQV

RTLAETVRLDARTKLLNPKWYEGMLSTGYEGVREIEKRLTNTVGWSATSGQVDNWVYEEANTTFIQDEEM

LKKLMNTNPNSFRKLVQTFLEANGRGYWETSEDNIDKLRQLYSEVEDKIEGIDR

>ACO57443.1 PpChlH [Prunus persica]

MASLVSSPFTLPHTKADQLSSLSRKQYFLHSFLPKKVNQSSLKSSLKVKCAMGSYGLFTQTTQEVRRIVP

ENKQGLPTVKIVYVVLEAQYQSSLTAAVQALNSNSKYASFEVVGYLVEELRDAETYKMFCQDLEDANIFI

GSLIFVEELAVKVRDAVEKERDRLDAVLVFPSMPEVMRLNKLGSFSMSQLGQSKSPFFQLFKRKKPESAG

FADSMLKLVRTLPKVLKYLPSDKAQDARLYILSLQFWLGGSPDNLQNFLKMISGSYVPALKGEKIPYSDP

VLFLDSGIWHPLAPCMYDDVKEYLNWYGTRKDANEKLKSPNAPVVGLILQRSHIVTGDESHYVAVIMELE

ARRAKVIPIFAGGLDFSGPVERFLIDPVTKKPFIHSAISLTGFALVGGPARQDHPRAVEALMKLDVPYIV

ALPLVFQTTEEWLNSTLGLHPIQVALQVALPELDGGMEPIVFAGRDPRTGKSHALHKRVEQLCTRAIRWG

ELKRKAKAEKKLAITVFSFPPDKGNVGTAAYLNVFSSIFAVLQELKRDGYNVENLPETSEALIEDVIHDK

EAQFSSPNLNVAYKMGVREYQSLTPYATALEENWGKPPGNLNSDGENLLVYGKQYGNVFIGVQPTFGYEG

DPMRLLFSKSASPHHGFAAYYSFVEKIFQADAVLHFGTHGSLEFMPGKQVGMSDACFPDSLIGNIPNVYY

YAANNPSEATIAKRRSYANTISYLTPPAENAGLYKGLKQLSELISSYQSLKDTGRGSQIVSSIISTAKQC

NLDKDVELPEEGLEISAKERDLVVGKVYNKIMEIESRLLPCGLHVIGEPPTAMEAVATLVNIAALNRPEE

GITSLPDILAETAGRGIEDLYRGSDKGILKDVELLKQITDTSRGAISAFVERTTNEKGQVVDVKDKLSSI

LGFGINEPWVQYLSNTKFYRADRDKLRTLFMFLGECLKLIVADNEIGSLKQALEGKYVEPGPGGDPIRNP

EVLPTGKNIHALDPQSIPTTAAMQSAKIVVERLIERQKIDNGGKYPETIALVLWGTDNIKTYGESLAQVL

WMVGVMPVADAFGRVNRVEIVSLEELGRPRIDVVVNCSGVFRDLFINQMNLLDRAVKMVAELDEPVEQNF

IRKHALEQAETLGIGVREAATRIFSNASGSYSSNINLAVENSSWNDEKQLQDMYLSRKSFAFDSDAPGVG

MAENRKVFEMALSTAEATFQNLDSSEISLTDVSHYFDSDPTNLVQSLRKDGKKPSAYIADTTTANAQVRT

LSETVRLDARTKLLNPKWYEGMLSSGHRVVREIEKRLTNTVGWSATSGQVDNWVYEEANTTFIQDKEMLE

RLMKTNPNSFRKLVQTFLEANGRGYWDTAEENIEKLKELYQEVEDKIEGIDR

>AEN74910.1 FahlH [Fragaria x ananassa]

MASLVSSPFTLPQTKPDQLSSFSKKHYFLHSFLPRKTNQASSKTTLKVKCAMGNGLFTQTTQEVRRIVPE

NKQNLPTVKVVYVVLEAQYQSSLTAAVQSLNASNKHASFSVVGYLVEELRDDDTYKTFCQDLQDANVFIG

SLIFVEELALKVKQAVEKERDRMDAVLVFPSMPEVMRLNKLGSFSMSQLGQSKSPFFQLFKRKKQGAGFA

DSMLKLVRTLPKVLKYLPSDKAQDARLYILSLQFWLGGSPDNLQNFLKMIAGSYIPALKGEKIPYSDPVL

FLDSGIWHPLAPCMYDDVKEYLNWYGTRKDANEKLKSPSAPIVGLILQRSHIVTGDESHYVAVIMELEAR

GAKVIPIFAGGLDFSGPVERFLIDPVTKKPFIHSAISLTGFALVGGPARQDHPRAIEALMKLDVPYIVAL

PLVFQTTEEWLNSTLGLHPIQVALQVALPELDGGMEPIVFAGRDPRTGKSHALHKRVEQLCTRAIRWGEL

KRKAKAEKKLAITVFSFPPDKGNVGTAAYLNVFSSIFSVLQELKRDGYYVEGLPETSDALIEEVIHDKEA

QFSSPNLNIAYKMGVREYQSLTPYAAALEENWGKPPGNLNSDGENLLVYGKQYGNVFIGVQPTFGYEGDP

MRLLFSKSASPHHGFAAYYSFVEKIFQADAVLHFGTHGSLEFMPGKQVGMSDACFPDSLIGNIPNVYYYA

ANNPSEATIAKRRSYANTISYLTPPAENAGLYKGLKQLSELIASYQSLKDTGRGQQIVSSIISTARQCNL

DKDVDLPDEGVEISAKERDLVVGKVYNKIMEIESRLLPCGLHVIGEPPTAMEAVATLVNIAALNRPEENI

FSLPAILAETVGRDIEDLYRQSDKGILKDVELLKQITDASRGAVSSFVECTTNEKGQVVDVKNKLTSILG

FGINEPWIQYLSNTKFYRADREKLRTLFEYLGECLKLIVADNEIGSLKQALEGKFVEPGPGGDPIRNPKV

LPTGKNIHALDPQSIPTTAAMNSAKVVVERLIERQKLDNGGKYPETIALVLWGTDNIKTYGESLAQVLWM

VGVNPVADGLGRVNKVEVVPLEELGRPRIDVVVNCSGVFRDLFINQMNLLDRAVKMVAELDEPLEQNFVR

KHALEQAETLGIGVREAATRIFSNASGSYSSNINLAVENSSWNDEKQLQDMYLSRKSFAFDCDAPGAGMA

ENRKVFEMALSTADATFQNLDSSEISLTDVSHYFDSDPTNLVQNLRKDGKKPSSYIADTTTANAQVRTLS

ETVRLDARTKLLNPKWYEGMLSSGYEGVREIEKRLTNTVGWSATSGQVDNFVYEEANATFIKDEEMLNRL

MKTNPNSFRKLLQTFLEANGRGYWDTDEENIERLKELYSEVEDKIEGIDR

>AST11845.1 protoporphyrin IX magnesium chelatase subunit H [Ocimum basilicum]

MASLVSSPFTLPNSKAVNLSSLSQKHYLLHSFLPKNPNRANTHSSQKFKCAAIGNGLFTQTSPEVRRIVP

EKSNNLSTVKIVYVVLEAQYQSSLSAAVQQLNSNGEFASFELVGYLVEELRDESTYKTFCKDLEDANIFI

GSLIFVEELALKVKAAVEKERERLDAVLVFPSMPEVMRLNKLGSFSMSQLGQSKSPFFQLFKKKNGKSSA

GFADSMLKLVRTLPKVLKYLPSDKAQDARLYILSLQFWLGGSPDNLVNFLKMISSSYVPALKGAKVEYSD

PVLYLDSGIWHPLAPCMYDDVKEYLNWYATRRDANEQLKSPNAPVIGLVLQRSHIVTGDESHYVAVIMEL

EARGAKVIPIFAGGLDFSGPVERYFIDPITKQPMINSVVSLTGFALVGGPARQDHPRAVEALMKLDVPYI

VALPLVFQTTEEWLNSTLGLHPIQVALQVALPELDGGMEPIVFSGRDPRTGKSHALHKRVEQLCTRAIKW

AELKRKTKAEKRLAITVFSFPPDKGNVGTAAYLNVFSSIYSVLKDLKRDGYNVEGLPETAEALIEDVIHD

KEAQFNSPNLNIVYKMGVREYQKLTPYSTALEENWGKPPGNLNSDGENLLVYGKQYGNVFIGVQPTFGYE

GDPMRLLFSKSASPHHGFAAYYSYVEKIFKADAVLHFGTHGSLEFMPGKQVGMSDACYPDSLIGNIPNIY

YYAANNPSEATVAKRRSYANTISYLTPPAENAGLYKGLKQLGELISSYQSLKDTGRGPQIVSSIISAAKQ

CNLDKDVDLPDEGAEISAKERDLVVGKVYSKIMEIESRLLPCGLHVIGEPPSAMEAVATLVNIAALDRPE

DEISSLPSILAQTVGREMEDVYRGSDKGILRDVELLRQITEASRGAISAFVERSTNEKGQVVDVSNKLTS

ILGFGINEPWIQYLQNTKFYRADREKLRVLFQFLGECLKLVVADNELGSLKQALEGKYVEPGPGGDPIRN

PKVLPTGKNIHALDPQAIPTTAAMQSAKVVVDRLLERQKIENGGKYPETVALVLWGTDNIKTYGESLAQV

LWMIGVRPVADTFGRVNRVEPVSLEELGRPRVDVVVNCSGVFRDLFINQMNLLDRAVKMVAELDEPEEQN

YVRKHALEQAKELGVEVREAASRIYSNASGSYSSNINLAVENSSWNDEKQLQDMYSSEISLTDVSHYFDS

DPTNLVQTLRKDGKKPSAYIADTTTANAQVRTLSETVRLDARTKLLNPKCLSRKSFAFDCDAPGAGMTEK

RKIFEMALSTADATFQNLDSSEISLTDVSHYFDSDPTNLVQTLRKDGKKPSAYIADTTTANAQVRTLSET

VRLDARTKLLNPKWYEGMLSSGYEGVREIEKRLTNTVGWSATSGQVDNWVYEEANTTFIQDEQMLNRLMS

TNPNSFRKLIQTFLEANGRGYWETSAENIEKLRQLYSEVEDKISTV

>NcChlH1

MASLVSSPFTLPKSRIEHLSSLSQKHHLLHSFLPKNPTTKSSKKFQCAAIGNGLFTQTTQEVRRIVPDKSNGLPAVKIVYVVLEAQYQSSLTAAVQTLNSNGKYASFEVVGYLVEELRDANTYKTFCQDLEDANIFIGSLIFVEELALKVKSAVEKERERLDAVLVFPSMPEVMRLNKLGSFSMSQLGQSKSPFFQLFKKKNKSSAGFADSMLKLVRTLPKVLKYLPSDKAQDARMYILSLQFWLGGSPDNLVNFLKMISGSYIPALKGTKIEYADPVLYLDSGIWHPLAPCMYDDVKEYLNWYDTRRDTNDQLKSKNAPVVGLVLQRSHIVTGDESHYVAVIMELEARGAKVIPIFAGGLDFSGPVERYFINPITKKPMVNSVVSLTGFALVGGPARQDHPRAVEALMKLDVPYIVALPLVFQTTEEWLNSTLGLHPIQVALQVALPELDGGMEPIVFSGRDPRTGKSHALHKRVEQLCTRAIKWGELKRKTKAEKKLAITVFSFPPDKGNVGTAAYLNVFASIYSVLKDLKNDGYNVEGLPETAEGLIEDVIHDKEAQFNSPNLNVAYKMGVREYQSLTPYASALEENWGKPPGNLNSDGENLLVYGKQYGNVFIGVQPTFGYEGDPMRLLFSKSASPHHGFAAYYSYVEKIFKADAVLHFGTHGSLEFMPGKQVGMSDACFPDSLIGNIPNVYYYAANNPSEATIAKRRSYANTISYLTPPAENAGLYKGLKQLSELISSYQSLKDTGRGPQIVSSIISTARQCNLDKDVDLPEEGVEISAKERDLVVGQVYSKIMEIESRLLPCGLHVIGEPPSAMEAVATLVNIAALDRPEEGISSLPSILAQTVGREIEDVYRGSDKGILKDVELLCQITDASRGAITAFVEKTTNKKGQVVDVTDKLTSILGFGVNEPWVDHLSNTKFYRADREKLRVLFQFLGECLKLVVADNELGSLKQALEGKYVEPGPGGDPIRNPKVLPTGKNIHALDPQAIPTTAALQSAKVVVDRLLERQKMDNGGKYPETVALVLWGTDNIKTYGESLAQVMWMIGVRPVADTFGRVNKVEAVSLEELGRPRIDVVVNCSGVFRDLFINQMNLLDRAVKMVAELDEPEDQNYVRKHALEQAKELGVEVRDAASRIFSNASGSYSSNVNLAVENSSWNDEKQLQDMYLSRKSFAFDSDAPGAGMTEKRKIFEMALSTAEATFQNLDSSEISLTDVSHYFDSDPTNLVQNLRKDGKKPNAYIADTTTANAQVRTLSETVRLDARTKLLNPKWYEGMLSSGYEGVREIEKRLTNTVGWSATSGQVDNWVYEEANTTFIQDEQMLNRLMNSNPNSFRKLVQTFLEANGRGYWETSAENIERLRQLYSEVEDKIEGIDR

>NcChlH2

MASLVSSPFTLPKSRIEHLSSLSQKHHLLHSFLPKKPTTKSSKKFQCTAIGNGLFTQTTQEVRRIVPDKSSGLPAVKIVYVVLEAQYQSSLTAAVQTLNSNGKYASFEVVGYLVEELRDANTYKTFCQDLEDANIFIGSLIFVEELALKVKSAVEKERERLDAVLVFPSMPEVMRLNKLGSFSMSQLGQSKSPFFQLFKKKNKSSAGFADSMLKLVRTLPKVLKYLPSDKAQDARMYILSLQFWLGGSPDNLVNFLKMISGSYIPALKGTKIEYADPVLYLDSGIWHPLAPCMYDDVKEYLNWYDTRRDTNDQLKSKNAPVVGLVLQRSHIVTGDESHYVAVIMELEARGAKVIPIFAGGLDFSGPVERYFINPITKKPMVNSVVSLTGFALVGGPARQDHPRAVEALMKIDVPYIVALPLVFQTTEEWLNSTLGLHPIQVALQVALPELDGGMEPIVFSGRDPRTGKSHALHKRVEQLCTRAIKWGELKRKTKAEKKLAITVFSFPPDKGNVGTAAYLNVFASIYSVLKDLKNDGYNVEGLPETAEGLIEDVIHDKEAQFNSPNLNVAYKMGVREYQSLTPYASALEENWGKPPGNLNSDGENLLVYGKQYGNVFIGVQPTFGYEGDPMRLLFSKSASPHHGFAAYYSYVEKIFKADAVLHFGTHGSLEFMPGKQVGMSDACFPDSLIGNIPNVYYYAANNPSEATIAKRRSYANTISYLTPPAENAGLYKGLKQLSELISSYQSLKDTGRGPQIVSSIISTARQCNLDKDVDLPEEGVEISAKERDLVVGQVYSKIMEIESRLLPCGLHVIGEPPSAMEAVATLVNIAALDRPEEGISSLPSILVQTVGREIEDVYRGSDKGILKDVELLRQITDASRGAITAFVEKTTNKKGQVVDVADKLTSILGFGVNEPWVDHLSNTKFYRADREKLRVLFQFLGECLKLVVADNELGSLKQALEGKYVEPGPGGDPIRNPKVLPTGKNIHALDPQAIPTTAALQSAKVVVDRLLERQKMDNGGKYPETVALVLWGTDNIKTYGESLAQVMWMIGVRPVADTFGRVNKVEAVSLEELGRPRIDVVVNCSGVFRDLFINQMNLLDRAVKMVAELDEPEDQNYVRKHALEQAKELGVEVRDAASRIFSNASGSYSSNVNLAVENSSWNDEKQLQDMYLSRKSFAFDSDAPGAGMTEKRKIFEMALSTAEATFQNLDSSEISLTDVSHYFDSDPTNLVQNLRKDGKKPNAYIADTTTANAQVRTLSETVRLDARTKLLNPKWYEGMLSSGYEGVREIEKRLTNTVGWSATSGQVDNWVYEEANTTFIQDEQMLNRLMNSNPNSFRKLVQTFLEANGRGYWETSAENIERLRQLYSEVEDKIEGIDR

>NmChlH

MASLVSSPFTLPKSRIEHLSSLSQKHHLLHSFLPKKPTTKSSKKFQCAAIGNGLFTQTTQEVRRIVPDKSNGLPVVKIVYVVLEAQYQSSLTAAVQTLNSNGKYASFEVVGYLVEELRDANTYKTFCQDLEDANIFIGSLIFVEELALKVKSAVEKERERLDAVLVFPSMPEVMRLNKLGSFSMSQLGQSKSPFFQLFKKKNKSSAGFADSMLKLVRTLPKVLKYLPSDKAQDARMYILSLQFWLGGSPDNLVNFLKMISGSYIPALKGTKIEYADPVLYLDSGIWHPLAPCMYDDVKEYLNWYDTRRDTNDQLKSKNAPVVGLVLQRSHIVTGDESHYVAVIMELEARGAKVIPIFAGGLDFSGPVERYFINPITKKPMVNSVVSLTGFALVGGPARQDHPRAVEALMKLDVPYIVALPLVFQTTEEWLNSTLGLHPIQVALQVALPELDGGMEPIVFSGRDPRTGKSHALHKRVEQLCTRAIKWGELKRKTKAEKKLAITVFSFPPDKGNVGTAAYLNVFASIYSVLKDLKNDGYNVEGLPETAEGLIEDVIHDKEAQFNSPNLNVAYKMGVREYQSLTPYSSALEENWGKPPGNLNSDGENLLVYGKQYGNVFIGVQPTFGYEGDPMRLLFSKSASPHHGFAAYYSYVEKIFKADAVLHFGTHGSLEFMPGKQVGMSDACFPDSLIGNIPNVYYYAANNPSEATIAKRRSYANTISYLTPPAENAGLYKGLKQLSELISSYQSLKDSGRGPQIVSSIISTARQCNLDKDVDLPEEGIEISAKERDLVVGQVYSKIMEIESRLLPCGLHVIGEPPSAMEAVATLVNIAALDRPEEGISSLPSILAQTVGREIEDVYRGSDKGILKDVELLRQITDASRGAITAFVEKTTNKKGQVVDVTDKLTSILGFGVNEPWVDHLSNTKFYRADREKLRVLFQFLGECLKLVVADNELGSLKQALEGKYVEPGPGGDPIRNPKVLPTGKNIHALDPQAIPTTAALQSAKVVVDRLLERQKMDNGGKYPETVALVLWGTDNIKTYGESLAQVMWMIGVRPVADTFGRVNKVEAVSLEELGRPRIDVVVNCSGVFRDLFINQMNLLDRAVKMVAELDEPEDQNYVRKHALEQAKELGVEVRDAASRIFSNASGSYSSNVNLAVENSSWNDEKQLQDMYLSRKSFVFDSDAPGAGMTEKRKIFEMALSTAEATFQNLDSSEISLTDVSHYFDSDPTNLVQNLRKDGKKPNAYIADTTTANAQVRTLSETVRLDARTKLLNPKWYEGMLSSGYEGVREIEKRLTNTVGWSATSGQVDNWVYEEANTTFIQDEQMLNRLMNSNPNSFRKLVQTFLEANGRGYWETSAENIERLRQLYSEVEDKIEGIDR

>Q9FNB0

MASLVYSPFTLSTSKAEHLSSLTNSTKHSFLRKKHRSTKPAKSFFKVKSAVSGNGLFTQT

NPEVRRIVPIKRDNVPTVKIVYVVLEAQYQSSLSEAVQSLNKTSRFASYEVVGYLVEELR

DKNTYNNFCEDLKDANIFIGSLIFVEELAIKVKDAVEKERDRMDAVLVFPSMPEVMRLNK

LGSFSMSQLGQSKSPFFQLFKRKKQGSAGFADSMLKLVRTLPKVLKYLPSDKAQDARLYI

LSLQFWLGGSPDNLQNFVKMISGSYVPALKGVKIEYSDPVLFLDTGIWHPLAPTMYDDVK

EYWNWYDTRRDTNDSLKRKDATVVGLVLQRSHIVTGDDSHYVAVIMELEARGAKVVPIFA

GGLDFSGPVEKYFVDPVSKQPIVNSAVSLTGFALVGGPARQDHPRAIEALKKLDVPYLVA

VPLVFQTTEEWLNSTLGLHPIQVALQVALPELDGAMEPIVFAGRDPRTGKSHALHKRVEQ

LCIRAIRWGELKRKTKAEKKLAITVFSFPPDKGNVGTAAYLNVFASIFSVLRDLKRDGYN

VEGLPENAETLIEEIIHDKEAQFSSPNLNVAYKMGVREYQDLTPYANALEENWGKPPGNL

NSDGENLLVYGKAYGNVFIGVQPTFGYEGDPMRLLFSKSASPHHGFAAYYSYVEKIFKAD

AVLHFGTHGSLEFMPGKQVGMSDACFPDSLIGNIPNVYYYAANNPSEATIAKRRSYANTI

SYLTPPAENAGLYKGLKQLSELISSYQSLKDTGRGPQIVSSIISTAKQCNLDKDVDLPDE

GLELSPKDRDSVVGKVYSKIMEIESRLLPCGLHVIGEPPSAMEAVATLVNIAALDRPEDE

ISALPSILAECVGREIEDVYRGSDKGILSDVELLKEITDASRGAVSAFVEKTTNSKGQVV

DVSDKLTSLLGFGINEPWVEYLSNTKFYRANRDKLRTVFGFLGECLKLVVMDNELGSLMQ

ALEGKYVEPGPGGDPIRNPKVLPTGKNIHALDPQAIPTTAAMASAKIVVERLVERQKLEN

EGKYPETIALVLWGTDNIKTYGESLGQVLWMIGVRPIADTFGRVNRVEPVSLEELGRPRI

DVVVNCSGVFRDLFINQMNLLDRAIKMVAELDEPVEQNFVRKHALEQAEALGIDIREAAT

RVFSNASGSYSANISLAVENSSWNDEKQLQDMYLSRKSFAFDSDAPGAGMAEKKQVFEMA

LSTAEVTFQNLDSSEISLTDVSHYFDSDPTNLVQSLRKDKKKPSSYIADTTTANAQVRTL

SETVRLDARTKLLNPKWYEGMMSSGYEGVREIEKRLSNTVGWSATSGQVDNWVYEEANST

FIQDEEMLNRLMNTNPNSFRKMLQTFLEANGRGYWDTSAENIEKLKELYSQVEDKIEGID

R

>Q10M50

MSSLVSTPFTTATGVQKKLGAPVPLHSFLLSRRQPAAGAGRGRAAAAAIRCAVAGNGLFT

QTKPEVRRVVPPEGDASRRGVPRVKVVYVVLEAQYQSSVTAAVRELNADPRRAAGFEVVG

YLVEELRDEETYKTFCADLADANVFIGSLIFVEELALKVKDAVEKERDRMDAVLVFPSMP

EVMRLNKLGSFSMSQLGQSKSPFFQLFKRKKNSGGFADSMLKLVRTLPKVLKYLPSDKAQ

DARLYILSLQFWLGGSPDNLQNFLKMIAVSYVPALKGADIKYDDPVLFLDAGIWHPLAPT

MYDDVKEYLNWYGTRRDTNDKLKDPNAPVIGLVLQRSHIVTGDDGHYVAVIMELEAKGAK

VIPIFAGGLDFSGPTQRYLVDPITGKPFVNAVVSLTGFALVGGPARQDHPKAIAALQKLD

VPYIVALPLVFQTTEEWLNSTLGLHPIQVALQVALPELDGGMEPIVFAGRDPRTGKSHAL

HKRVEQLCTRAIRWAELKRKTKEEKKLAITVFSFPPDKGNVGTAAYLNVFNSIYSVLQDL

KKDGYNVEGLPDTAEALIEEVIHDKEAQFNSPNLNVAYRMNVREYQSLTSYASLLEENWG

KPPGNLNSDGENLLVYGKQYGNVFIGVQPTFGYEGDPMRLLFSKSASPHHGFAAYYTFVE

KIFQADAVLHFGTHGSLEFMPGKQVGMSDACYPDSLIGNIPNIYYYAANNPSEATVAKRR

SYANTISYLTPPAENAGLYKGLKQLSELISSYQSLKDTGRGPQIVSSIISTAKQCNLDKD

VPLPEEGVELPPNERDLIVGKVYAKIMEIESRLLPCGLHVIGEPPSAIEAVATLVNIASL

DRPEDEIYSLPNILAQTVGRNIEDVYRGSDKGILADVELLRQITEASRGAITTFVERTTN

NKGQVVDVTNKLSTMLGFGLSEPWVQHLSKTKFIRADREKLRTLFTFLGECLKLIVADNE

LGSLKLALEGSYVEPGPGGDPIRNPKVLPTGKNIHALDPQAIPTTAALKSAKIIVDRLLE

RQKVDNGGKYPETIALVLWGTDNIKTYGESLAQVLWMIGVRPVADTFGRVNRVEPVSLEE

LGRPRIDVVINCSGVFRDLFINQMNLLDRAVKMVAELDEPEEMNYVRKHAQEQARELGVS

LREAATRVFSNASGSYSSNVNLAVENASWTDEKQLQDMYLSRKSFAFDCDAPGAGMREQR

KTFELALATADATFQNLDSSEISLTDVSHYFDSDPTKLVQGLRKDGRAPSSYIADTTTAN

AQVRTLSETVRLDARTKLLNPKWYEGMMKSGYEGVREIEKRLTNTVGWSATSGQVDNWVY

EEANATFIEDEAMRKRLMDTNPNSFRKLVQTFLEASGRGYWETSEENLEKLRELYSEVED

KIEGIDR

>B8ANF1

MSSLVSTPFTTATGVQKKLGAPVPLHSFLLSRRQPAAGAGRGRAAAAAIRCAVAGNGLFT

QTKPEVRRVVPPEGDASRRGVPRVKVVYVVLEAQYQSSVTAAVRELNADPRRAAGFEVVG

YLVEELRDEETYKTFCADLADANVFIGSLIFVEELALKVKDAVEKERDRMDAVLVFPSMP

EVMRLNKLGSFSMSQLGQSKSPFFQLFKRKKNSGGFADSMLKLVRTLPKVLKYLPSDKAQ

DARLYILSLQFWLGGSPDNLQNFLKMIAVSYVPALKGADIKYDDPVLFLDAGIWHPLAPT

MYDDVKEYLNWYGTRRDTNDKLKDPNAPVIGLVLQRSHIVTGDDGHYVAVIMELEAKGAK

VIPIFAGGLDFSGPTQRYLVDPITGKPFVNAVVSLTGFALVGGPARQDHPKAIAALQKLD

VPYIVALPLVFQTTEEWLNSTLGLHPIQVALQVALPELDGGMEPIVFAGRDPRTGKSHAL

HKRVEQLCTRAIRWAELKRKTKEEKKLAITVFSFPPDKGNVGTAAYLNVFNSIYSVLQDL

KKDGYNVEGLPDTAEALIEEVIHDKEAQFNSPNLNVAYRMNVREYQSLTSYASLLEENWG

KPPGNLNSDGENLLVYGKQYGNVFIGVQPTFGYEGDPMRLLFSKSASPHHGFAAYYTFVE

KIFQADAVLHFGTHGSLEFMPGKQVGMSDACYPDSLIGNIPNIYYYAANNPSEATVAKRR

SYANTISYLTPPAENAGLYKGLKQLSELISSYQSLKDTGRGPQIVSSIISTAKQCNLDKD

VPLPEEGVELPPNERDLIVGKVYAKIMEIESRLLPCGLHVIGEPPSAIEAVATLVNIASL

DRPEDEIYSLPNILAQTVGRNIEDVYRGSDKGILADVELLRQITEASRGAITAFVERTTN

NKGQVVDVTNKLSTMLGFGLSEPWVQHLSKTKFIRADREKLRTLFTFLGECLKLIVADNE

LGSLKLALEGSYVEPGPGGDPIRNPKVLPTGKNIHALDPQAIPTTAALKSAKIVVDRLLE

RQKVDNGGKYPETIALVLWGTDNIKTYGESLAQVLWMIGVRPVADTFGRVNRVEPVSLEE

LGRPRIDVVVNCSGVFRDLFINQMNLLDRAVKMVAELDEPEEMNYVRKHAQEQARELGVS

LREAATRVFSNASGSYSSNVNLAVENASWTDEKQLQDMYLSRKSFAFDCDAPGAGMREQR

KTFELALATADATFQNLDSSEISLTDVSHYFDSDPTKLVQGLRKDGRAPSSYIADTTTAN

AQVRTLSETVRLDARTKLLNPKWYEGMMKSGYEGVREIEKRLTNTVGWSATSGQVDNWVY

EEANATFIEDEAMRKRLMDTNPNSFRKLVQTFLEASGRGYWETSEENLEKLRELYSEVED

KIEGIDR

>A0A0D3B0Z5

MASSLMYSPFTLSASRAEHLSSLSNTTTKHSFLRRKSKPTKPATSIFKVKSSVSGNGLFT

QTNPEVRRIVPVKRDNVPTVKIVYVVLEAQYQSSLSEAVQQLNKTSRFASYEVVGYLVEE

LRDKNTYKSFCKDLEDANIFIGSLIFVEELALKVKDAVEKERDRMDAVLVFPSMPEVMRL

NKLGSFSMSQLGQSKSPFFQLFKRKKGAGSAGFADSMLKLVRTLPKVLKYLPSDKAQDAR

LYILSLQFWLGGSPDNLQNFVKMISGSYIPALKGVKIEYSDPVLFLDTGIWHPLAPTMYD

DVKEYLNWYDTRRDTNASLKRKDATVIGLVLQRSHIVTGDDSHYVAVIMELEARGAKVIP

IFAGGLDFSGPVERYFVDPVTKQPIINSAVSLTGFALVGGPARQDHPRAIEALKTLDVPY

LVGVPLVFQTTEEWLNSTLGLHPIQVALQVALPELDGGMEPIVFAGRDPRTGKSHALHKR

VEQLCIRAIRWGELKRKTKTEKRVAITVFSFPPDKGNVGTAAYLNVFASIYSVLKDLKRD

GYNVEGLPETAETLIEEILHDKEAQFSSPNLNVAYKMGVREYQSLTPYAAALEENWGKPP

GNLNSDGENLLVFGKTYGNVFIGVQPTFGYEGDPMRLLFSKSASPHHGFAAYYSYVEKIF

KADAVLHFGTHGSLEFMPGKQVGMSDACFPDSLIGNIPNVYYYAANNPSEATIAKRRSYA

NTISYLTPPAENAGLYKGLKQLSELISSYQSLKDTGRGPQIVSSIISTAKQCNLDKDVDL

PEEGTDLSVKERDLVVGKVYSKIMEIESRLLPCGLHVIGEPPSAMEAVATLVNIAALDRA

EEEISSLPSILAECVGRQIEDVYRGSDKGILSDVELLKQITDASRGAVSAFVEKTTNSKG

QVVNVSDKLTSILGFGINEPWVEYLSNTKFYRANRDKLRTVFTFLGECLKLVVMDNELGS

LMQALEGKYVEPGPGGDPIRNPKVLPTGKNIHALDPQAIPTTAAMASAKIVVDRLVERQK

LENEGKYPETIALVLWGTDNIKTYGESLGQVLWMIGARPVADGLGRVNRVEPVSLEELGR

PRIDVVVNCSGVFRDLFINQMNLLDRAIKMVAELDEPVEMNYVRKHAMEQAATLGVDIRE

AATRVFSNASGSYSSNISLAVENSSWNDEKQLQDMYLSRKSFAFDSDAPGAGMAEKKQVF

EMALMTAEVTFQNLDSSEISLTDVSHYFDSDPTNLVQSLRKDKKKPSAYIADTTTANAQV

RSLSETVRLDARTKLLNPKWYEGMMSSGYEGVREIEKRLTNTVGWSATSGQVDNWVYEEA

NTTFIKDEEMLNRLMNTNPNSFRKMIQTFLEANGRGYWETSEDNIEKLKDLYSQVEDKIE

GIDR

>A0A1S2Z2H7

MASFVSTPFTLPNSKPDQLSSLAQRQLFLHSFLPKKTNYHNTSKASFRLKCNAIGNGLFT

QTTQEVRRIVPENKQNLPTVKIVYVVLEAQYQSSVSAAVRALNSNQNDASFEVVGYLVEE

LRDVSTYQTFCKDLEDANIFIGSLIFVEELALKVKSAVEKERERLDAVLVFPSMPEVMRL

NKLGSFSMSQLGQSKSPFFQLFKKKKTSSAGFADSMLKLVRTLPKVLKYLPSDKAQDARL

YILSLQFWLGGSPDNLQNFLKMISGSYVPALKGTKMEYSEPVLFLDNGIWHPLAPCMYDD

VKEYLNWYGTRRDANEKLKSPNAPVVGLILQRSHIVTGDEGHYVAVIMELEAKGAKVIPI

FAGGLDFSGPVEKFLIDPITKKPFVNSVISLTGFALVGGPARQDHPRAVEALMKLDVPYI

VALPLVFQTTEEWLNSTLGLHPIQVALQVALPELDGGMEPIVFSGRDPKTGKSHALHKRV

EQLCTRAIKWAELKRKTKEEKKLAITVFSFPPDKGNVGTAAYLNVFSSIFSVLKELERDG

YNVDGLPETSEALIEDILHDKEAQFSSPNLNIAYKMSVREYQNITPYSTALEENWGKPPG

NLNADGENLLVYGKQYGNVFIGVQPTFGYEGDPMRLLFSKSASPHHGFAAYYSYVEKIFK

ADAVLHFGTHGSLEFMPGKQVGMSDVCYPDSLIGNIPNVYYYAANNPSEATIAKRRSYAN

TISYLTPPAENAGLYKGLKQLSELISSYQSLKDTGRGQQIVSSIISTAKQCNLDKDVDLP

EEGVELPTKERDLVVGKVYAKIMEIESRLLPCGLHVIGEPPSAMEAVATLVNIAALDRAE

EDISSLPSILAQSVGRNIEEIYRASDKGILKDVELLRQITEASRGAITSFVERTTNNKGQ

VVDVSNKLTSILGFGINEPWIQYLSNTKFYRGDREKLRTLFDFLGECLRLIVADNEVGSL

KQALEGKYVEPGPGGDPIRNPKVLPTGKNIHALDPQSIPTTAAMQSAKIVVERLLERQKA

DNGGKFPETVALVLWGTDNIKTYGESLAQVLWMIGVNPISDTFGRVNRVEPVSVEELGRP

RIDVVVNCSGVFRDLFINQMNLLDRAVKMVAELDEPAEQNFVRKHAIEQAEALGVEVREA

ATRIFSNASGSYSSNINLAVENSSWNDEKQLQDMYLSRKSFAFDCDAPGAGMTEKRKVFE

MALSTADATFQNLDSSEISLTDVSHYFDSDPTNLVQNLRKDGKKPSAYVADTTTANAQVR

TLSETVRLDARTKLLNPKWYEGMLSSGYEGVREIEKRLTNTVGWSATSGQVDNWVYEEAN

TTFIQDEEMLKKLMNTNPNSFRKLVQTFLEANGRGYWETEEENIEKLRQLYSEVEDKIEG

IDR

>A0A1U7YMV7

MASLVSSPFTSPASKVEHLSSLSQKHFFLHSFLPKKLNQGCSSSRAGMRVKCAAIGNGLF

TQTTPEVRRILPDQNPDLPRVKIVYVVLEAQYQSSLSAAVRSLNSSGKYASFEVVGYLVE

ELRDKSTYQTFCKDIEDANIFIGSLIFVEELARKIKAAVEKERDRLDAVLVFPSMPEVMR

LNKLGSFSMSQLGQSKSPFFQLFKKKKSSAGFADNMLKLVRTLPKVLKYLPSDKAQDARL

YILSLQFWLGGSPDNLQNFIKMISGSYVPALKGTKVEYADPVVFLDSGMWHPLAPCMYDD

VKEYLNWYGTRKDANEKLKDPNAPVVGLILQRSHIVTGDESHYVAVIMELEARGAKVIPI

FACGLDFSGPVEKFLIDPVTKKPFVHSVVSLTGFALVGGPARQDHPRAVEALRKLDVPYI

VALPLVFQTTEEWLNSTLGLHPIQVALQVALPELDGGMEPIVFSGRDPRTGKSHALHKRV

EQLCTRSINWAELKRKSKVEKRLAITVFSFPPDKGNVGTAAYLNVFASIYSVLKELQRDG

YNVEGLPETAEALIEDIIHDKEAKFSSPNLNIAYKMGVREYQSLTPYVTALEESWGKPPG

NLNSDGENLLVYGKQYGNVFIGVQPTFGYEGDPMRLLFSKSASPHHGFAAYYSFVEKIFK

ADAVLHFGTHGSLEFMPGKQVGMSDVCYPDSLIGNIPNVYYYAANNPSEATIAKRRSYAN

TISYLTPPAENAGLYKGLKQLSELISSYQSLKDTGRGPQIVSSIISTAKQCNLDKDVKLP

EEGEELSAKERDLVIGKVYSKIMEIESRLLPCGLHVIGEPPSAMEAVATLVNIAALDRPE

DGISSLPAVLAETVGRDIEGVYRGSDKGILKDVELLHQITETSRGAISAFVERTTNKKGQ

VVDVANKLSSILGFGLNEPWAQYLSNTKFYRADREKLRTLFEFLGECLKLFVADNELGSL

KQALEGSYVEPGPGGDPIRNPKVLPTGKNIHALDPQSIPTVAAMQSAKVVVDRLLERQKS

DNGGQYPETVALVLWGTDNIKTYGESLAQVFWMIGVRPVSDTLGRVNRVEPVSLEELGRP

RIDVVVNCSGVFRDLFINQMNLLDSAVKMVAELDEPDDQNYVKKHATQQAEALGIGLREA

ATRVFSNASGSYSSNINLAVENSSWNDEKQLQDMYLSRKSFAFDCDAPGAGMTEKRKVFE

MALSTADATFQNLDSSEISLTDVSHYFDSDPTNLVQSLRKDGKKPSAYIADTTTANAQVR

TLADTVRLDARTKLLNPKWYEGMMASGYEGVREIEKRLTTTVGWSATSGQVDNWVYDEAN

STFIQDEEMLNRLMNTNPNSFRKLVQTFLEANGRGYWETSEQNIERLRQLYSEVEDKIEG

IDR

>A0A1U7ZFD6

MASLVSSPFTLPASKVDHLSSLSQKHFLLHSFLPRKLNQYSSSRTGLRVKCAATGNGLFT

QTTPEVRRILPDQKPGLPRVKIVYVVLEAQYQSSLSAAVRSLNSKGRYASFGVVGYLVEE

LRDESTYQTFCKDLEDANIFIGSLIFVEELARKVKAAVEKERDRLDAVLVFPSMPEVMRL

NKLGSFSMSQLGQSKSPFFQLFKKKKSSAGFADSMLKLVRTLPKVLKYLPSDKAQDARLY

ILSLQFWLGGSPDNLQNFIKMISGSYVPALKGAKIEYADPVLFLDTGIWHPLAPCMYDDV

KEYLNWYGTRKDANEKLKDPNAPVIGLVLQRSHIVTGDDGHYVAVIMELEARGAKVIPIF

AGGLDFSGPVEKFFIDPISKKPFVHSAVSLTGFALVGGPARQDHPRAVEALTKLDVPYIV

ALPLVFQTTEEWLNSTLGLHPIQVALQVALPELDGGMEPIVFSGRDPRTGKSHALHKRVE

QLCTRAIKWAELKRKTKTEKRLAITVFSFPPDKGNVGSAAYLNVFASIYSVLKDLQKDGY

NVEGLPETAEALIEDVIHDKEAQFSSPNLNIAYKMGVREYQSLTPYATALEESWGKPPGN

LNSDGENLLVYGKQYGNVFIGVQPTFGYEGDPMRLLFSKSASPHHGFAAYYTFVEKVFKA

DAVLHFGTHGSLEFMPGKQVGMSDVCYPDSLIGNIPNVYYYAANNPSEATIAKRRSYANT

ISYLTPPAENAGLYKGLKQLSELISSFQSLKDTGRGPQIVSSIISTAKQCNLDKDVNLPE

EGEELSAKERDLVVGKVYSKIMEIESRLLPCGLHVIGEPPSAMEAVATLVNIAALDRPEE

GISSLPAILAETVGRDIEDVYRGNDKGILKDVELLRQITEASRGAISAFVERTTNNKGQV

VDVANKLSSILGFGLNEPWVQYLSNTKFYRADREKLRTLFQFLGECLKLVVADNELGSLK

QALEGSYVEPGPGGDPIRNPKVLPTGKNIHALDPQAIPTAAAMQSAKVVVERLLERQKAD

NGGEYPETVALVLWGTDNIKTYGESLAQVLWMIGVRPVADTFGRVNRVEPVSLEELGRPR

IDVIVNCSGVFRDLFINQMNLLDSAVKMVAELDEPEDQNYVKKHAVQQAQALSIGLREAA

TRVFSNASGSYSSNINLAVENSSWNDEKQLQDMYLSRKSFAFDCDAPGAGMTEKRKVFEM

ALSTADATFQNLDSSEISLTDVSHYFDSDPTNLVQNLRKDGKKPSAYIADTTTANAQVRT

LAETVRLDARTKLLNPKWYEGMMASGYEGVREIEKRLTNTVGWSATSGQVDNWVYEEANS

TFIQDEEMLNRLMNTNPNSFRKLVQTFLEANGRGYWETSEQNIERLRQLYSEVEDKIEGI

DR

>A0A1U8HTJ8

MASLVSSPFTLPASKADQLSSLSQKHFFLHSFLPKKINNLPNSKSSFKVKCAAIGNGLFT

QTTPEVRRIVPENKNNLPTVKIVYVVLEAQYQSSLSSAVQSLNQNSNFASFEVVGYLVEE

LRDENTYKTFCKDLEGANIFIGSLIFVEELALKVKTAVEKERDRLDAVLVFPSMPEVMRL

NKLGSFSMSQLGQSKSPFFQLFKRKKQGAGFADSMLKLVRTLPKVLKYLPSDKAQDARLY

ILSLQFWLGGSPDNLQNFLKMISSSYVPALKGTKVDYSDPVLFLDSGIWHPLAPCMYDDV

KEYLNWYGTRRDVNEKLRGPDAPVIGLVLQRSHIVTGDESHYVAVIMELEAKGAKVIPIF

AGGLDFSGPVERFLIDPVTKKPMVNSVVSLTGFALVGGPARQDHPRAVEALMKLDVPYIV

ALPLVFQTTEEWLNSTLGLHPIQVALQVALPELDGGMEPIVFAGRDPRTGKSHALHKRVE

QLCTRAIKWAELKRKSKTEKKLAITVFSFPPDKGNVGTAAYLNVFASIYSVLKDLQKDGY

NVEGLPETAEALIEDVIHDKEAQFNSPNLNVAYKMSIREYQNLTPYAPALEENWGKPPGN

LNSDGENLLVYGKQYGNVFIGVQPTFGYEGDPMRLLFSKSASPHHGFAAYYSFVEKIFEA

DAVLHFGTHGSLEFMPGKQVGMSDVCYPDSLIGNIPNVYYYAANNPSEATIAKRRSYANT

ISYLTPPAENAGLYRGLKQLSELISSYQSLKDSGRGQQIVSSIISTARQCNLDKDVELPE

EGEEISAKERDLVVGKVYSKIMEIESRLLPCGLHVIGEPPSAMEAVATLVNIAALDRPED

GISSLPSILAETVGRNIEDVYRGSDKGILKDVELLRQITEASRGAISAFVQKTTNKNGQV

VDVADKLSSILGFGINEPWIQYLSNTKFYRADREKLRVLFEFLGECLKLVVADNELGSLK

QALEGKYVEPGPGGDPIRNPKVLPTGKNIHALDPQAIPTTAAMQSAKIVVDRLVERQKVD

NGGKYPETVALVLWGTDNIKTYGESLAQVLWMIGVRPVADTFGRVNRVEPVSLEELGRPR

IDVVVNCSGVFRDLFINQMNLLDRAVKMVAELDEPVEQNYVRKHALEQAKALGIEVREAA

TRVFSNASGSYSSNVNLAVENSSWNDEKQLQDMYLSRKSFAFDCDAPGAGMTEKRKVFEM

ALSTADATFQNLDSSEISLTDVSHYFDSDPTNLVQNLRKDGKKPSAYIADTTTANAQVRT

LAETVRLDARTKLLNPKWYEGMMSSGYEGVREIEKRLTNTVGWSATSGQVDNWVYEEANS

TFIQDENMLSRLMNTNPNSFRKLIQTFLEANGRGYWETSEENIEKLRQLYSEVEDKIEGI

DR

>A0A223FRD8

MASLVSSPFTLPNSKAVNLSSLSQKHYLLHSFLPKNPNRANTHSSQKFKCAAIGNGLFTQ

TSPEVRRIVPEKSNNLSTVKIVYVVLEAQYQSSLSAAVQQLNSNGEFASFELVGYLVEEL

RDESTYKTFCKDLEDANIFIGSLIFVEELALKVKAAVEKERERLDAVLVFPSMPEVMRLN

KLGSFSMSQLGQSKSPFFQLFKKKNGKSSAGFADSMLKLVRTLPKVLKYLPSDKAQDARL

YILSLQFWLGGSPDNLVNFLKMISSSYVPALKGAKVEYSDPVLYLDSGIWHPLAPCMYDD

VKEYLNWYATRRDANEQLKSPNAPVIGLVLQRSHIVTGDESHYVAVIMELEARGAKVIPI

FAGGLDFSGPVERYFIDPITKQPMINSVVSLTGFALVGGPARQDHPRAVEALMKLDVPYI

VALPLVFQTTEEWLNSTLGLHPIQVALQVALPELDGGMEPIVFSGRDPRTGKSHALHKRV

EQLCTRAIKWAELKRKTKAEKRLAITVFSFPPDKGNVGTAAYLNVFSSIYSVLKDLKRDG

YNVEGLPETAEALIEDVIHDKEAQFNSPNLNIVYKMGVREYQKLTPYSTALEENWGKPPG

NLNSDGENLLVYGKQYGNVFIGVQPTFGYEGDPMRLLFSKSASPHHGFAAYYSYVEKIFK

ADAVLHFGTHGSLEFMPGKQVGMSDACYPDSLIGNIPNIYYYAANNPSEATVAKRRSYAN

TISYLTPPAENAGLYKGLKQLGELISSYQSLKDTGRGPQIVSSIISAAKQCNLDKDVDLP

DEGAEISAKERDLVVGKVYSKIMEIESRLLPCGLHVIGEPPSAMEAVATLVNIAALDRPE

DEISSLPSILAQTVGREMEDVYRGSDKGILRDVELLRQITEASRGAISAFVERSTNEKGQ

VVDVSNKLTSILGFGINEPWIQYLQNTKFYRADREKLRVLFQFLGECLKLVVADNELGSL

KQALEGKYVEPGPGGDPIRNPKVLPTGKNIHALDPQAIPTTAAMQSAKVVVDRLLERQKI

ENGGKYPETVALVLWGTDNIKTYGESLAQVLWMIGVRPVADTFGRVNRVEPVSLEELGRP

RVDVVVNCSGVFRDLFINQMNLLDRAVKMVAELDEPEEQNYVRKHALEQAKELGVEVREA

ASRIYSNASGSYSSNINLAVENSSWNDEKQLQDMYSSEISLTDVSHYFDSDPTNLVQTLR

KDGKKPSAYIADTTTANAQVRTLSETVRLDARTKLLNPKCLSRKSFAFDCDAPGAGMTEK

RKIFEMALSTADATFQNLDSSEISLTDVSHYFDSDPTNLVQTLRKDGKKPSAYIADTTTA

NAQVRTLSETVRLDARTKLLNPKWYEGMLSSGYEGVREIEKRLTNTVGWSATSGQVDNWV

YEEANTTFIQDEQMLNRLMSTNPNSFRKLIQTFLEANGRGYWETSAENIEKLRQLYSEVE

DKISTV

>A0A3Q7G0W1

MASLVSSPFTLPNSKVEHLSSISQKHYFLHSFLPKKTNPTFSKSPKKFQCNAIGNGLFTQ

TTQEVRRIVPENLKGLATVKIVYVVLEAQYQSALTAAVQTLNKNGEFASFEVVGYLVEEL

RDENAYKTFCKDLEDANIFIGSLIFVEELALKVKSAVEKERDRLDAVLVFPSMPEVMRLN

KLGSFSMSQLGQSKSPFFQLFKKKKSSAGFSDQMLKLVRTLPKVLKYLPSDKAQDARLYI

LSLQFWLGGSPDNLVNFLKMVSGSYVPALKGVKMDYSDPVLYLDSGIWHPLAPCMYDDVK

EYLNWYATRRDTNEKLKSSSAPVIGLVLQRSHIVTGDESHYVAVIMELEARGAKVIPIFA

GGLDFSGPVERYFIDPITKKPFVNSVVSLTGFALVGGPARQDHPRAIEALTKLDVPYIVA

LPLVFQTTEEWLNSTLGLHPIQVALQVALPELDGGMEPIVFSGRDPRTGKSHALHKRVEQ

LCTRAIKWGELKRKSKAEKKLAITVFSFPPDKGNVGTAAYLNVFASIYSVLKDLKKDGYN

VEGLPETSAELIEEVIHDKEAQFSSPNLNVAYKMNVREYQKLTPYATALEENWGKAPGNL

NSDGENLLVYGKQYGNVFIGVQPTFGYEGDPMRLLFSKSASPHHGFAAYYSFVEKIFKAD

AVLHFGTHGSLEFMPGKQVGMSDACFPDSLIGNIPNVYYYAANNPSEATIAKRRSYANTI

SYLTPPAENAGLYKGLKQLSELIASYQSLKDSGRGPQIVSSIISTARQCNLDKDVDLPDE

EKEIDAKERDLVVGKVYAKIMEIESRLLPCGLHIIGEPPTAMEAVATLVNIAALDRAEDD

ISSLPSILAATVGRNIEEIYRGNDNGVLRDVELLRQITEASRGAISAFVERSTNNKGQVV

DNSDKLTSLLGFSINEPWIQYLSNTQFYRADREKLRVLFQFLGECLKLIVANNEVGSLKQ

ALEGKYVEPGPGGDPIRNPKVLPTGKNIHALDPQAIPTTAALQSAKIVVERLLERQKIDN

GGKYPETVALVLWGTDNIKTYGESLAQVMWMIGVRPVADTLGRVNRVEPVSLEELGRPRV

DVVVNCSGVFRDLFINQMNLLDRGIKMVAELDEPEDQNFVRKHALEQAKTLGIDVREAAT

RVFSNASGSYSSNINLAVENSSWNDEKQLQDMYLSRKSFAFDCDAPGAGMMEKRKVFEMA

LSTADATFQNLDSSEISLTDVSHYFDSDPTNLVQNLRKDGKKPSAYIADTTTANAQVRTL

SETVRLDARTKLLNPKWYEGMLSTGYEGVREIEKRLTNTVGWSATSGQVDNWVYEEANTT

FIKDEEMLNRLMNTNPNSFRKLLQTFLEANGRGYWDTSEENIEKLKQLYSEVEDKIEGID

R

>A0A4P9CZC7

MSSLMSSSSFTLPTSKTEHLSSLSHKHSIFNSSSSKKIRPRKIGFGVRCAAVGNGLFTQS

TPEVRRVVPNGNQGLPSVKIVYVVLEAQYQSSLTTAVKTLNREGRYAAFEVVGYLVEELR

DENTYKAFCEDLKDANIFIGSLIFVEELAQKVKVAVETERDRLDAVLVFPSMPEVMRLNK

LGSFSMSQLGQSKSPFFQLFKRKKQSAGFAESMLKLVRTLPKVLKYLPSDKAQDARLYIL

SLQFWLGGSPENLLNFVKMISGSYIPALKGMKIAYSDPVLFLDSGIWHPLAPCMYDDVKE

YLNWYGTRKDANEKLKDPNSPIIGLILQRSHIVTGDDSHYVAVIMELEARGAKVIPIFAG

GLDFSGPTERYLVDPVTKKPFVHSAISLTGFALVGGPARQDHPRAIEALRKLDVPYIVAL

PLVFQTTEEWLISSLGLHPIQVALQVALPELDGGMEPIVFSGRDGRTGKSHALHKRVEQL

CTRAIRWAELKRKSKEEKKVAITVFSFPPDKGNVGTAAYLNVFASIFSVVKDLQRDGYNV

DGLPETSEALIEDIIHDKEAKFSSPNLNVAYKMNVREYQSLTPYAAALEESWGKPPGNLN

SDGEHLLVYGKQYGNVFIGVQPTFGYEGDPMRLLFSKSASPHHGFAAYYSFVEKIFKADA

VLHFGTHGSLEFMPGKQVGMSDVCYPDSLIGNIPNIYYYAANNPSEATIAKRRSYANTIS

YLTPPAENAGLYKGLKQLSELISSYQSLKDSGRGPQIVSSIISTAKQCNLDKDVSLPEEG

EELSSKQRDLVVGKVYSKIMEIESRLLPCGLHVIGEPPSAMEAVATLVNIAALDRPEDGI

ISLPGILAATVGRNIEDVYRGNDNGILKDVELLRQITEASRGSISAFVERTTNKKGQVVD

VAGKLGSILGLGINEPWIQYLANTKFYQADRDKLRTLFEFLGECLKLVVADNELGSLKQA

LEGSYVEPGPGGDPIRNPKVLPTGKNIHALDPQAIPTEAAMQSAKVVVERLLERQKADNG

GQYPETVALVLWGTDNIKTYGESLGQVLWMIGVRPIADTFGRVNRVEPVSLEELGRPRID

VVVNCSGVFRDLFINQMNLLDRAVKMVAELDEPEDQNFVKKHALEQAEALGVSLREAATR

VFSNASGSYSSNVNLAVENSSWNDEKQLQDMYLSRKSFAFDSDAPGVGMSEKRKVFELAL

STAEATFQNLDSSEISLTDVSHYFDSDPTNLVQTLRKDGKKPSAYIADTTTANAQVRTLS

ETVRLDARTKLLNPKWYEGMIKSGYEGVREIEKRLTNTVGWSATSGQVDNWVYEEANTTF

IQDEDMLKRLMDTNPNSFRKLVQTFLEANGRGYWETSEDNIEKLRQLYSEVEDKIEGIDR

>A0A4V6MB88

MQRLAGRTASVARTAVPSVPRSSAQSARKVACNVQTGPRPKLTTFTNNKSVSIDMRSEDG

DSGLFTSTDPESRRVIPEVNGRVRVKVVYVVLEAQYQAALTTAVKRINDTNSKVCFEIVG

YLLEELRDAENYEAFKADVANTNIFIGSLIFIEELADKIVSAIEPLRSSMDACLVFPSMP

AVMKLNKLGTFNMSQLGGGKSIIGDFIKSARKNNDNFEEGLLKLVRTLPKVLKFLPSDKA

QDARNFVNSLQYWLGGNQENLENLLLNVSQEYVPALKGAGMATSEPVLPPDTGIWHPCAP

QMFEDLKEYLNWYETRKDMTFAADAPVIGLVLQRSHLVTGDSGHYDGVVSEMEAKGAKVI

PVFAGGLDFSSPVKKFFYDPLGSGRSYVDTVVSLTGFALVGGPARQDAPKAVAALTELNV

PYLCSLPLVFQTTEEWMDSELGVHPVQVALQVALPELDGAIEPIVFAGRDSATGKSHSLP

DRVTSLCRRAINWARLRKLKNKEKKLAVTVFSFPPDKGNVGTAAYLDVFGSIFKVLKNLQ

NEGYDVGNLPLNSQMLLKSVTNDTDAKINSVDLNIAYRMPVKEYEELCEYSGSLEENWGK

PPGQLNSSGNDMLVYGKQFGNIFIGVQPTFGYEGDPMRLLFSRSASPHHGFAAYYTFLEK

IFKADAVLHFGTHGSLEFMPGKQVGMSGVCYPDSLIGSIPNIYYYAANNPSEATIAKRRS

YANTISYLTPPAENAGLYKGLKELKELIASYQGMRESSRAIQIVATICETARGCNLDRDV

ALPADDEAMKALNMDERDSIVGKVYKELMQIESRLLPCGLHIVGCAPTAMEAIATLVNIG

ELDRPDNQPSPVLGMPGILARSIGRKIEDIYAGNNRGELFEVDTLQRITACSRELVREFV

LDRTGVDGRIGVPVLTTISKWVGLYEDPFSRVLRGSEFAACDRLQLAETFKYLEYCLTQI

VNDNELGALVEGLNGEYVKPGPGGDPIRNPLVLPTGKNIHALDPQSIPTVAAIKSAAVVV

ERLLDRERSLNGGKYPETIALVLWGTDNIKTYGESLAQVMMMVGIRPVPDALGRVNKLEV

IPLEELKRPRVDVVVNCSGVFRDLFVNQMLLLDRAVKMAAELDEPDELNFVRKHAKEQAK

ELGLKSIRDAAVRIFSNSSGSYSSNVNLAVENSSWTDEAQLQEMYLKRKSFAFNSDRPGA

GGEEQRAVFEASMKTVDATFQNLDSSEISLTDVSHYFDSDPTKLVQSLRTDGKAPSAYIA

DTTTANAQVRTLGETVRLDARTKLLNPKWYEGMLGSGYEGVREIQKRLTNTMGWSATSGM

VDNWVYDEANSTFIEDPTMAARLMEANPNSFRKLVATFLEANGRGYWDASPEQLEKLKQL

YMDVEDKIEGVE

>A0A6I9S7M2

MSSLVSTPFAPPNSTRTEHLSSVSQKHIFLHSFLPRKPSHSARNGFRVRCTAIGNGLFTQ

TKPEVRRILPDASPGLPKVKVVYVVLEAQYQSSVSAAVRSLNADRRHASFEVVGYLVEEL

RDESTYQTFRKDLEDANIFIGSLIFVEELAQKVKVAVEKERDRMDAVLVFPSMPEVMRLN

KLGSFSMSQLGQSKSPFFQLFKRKKQSAGFAESMLKLVRTLPKVLKYLPSDKAQDARLYI

LSLQFWLGGSPENLQNFLKMITGSYVPALKATKIDYADPVLFLDSGIWHPLAPCMYDDAK

EYLNWYGTRRDANEKLKDPNSPVIGLVLQRSHIVTGDDGHYVAVIMELEARGAKVIPIFS

GGLDFSGPAERFLVDPISNKPFVHAVVSLTGFALVGGPARQDHPRAIEALRKLDVPYIVA

LPLVFQTTEEWLISSLGLHPIQVALQVALPELDGGMEPIVFSGRDARTGKSHALHKRVEQ

LCTRAIRWAELKRKSKEEKKVAITVFSFPPDKGNVGTAAYLNVFSSIFSVLRGLKKDGYD

VDGLPDTPEALIEDVIHDKEAKFSSPNLNVAYRMSVREYQALTPYASALEENWGKPPGNL

NSDGEHLLVYGKQYGNVFIGVQPTFGYEGDPMRLLFSKSASPHHGFAAYYSFVEKIFEAD

AVLHFGTHGSLEFMPGKQVGMSDVCYPDSLIGNIPNIYYYAANNPSEATIAKRRSYANTI

SYLTPPAENAGLYKGLKQLAELISSYQSLKDTGRGVQIVSSIISTAKQCNLDKDVSLPEE

GVELSAKERDLVVGKVYSKIMEIESRLLPCGLHVIGEPPSAMEAVATLVNIAALDRPEDG

IYSLPGILAETVGRDIEDVYRGSDKGILADVELLRQITEASRGAITTFVDRTTNKKGQVV

DVAEKLSSMLGFGLEPWVQYLSKTKFLRADREKLRTLFEFLGECLKLVVADNELASLKLA

LEGSYVEPGPGGDPIRNPKVLPTGKNIHALDPQAIPTAAAMQSAKVVVDRLIERQKADNG

KKYPETVALVLWGTDNIKTYGESLAQVLWMIGVRPVADTFGRVNRVEPVSLEELGRPRID

VVVNCSGVFRDLFINQMNLLDRAVKMVAELDEPEDQNYVRKHAMQQAQELGVPVREAATR

VFSNASGSYSSNVNLAVENSSWNDEKQLQDMYVSRKSFAFDCDAPGAGMTEKRKVFEMVL

GTADATFQNLDSSEISLTDVSHYFDSDPTNLVQSLRKDGRKPSAYIADTTTANAQVRTLA

ETVRLDARTKLLNPKWYEGMMGSGYEGVREIEKRLTNTVGWSATSGQVDNWVYEEANDTF

IKDDKMLQRLMETNPNSFRKLVQTFLEASGRGYWETSEENLERLRQLYSEVEDKIEGIER

>A0A6I9SX28

MASLVSSPFTLPNSKVEHLSSFSQKNYFLHSFLPKKFNNTNTQSSHKFKCAAIGNGLFTQ

TTPEVRRIVPEKSSNGLPTVKIVYVVLEAQYQSSLTAAVQSLNQSGQYASFEVVGYLVEE

LRDANTYKTFCKDLEDANIFIGSLIFVEELALKVKDAVEKERERLDAVLVFPSMPEVMRL

NKLGSFSMSQLGQSKSPFFQLFKKKNKSSAGFADSMLKLVRTLPKVLKYLPSDKAQDARM

YILSLQFWLGGSPDNLVNFLKMISGSYVPALKGTKIEYSDPVLYLDSGIWHPLAPCMYDD

VKEYLNWYATRRDTNEQLKNPNSPVIGLVLQRSHIVTGDESHYVAVIMELEARGAKVIPI

FAGGLDFSGPVERYFIDPITKKPMVNSVVSLTGFALVGGPARQDHPRAVEALMKLDVPYI

VALPLVFQTTEEWLNSTLGLHPIQVALQVALPELDGGMEPIVFAGRDPRTGKSHALHKRV

EQLCTRAIKWAELKRKSKAEKKLAITVFSFPPDKGNVGTAAYLNVFASIYSVLKDLKSDG

YSVEGLPETAEALIEDIIHDKEAQFNSPNLNIAYKMGVREYQNLTPYATALEENWGKPPG

NLNSDGENLLVYGKQYGNVFIGVQPTFGYEGDPMRLLFSKSASPHHGFAAYYSFVEKIFK

ADAVLHFGTHGSLEFMPGKQVGMSDVCYPDSLIGNIPNVYYYAANNPSEATVAKRRSYAN

TISYLTPPAENAGLYKGLKQLSELISSYQSLKDTGRGPQIVNSIISTARQCNLDKDVDLP

EEGVEISAKERDLVVGKVYSKIMEIESRLLPCGLHVIGEPPSAMEAVATLVNIAALDRPE

DGISSLPSILAETVGREIEDVYRGSDKGILRDVELLRQITEASRGAISAFVERTTNKKGQ

VVDVADKLTSILGFGLNEPWIQYLSNTKFYRADREKLRVLFQFLGECLKLVVADNELGSL

KQALEGKYVEPGPGGDPIRNPKVLPTGKNIHALDPQAIPTTAAMQSAKVVVDRLLERQKI

DNGGKYPETVALVLWGTDNIKTYGESLAQVLWMIGVRPVADTFGRVNRVEPVSLEELGRP

RVDVVVNCSGVFRDLFINQMNLLDRAVKMVAELDEPEDQNYVRKHALEQAKTLGVEVREA

ASRIFSNASGSYSSNINLAVENSSWNDEKQLQDMYLSRKSFAFDSDAPGAGMTEKRKIFE

MALSTADATFQNLDSSEISLTDVSHYFDSDPTNLVQNLRKDGKKPSAYIADTTTANAQVR

TLSETVRLDARTKLLNPKWYEGMLSTGYEGVREIEKRLTNTVGWSATSGQVDNWVYEEAN

TTFIQDEQMLNRLMSTNPNSFRKLIQTFLEANGRGYWETSAENIERLRQLYSEVEDKIEG

IDR

>A0A6J0MUB4

MASSSLMYSPFTLSASKAEHLSSLSNTTKHSFLLRRGKSKQTKQTKSLFKVKSSVSGGNG

LFTQTNPEVRRIVPVKRDNVPTVKIVYVVLEAQYQSSLSEAVQQLNKTSRFASYEVVGYL

VEELRDKNTYSSFCKDLEDANIFIGSLIFVEELALKVKDAVEKERDRMDAVLVFPSMPEV

MRLNKLGSFSMSQLGQSKSPFFQLFKRKKGTSSAGFADSMLKLVRTLPKVLKYLPSDKAQ

DARLYILSLQFWLGGSPDNLQNFVKMISGSYVPALKGVKIEYSDPVLFLDTGIWHPLAPT

MYDDVKEYLNWYDTRRDTNPSLKKKDATVIGLVLQRSHIVTGDDSHYVAVIMELEARGAK

VIPIFAGGLDFSGPVERYFVDPVTKQPIINSAVSLTGFALVGGPARQDHPRAIEALKTLD

VPYLVGVPLVFQTTEEWLNSTLGLHPIQVALQVALPELDGGMEPIVFAGRDPRTGKSHAL

HKRVEQLCIRAIRWGELKRKTKAEKKVAITVFSFPPDKGNVGTAAYLNVFASIFSVLKDL

KRDGYNVEGLPETAETLIEEILHDKEAQFSSPNLNVAYKMGVREYQTLTPYATALEENWG

KPPGNLNSDGENLLVFGKTYGNVFIGVQPTFGYEGDPMRLLFSKSASPHHGFAAYYSYVE

KIFKADAVLHFGTHGSLEFMPGKQVGMSDACFPDSLIGNIPNVYYYAANNPSEATIAKRR

SYANTISYLTPPAENAGLYKGLKQLSELISSYQSLKDTGRGPQIVSSIISTAKQCNLDKD

VDLPDEGTDLSVKERDLVVGKVYSKIMEIESRLLPCGLHVIGEPPSAMEAVATLVNIAAL

DRAEDNISSLPSILAECVGREIEDVYRGSDKGILTDVELLKQITDASRGAVSAFVEKTTN

SKGQVVNVSDKLTSILGFGINEPWVEYLSNTKFYRANRDKLRTVFGFLGECLKLVVMDNE

LGSLMTALEGKYVEPGPGGDPIRNPKVLPTGKNIHALDPQAIPTTAAMASAKIVVDRLVE

RQRMENEGKYPETIALVLWGTDNIKTYGESLGQVLWMIGARPVADGLGRVNRVEPVSLEE

LGRPRIDVVVNCSGVFRDLFINQMNLLDRAIKMVAELDEPVEMNYVRKHAMEQAATLGVD

IREAATRVFSNASGSYSANISLAVENSSWNDEQQLQDMYLSRKSFAFDSDAPGAGMAEKK

EVFQMALMTAEVTFQNLDSSEISLTDVSHYFDSDPTNLVQSLRKDKKKPSAYIADTTTAN

AQVRSLSETVRLDARTKLLNPKWYEGMMSSGYEGVREIEKRLTNTVGWSATSGQVDNWVY

EEANTTFIKDEEMLNRLMNTNPNSFRKMIQTFLEANGRGYWETSEDNIEKLKDLYSQVED

KIEGIDR

>A0A6J1DH39

MASLVSSPFLAASKSDHQLSSLSQKHYFLHSFLPKKTHLPVSSKSAIRVKCTAIGNGLFT

QTSPEVRRVVPDNTAGLPTVKVVYVVLEAQYQSSLTAAVQSLNKNKTHASFEVVGYLVEE

LRDESTYKSFCKDLEDANIFIGSLIFVEELALKVKAAVEKERECLDAVLVFPSMPEVMRL

NKLGSFSMSQLGQSKSPFFQLFKKKKQSAGFADSMLKLVRTLPKVLKYLPSDKAQDARLY

ILSLQFWLGGSPDNLQNFLKMISGSYVPALKGAKVEYSEPVLYLDSGIWHPLAPCMYDDV

KEYLNWYGTRRDANEKLKDPNAPVIGLILQRSHIVTGDESHYVAVIMELEAKGAKVIPIF

AGGLDFSGPVERYLVNPVTKKPFVHSVVSLTGFALVGGPARQDHPRAVEALTKLDVPYIV

ALPLVFQTTEEWLNSTLGLHPIQVALQVALPELDGGMEPIVFSGRDPRTGKSHALHKRVE

QLCTRAIKWAELKRKSKAEKKLAITVFSFPPDKGNVGTAAYLNVFSSIFSVLKDLKRDGY

NVEGLPETSEALIEDVIHDKEAQFNSPNLNIAYKMNVREYQKLTPYSTALEENWGKPPGH

LNSDGENLLVYGKQYGNVFIGVQPTFGYEGDPMRLLFSKSASPHHGFAAYYSYVENIFKA

DAVLHFGTHGSLEFMPGKQVGMSDVCYPDSLIGNIPNVYYYAANNPSEATVAKRRSYANT

ISYLTPPAENAGLYKGLKQLSELISSYQSLKDTGRGAQIVSSIVSTARQCNLDKDVELPE

EGEEIPAKERDQVVGKVYSKIMEIESRLLPCGLHIIGEPPSAMEAVATLVNIAALDRPED

EISSLPSILASTVGRNIEDVYRGNDRGVLKDVELLRQITEASRGAISAFVERTTNKKGQV

VDVGDKLSSILGFNISEPWVQYLSNTKFYRADREKLRKLFEFLAECLKLVVMDNELGSLK

QALEGKYVEPGPGGDPIRNPKVLPTGKNIHALDPQAIPTTAAMQSAKVVVDRLIERQKVE

NGGKYPETIALVLWGTDNIKTYGESLAQVLWMIGVLPVADTFGRVNRVEPVSLEELGRPR

IDVVVNCSGVFRDLFINQMNLLDRAVKMVAELDEPEEQNFVRKHAIEQAQALGIGVREAA

TRVFSNASGSYSSNINLAVENSSWNDEKQLQDMYLSRKSFAFDCDAPGAGMMEKRNVFEM

ALSTADATFQNLDSSEISLTDVSHYFDSDPTNLVQGLRKDGKKPSAYIADTTTANAQVRT

LAETVRLDARTKLLNPKWYEGMMSSGYEGVREIEKRLTNTVGWSATSGQVDNWVYEEANS

TFIQDEEMLNRLMKTNPNSFRKLVQTFLEANGRGYWETSEENIEKLRQLYSEVEDKIEGI

DR

>A0A6J1ELQ1

MASLMSSPFLPASKSELQLSSLSQKHFFLHSFLPKKTHLAISSKSAVKVKCVATGNGLFT

QTSPEVRRVVPDNTNGLPTVKIVYVVLEAQYQSSLTAAVLALNKNKTHANFQVVGYLVEE

LRDVSTYNTFCKDVEDANIFIGSLIFVEELALKVKAAVEKERDRLDAVLVFPSMPEVMRL

NKLGSFSMSQLGQSKSPFFQLFKKKKQSAGFADSMLKLVRTLPKVLKYLPSDKAQDARLY

ILSLQFWLGGSPDNLQNFLKMISGSYVPALKGAKIEYSEPVLYLDTGIWHPLAPCMYDDV

KEYLNWYGTRRDANEKLKDSKAPVIGLILQRSHIVTGDESHYVAVIMELEARGAKVIPIF

AGGLDFSGPVERYLVDPVTKKPFVHSVVSLTGFALVGGPARQDHPRAVEALTKLDVPYIV

ALPLVFQTTEEWLNSTLGLHPIQVALQVALPELDGGMEPIVFSGRDPRTGKSHALHKRVE

QLCTRAIKWAELKRKSKAEKKLAITVFSFPPDKGNVGTAAYLNVFSSIFSVLKDLKKDGY

NVEGLPETSEALIEDVIHDKEAQFNSPNLNIAYKMNVREYQQLTPYSSTLEENWGKPPGH

LNSDGENLLVYGKQYGNVFIGVQPTFGYEGDPMRLLFSKSASPHHGFAAYYSYVENIFKA

DAVLHFGTHGSLEFMPGKQVGMSDVCYPDSLIGNIPNVYYYAANNPSEATVAKRRSYANT

ISYLTPPAENAGLYKGLKQLSELISSYQSLKDTGRGAQIVSSIVSTARQCNLDKDVELPE

EGEEIPAKDRDLVVGKVYSKIMEIESRLLPCGLHVIGEPPSALEAVATLVNIAALDRPED

DISSLPSILANTVGRNIEDLYRGNDKGVLKDVELLRQITEASRGAISLFVERSTNSKGQV

VDVGDKLTSILGFGINEPWVQYLSNTKFYRADREKLRKLFEFLAECLKLIVTDNELGSLK

QALEGKYVEPGPGGDPIRNPKVLPTGKNIHALDPQAIPTTAAMQSAKVVVERLIERQKAE

NGGKYPETIALVLWGTDNIKTYGESLAQVLWMIGVMPVADTFGRVNRVEPVSLEELGRPR

IDVVVNCSGVFRDLFINQMNLLDRAVKMVAELDEPEELNFVRKHATEQAQALGIEVREAA

TRVFSNASGSYSSNINLAVENSSWNDEKQLQDMYLSRKSFAFDCDAPGAGMMEKRKVFEM

ALSTADATFQNLDSSEISLTDVSHYFDSDPTNLVQGLRKDGKKPNAYIADTTTANAQVRS

LAETVRLDARTKLLNPKWYEGMMSSGYEGVREIEKRLTNTVGWSATSGQVDNWVYEEANT

TFIQDEEMLNRLMKTNPNSFRKLVQTFLEANGRGYWETSEENIEKLRQLYSEVEDKIEGV

DR

>A0A6J1HV67

MASLMSSPFLPASKSELQLSSLSQKHFFLHSFLPKKTHLAISSKSAVKVKCVATGNGLFT

QTSPEVRRVVPDNTNGLPTVKIVYVVLEAQYQSSLTAAVLALNKNKTHANFQVVGYLVEE

LRDVSTYNTFCKDVEEANIFIGSLIFVEELALKVKAAVEKERDRLDAVLVFPSMPEVMRL

NKLGSFSMSQLGQSKSPFFQLFKKKKQSAGFADSMLKLVRTLPKVLKYLPSDKAQDARLY

ILSLQFWLGGSPDNLQNFLKMISGSYVPALKGAKIEYSEPVLYLDTGIWHPLAPCMYDDV

KEYLNWYGTRRDANEKLKDSKAPVIGLILQRSHIVTGDESHYVAVIMELEARGAKVIPIF

AGGLDFSGPVERYLVDPVTKKPFVHSVVSLTGFALVGGPARQDHPRAVEALTKLDVPYIV

ALPLVFQTTEEWLNSTLGLHPIQVALQVALPELDGGMEPIVFSGRDPRTGKSHALHKRVE

QLCTRAIKWAELKRKSKAEKKLAITVFSFPPDKGNVGTAAYLNVFSSIFSVLKDLKKDGY

NVEGLPETSEALIEDVIHDKEAQFNSPNLNIAYKMNVREYQQLTPYSSALEENWGKPPGH

LNSDGENLLVYGKQYGNVFIGVQPTFGYEGDPMRLLFSKSASPHHGFAAYYSYVENIFKA

DAVLHFGTHGSLEFMPGKQVGMSDVCYPDSLIGNIPNVYYYAANNPSEATVAKRRSYANT

ISYLTPPAENAGLYKGLKQLSELISSYQSLKDTGRGAQIVSSIVSTARQCNLDKDVELPE

EGEEIPAKDRDLVVGKVYSKIMEIESRLLPCGLHVIGEPPSAMEAVATLVNIAALDRPED

DISSLPSILANTVGRNIEDLYRGNDKGVLKDVELLRQITEASRGAISSFVERSTNSKGQV

IDVGDKLTSILGFGINEPWVQYLSNTKFYRADREKLRKLFEFLAECLKLIVTDNELGSLK

QALEGKYVEPGPGGDPIRNPKVLPTGKNIHALDPQAIPTTAAMQSAKVVVERLIERQKAE

NGGKYPETIALVLWGTDNIKTYGESLAQVLWMIGVMPVADTFGRVNRVEPVSLEELGRPR

IDVVVNCSGVFRDLFINQMNLLDRAVKMVAELDEPEELNFVRKHATEQAQALGIEVREAA

TRVFSNASGSYSSNINLAVENSSWNDEKQLQDMYLSRKSFAFDCDAPGAGMMEKRKVFEM

ALSTADATFQNLDSSEISLTDVSHYFDSDPTNLVQGLRKDGKKPNAYIADTTTANAQVRS

LAETVRLDARTKLLNPKWYEGMMSSGYEGVREIEKRLTNTVGWSATSGQVDNWVYEEANT

TFIQDEEMLNRLMKTNPNSFRKLVQTFLEANGRGYWETSEENIEKLRQLYSEVEDKIEGV

DR

>A0A6P4B7I9

MASLVSSPFTLSGSKEALSSLSQKHYFLHSFLPRKINPTYSKFTLRVKCAAIGNGLFTQT

TPEVRRIVPSNNQGLPTVKIVYVVLEAQYQSSLTAAVQALNSNQTDASYEVVGYLVEELR

DEATYKTFCKDLEDANIFIGSLIFVEELALKVKAAVEKERDRLDAVLVFPSMPEVMRLNK

LGSFSMSQLGQSKSPFFQLFKRKKQSSAGFADSMLKLVRTLPKVLKYLPSDKAQDARLYI

LSLQFWLGGSPDNLQNFLKMISGSYVPALKGTKIKYSDPVLYLDSGVWHPLAPCMYDDVK

EYMNWYDTRRDANEKLKSPNAPIIGLVLQRSHIVTGDESHYVAVIMELEARGAKVIPIFA

GGLDFSGPVERYLIDPVTKKPFVHSVVSLTGFALVGGPARQDHPRAIEALMKLDVPYIVA

LPLVFQTTEEWLNSTLGLHPIQVALQVALPELDGGMEPIVFAGRDPRTGKSHALHKRVEQ

LCTRAIRWAELKRKSKTEKRVAITVFSFPPDKGNVGTAAYLNVFSSIFSVLQDLKRDGYN

VEGLPETSEALIEEVIHDKEAQFSSPNLNVAYKMGVREYQSLTPYANALEESWGKPPGNL

NSDGENLLVYGKQYGNVFIGVQPTFGYEGDPMRLLFSKSASPHHGFAAYYSFVEKIFKAD

AVLHFGTHGSLEFMPGKQVGMSDVCYPDSLIGNIPNVYYYAANNPSEATIAKRRSYANTI

SYLTPPAENAGLYKGLKQLSELISSYQSLKDTGRGPQIVSSIISTAKQCNLDKDVELPDE

GQEITAQERDLVVGKVYSKIMEIESRLLPCGLHVIGEPPSAIEAIATLVNIAALDRPEDE

ISSLPSILAETVGRNIEDIYRGSDKGILKDVELLRQITEASRGAISAFVERTTNKKGQVV

DVADKLSSILGFGINEPWVQYLSNTKFYRADRDKLRKLFEFLGECLKLIVADNELGSLKQ

ALAGKYVEPGPGGDPIRNPKVLPTGKNIHALDPQAIPTTAAMQSAKVVVDRLIERQKADN

GGNYPETVALVLWGTDNIKTYGESLAQVLWMIGVRPVADTFGRVNRVEPVDLEELGRPRI

DVVVNCSGVFRDLFINQMNLLDRAVKMVAELDEPAEMNYVRKHAIEQAEALGIDVREAAT

RVFSNASGSYSSNINLAVENSSWNDEKQLQDMYLSRKSFAFDCDAPGAGMTEKRNVFEMA

LSTADATFQNLDSSEISLTDVSHYFDSDPTNLVQNLRKDGKKPSAYVADTTTANAQVRTL

SETVRLDARTKLLNPKWYEGMMSSGYEGVREIEKRLTNTVGWSATSGQVDNWVYEEANTT

FIEDEEMLNRLMNTNPNSFRKMVQTFLEANGRGYWETSEKR

>A0A6P5SBG3

MASLVSSPFTLPNTKADQLSSLSRKQYFLHSFLPKKVNQSSLKSSLKVKCAMGNGLFTQT

TQEVRRIVPENKQGLPTVKIVYVVLEAQYQSSLTAAVQALNSNSKYASFEVVGYLVEELR

DAETYKMFCQDLEDANIFIGSLIFVEELAVKVRDAVEKERDRLDAVLVFPSMPEVMRLNK

LGSFSMSQLGQSKSPFFQLFKRKKQSAGFADSMLKLVRTLPKVLKYLPSDKAQDARLYIL

SLQFWLGGSPDNLQNFLKMISGSYVPALKGEKIPYSDPVLFLDSGIWHPLAPCMYDDVKE

YLNWYGTRKDANEKLKSPNAPVVGLILQRSHIVTGDESHYVAVIMELEARGAKVIPIFAG

GLDFSGPVERFLIDPVTKKPFIHSAISLTGFALVGGPARQDHPRAVEALMKLDVPYIVAL

PLVFQTTEEWLNSTLGLHPIQVALQVALPELDGGMEPIVFAGRDPRTGKSHALHKRVEQL

CTRAIRWGELKRKAKAEKKLAITVFSFPPDKGNVGTAAYLNVFSSIFAVLQELKRDGYNV

ENLPETSEALIEDVIHDKEAQFSSPNLNVAYKMGVREYQSLTPYATALEENWGKPPGNLN

SDGENLLVYGKQYGNVFIGVQPTFGYEGDPMRLLFSKSASPHHGFAAYYSFVEKIFQADA

VLHFGTHGSLEFMPGKQVGMSDACFPDSLIGNIPNVYYYAANNPSEATIAKRRSYANTIS

YLTPPAENAGLYKGLKQLSELISSYQSLKDTGRGSQIVSSIISTAKQCNLDKDVELPEEG

VEISAKERDLVVGKVYNKIMEIESRLLPCGLHVIGEPPTAMEAVATLVNIAALNRPEEGI

TSLPDILADTAGRDIEDIYRGSDKGILKDVELLKQITDTSRGAISAFVERTTNEKGQVVD

VKDKLSSILGFGINEPWVQYLSNTKFYRADRDKLRTLFMFLGECLKLIVADNEIGSLKQA

LEGKYVEPGPGGDPIRNPKVLPTGKNIHALDPQSIPTTAAMQSAKIVVERLIERQKIDNG

GKYPETIALVLWGTDNIKTYGESLAQVLWMVGVMPVADAFGRVNRVEIVSLEELGRPRID

VVVNCSGVFRDLFINQMNLLDRAVKMVAELDEPVEQNFIRKHALEQAETLGIGVREAATR

IFSNASGSYSSNINLAVENSSWNDEKQLQDMYLSRKSFAFDSDAPGTGMAENRKVFEMAL

STAEATFQNLDSSEISLTDVSHYFDSDPTNLVQNLRKDGKKPSAYIADTTTANAQVRTLS

ETVRLDARTKLLNPKWYEGMLSSGYEGVREIEKRLTNTVGWSATSGQVDNWVYEEANSTF

IQDEEMLNRLMKTNPNSFRKLVQTFLEANGRGYWDTDEQNIEKLKELYSEVEDKIEGIDR

>A0A6P5YP51

MASFVYSPFTFPSSKPDQLSSLSQKHFFLHSFLPKRTNNQPNSKSSLKVKCGAIGNGLFT

QTTPEVRRIVPEKKDDLPTVKIVYVVLEAQYQSSLSSAVHSLNQNSNFASFGVVGYLVEE

LRDENTYKTFCQDLEDANIFIGSLIFVEELALKVKAAVEKERDRLDAVLVFPSMPEVMRL

NKLGSFSMSQLGQSKSPFFQLFKRKKQGAGFADGMLKLVRTLPKVLKYLPSDKAQDARLY

ILSLQFWLGGSPDNLQNFLKMISGSYVPALKRTKIDYSDPVLFLDSGIWHPLAPCMYDDV

KEYLNWYGTRRDVNEKLRGPNAPLIGLVLQRSHIVTGDESHYVAVIMELEARGAKVIPIF

AGGLDFSGPVERFLIDPVTKEPMVNSVVSLTGFALVGGPARQDHPRAVEALMKLDVPYIV

ALPLVFQTTEEWLNSTLGLHPIQVALQVALPELDGGMEPIVFAGRDPRTGKSHALHKRVE

QLCTRAIKWAELKRKSKTEKKLAVTVFSFPPDKGNVGTAAYLNVFASIYSVLKDLQKDGY

NVEGLPETAETLIEDVIHDKEAQFNSPNLNVAYKMSVREYQNLTPYAPALEENWGKPPGN

LNSDGENLLVYGKQYGNVFIGVQPTFGYEGDPMRLLFSKSASPHHGFAAYYSFVEKIFNA

DAVLHFGTHGSLEFMPGKQVGMSDVCYPDSLIGNIPNVYYYAANNPSEATIAKRRSYANT

ISYLTPPAENAGLYKGLKQLSELISSYQSLKDSGRGQQIVNSIISTAKQCNLDKDVELPE

EGEEISAKERDLVVGKVYSKIMEIESRLLPCGLHVIGEPPSAMEAVATLVNIAALDRPED

GILSLPSVLAKTVGRNIEDVYRGSDKGILKDVELLRQITEASRGAIYAFVEQTTNKKGQV

VDVADKLSSILGFGINEPWIQYLSNTKFYRADREKLRVLFEFLGECLKLVVADNELGSLK

QALEGKYVEPGPGGDPIRNPKVLPTGKNIHALDPQAIPTTAAMQSAKIVVDRLIDRQKID

NGGKYPETIALVLWGTDNIKTYGESLAQVLWMIGVRPVADTFGRVNRVEPVSLEELGRPR

IDVVVNCSGVFRDLFINQMNLLDRAVKMVAELDEPVEQNYVKKHALEQAKALGIEVREAA

TRVFSNASGSYSSNVNLAVENSSWNDEKQLQDMYLSRKSFAFDCDAPGAGMTEKRKVFEM

ALSTADATFQNLDSSEISLTDVSHYFDSDPTNLVHNLRKDGKKPSAYIADTTTANAQVRT

LAETVRLDARTKLLNPKWYEGMMSSGYEGVREIEKRLTNTVGWSATSGQVDNWVYEDANS

TFIQDENMLNRLMSTNPNSFRKLVQTFLEANGRGYWETSEENIERLRQLYSEVEDKIEGI

DR

>A0A6P6SUR7

MASLISSPFQIPSSTVDQLSSISQKRYFLHSFLPKKSSPTNSNSALKFKCAAIGNGLFTQ

TTPEVRRIVPENNKGLPTVKVVYVVLEAQYQSTLTAAVQTLNQNGKFASFEVVGYLVEEL

RDENTYKTFCKDLEDANIFIGSLIFVEELALKVKAAVEKERNRLDAVLVFPSMPEVMRLN

KLGSFSMSQLGQSKSPFFQLFKKKNKSSAGFADSMLKLVRTLPKVLKYLPSDKAQDARMY

ILSLQFWLGGSPDNLVNFLKMISGSYVPALKGTKIEYSDPVLYLDSGIWHPLAPCMYDDV

KEYLNWYGTRRDANEKLKSPTAPIIGLVLQRSHIVTGDESHYVAVIMELEARGAKVIPIF

AGGLDFSGPVEKYFIDPVTKRPFVNSVVSLTGFALVGGPARQDHPRAVEALRKLDVPYIV

ALPLVFQTTEEWLNSTLGLHPIQVALQVALPELDGGMEPIVFAGRDPRTGKSHALHKRVE

QLCTRAIKWAELKKKSKTEKKLAITVFSFPPDKGNVGTAAYLNVFASIYSVLKDLQKDGY

NVEGLPETGEALIEDIIHDKEAQFNSPNLNIAYKMNVREYQKLTPYATALEENWGKPPGN

LNSDGENLLVYGKQYGNVFIGVQPTFGYEGDPMRLLFSKSASPHHGFAAYYSFVEKIFQA

DAVLHFGTHGSLEFMPGKQVGMSDVCYPDSLIGNIPNVYYYAANNPSEATIAKRRSYANT

ISYLTPPAEYAGLYKGLKQLSELISSYQSLKDTGRGSQIVSSIISTARQCNLDKDVDLPE

EGEEISATERDLVVGKVYSKIMEIESRLLPCGLHVIGEPPSAMEAVATLVNIAALDRPED

GISSLPSILAQTVGRDMEDVYRGSDKGILRDVELLRQITEASRGAITAFVERTTNKKGQV

VDVADKLSAILGFGINEPWAQYLSTTKFYRADREKLRVLFQFLGECLKLVVADNELGSLK

QALEGKYVEPGPGGDPIRNPKVLPTGKNIHALDPQAIPTTAAMQSAKVVVDRLLERQKAD

NGGKYPETVALVLWGTDNIKTYGESLAQVLWMIGVQPVADTFGRVNRVEPVSLEEIGRPR

IDVVVNCSGVFRDLFINQMNLLDRAVKMVAELDEPEDQNYVRKHALEQAKELGIDVREAA

TRVFSNASGSYSSNVNLAVENSSWNDEKQLQDMYLSRKSFAFDCDAPGAGMTEKRKVFEM

ALSTADATFQNLDSSEISLTDVSHYFDSDPTNLVQNLRTDGKKPNAYIADTTTANAQVRT

LSETVRLDARTKLLNPKWYEGMLSSGYEGVREIEKRLTNTVGWSATSGQVDNWVYEEANT

TFIEDQEMLNRLMSTNPNSFRKLVQTFLEANGRGYWETSEENIERLRQLYSEVEDKIEGI

DR

>A0A8B8L2E7

MASLVSLSFSLPNSKPDQLSSLAQKHLFLHSFLPKKASYYGSSNSTLRVKCAAIGNGLFT

QTTPEVRRIVPENDQNLPTVKIVYVVLEAQYQSSLSAAVRALNSERKNVSFEVVGYLVEE

LRDESTYKTFCKDLEDANIFIGSLIFVEELALKVKVAVEKERDRLNAVLVFPSMPEVMRL

NKLGSFSMSQLGQSKSPFFQLFKRKKPQSAGFADNMLKLVRTLPKVLKYLPSDKAQDARL

YILSLQFWLGGSPDNLQNFLKMVSGSYVPALKGTKIEYSEPVLYLDSGIWHPLAPCMYDD

VKEYLNWYGTRRDANEKLKSPNAPVIGLILQRSHIVTGDEGHYVAVIMELEARGAKVIPI

FAGGLDFSGPVEKFLIDPITKKPFVNSVVSLTGFALVGGPARQDHPRAVEALRRLDVPYI

VALPLVFQTTEEWLNSTLGLHPIQVALQVALPELDGGMEPIVFAGRDPKTGKSHALHKRV

EQLCIRAIRWAELKRKSKAEKKLAITVFSFPPDKGNVGTAAYLNVFSSIYSVLEELKRDG

YNVDGLPDAPEALIEDVIHDKEAQFSSPNLNIAYKMSVREYQNLTPYASALEENWGKPPG

NLNADGENLLVYGKQYGNVFIGVQPTFGYEGDPMRLLFSKSASPHHGFAAYYSFVEKIFK

ADAVLHFGTHGSLEFMPGKQVGMSDVCYPDSLIGNIPNVYYYAANNPSEATIAKRRSYAN

TISYLTPPAENAGLYKGLKQLSELISSYQSLKDTGRGPQIVSSIISTAKQCNLDKDVSLP

DEGEEISPKERDLVVGKVYSKIMEIESRLLPCGLHVIGEPPSALEAVATLVNIAALDRPE

DGISSLPAILAYTVGREIEDVYRGSDKGILKDVELLRQITEASRGAVTAFVERTTNNKGQ

VVDVADKLTSILGFGVNEPWIQYLSNTKFYRADREKLRTLFGFLGECLKLVVADNELGSL

KQALEGKYVEPGPGGDPIRNPKVLPTGKNIHALDPQAIPTTAAMQSAKIVVDRLIERQKA

DNGGKYPETVALVLWGTDNIKTYGESLAQVLWMIGVTPVADTFGRVNRVESVSLEELGRP

RIDVVVNCSGVFRDLFINQMNLLDRAVKMVAELDEPAEQNYVRKHALEQAQALGIEVREA

ATRVFSNASGSYSSNINLAVENSSWNDEKQLQDMYLSRKSFAFDSDAPGAGMTEKRKVFE

MALSTADATFQNLDSSEISLTDVSHYFDSDPTNLVQNLRKDGKKPSSYIADTTTANAQVR

TLSETVRLDARTKLLNPKWYEGMLSSGYEGVREIEKRLTNTVGWSATSGQVDNWVYEEAN

TTFIQDEQMLNKLMNTNPNSFRKLVQTFLEANGRGYWETSEENIEKLRQLYSEVEDKIEG

IDR

>A0A8B8NCY4

MASLLSSQFALPAPKADQLSSLSQKRYFLHSFLPRKTNPARNSSKSALKLKCAVLGNGLF

TQTTPEVRRIVPENNENLPTVKVVYVVLEAQYQSSLSAAVLALNKNERYASFEVVGYLVE

ELRDENTYKSFCKDLEDANIFIGSLIFVEELALKVKAAVETERDRLDAVLVFPSMPEVMR

LNKLGSFSMSQLGQSKSPFFQLFKKKKSSAGFADSMLKLVRTLPKVLKYLPSDKAQDARL

YILSLQFWLGGSPDNLQNFLKMISGSYVPALKGTKIEYSDPVLFLDSGIWHPLAPCMYDD

VKEYLNWYGTRKDANEKLKGPNAPVIGLILQRSHIVTGDESHYVAVIMELEARGAKVIPI

FAGGLDFSGPVEKFLINPVTKKPFVHSVISLTGFALVGGPARQDHPRAVEALMKLDVPYI

VAVPLVFQTTEEWLNSTLGLHPIQVALQVALPELDGGMEPIVFAGRDPRTGKSHALHKRV

EQLCTRAIRWAELKRKSKEEKRLAITVFSFPPDKGNVGSAAYLNVFSSIYSVLKDLQKDG

YNVEGLPETSEALIEEVIHDKEAQFSSPNLNVAHKMGVREYYDLTPYATALEENWGKAPG

NLNSDGESLLVYGKQYGNVFIGVQPTFGYEGDPMRLLFSKSASPHHGFAAFYSFVEKIFQ

ADAVLHFGTHGSLEFMPGKQVGMSDACYPDSLIGNIPNVYYYAANNPSEATIAKRRSYAN

TISYLTPPAENAGLYKGLKQLSELISSYQSLKDTGRGPQIVSSIISTAKQCNLDKDVELP

DELEEISANERDLVVGKVYSKIMEIESRLLPCGLHVIGEPPSAMEAVATLVNIAALDRPE

DGISSLPAILAETVGRDIEDVYRGSDKGILKDVELLRQITEASRGAVTAFVEQTTNNKGQ

VVDVTNKLSSILGFGINEPWIQYLSSTKFYRAEREKLRTLFKFLGECLKLVVADNELGSL

KQALEGKYVEPGPGGDPIRNPKVLPTGKNIHALDPQAIPTTAAMQSAKVVVDRLVERQKV

DNGGKYPETVALVLWGTDNIKTYGESLGQVLWMIGVRPVADTFGRVNRVEPVSLEELGRP

RIDVVVNCSGVFRDLFINQMNLLDRAIKMVAELDEPEELNYVRKHALEQAKTLGIDVREA

ATRVFSNASGSYSSNINLAVENSSWNDEKQLQDMYLSRKSFAFDCDAPGAGMTEKRKVFE

MALSTADATFQNLDSSEISLTDVSHYFDSDPTNLVQNLRKDRRKPSAYIADTTTANAQVR

TLAETVRLDARTKLLNPKWYEGMMSSGYEGVREIEKRLTNTVGWSATSGQVDNWVYEEAN

STFVQDEEMLKRLMNTNPNSFRKLLQTFLEANGRGYWETSEQNIERLRQLYSEVEDKIEG

IDR

>A0A9R0HYG3

MSSMVSSAFTLPSTKTDLISSISQKKYILHSFLPKKSTQFTSRSPRNAQIKCVVAGNGQF

TQVSPEVRRIVPLKNNNLPTVKIVYVVLEAQYQSSLTAAVQTLNKTSDVANFEVVGYLVE

ELRDKATYLAFCKDLEDANIFIGSLIFVEELAIKVRDAVEKVRDRMDAVLVFPSMPEVMR

LNKLGSFSMSQLGQSKSPFFQLFKRKKQGAGFADSMLKLVRTLPKVLKYLPSDKAQDARL

YILSLQFWLGGSPDNLVNFVKMISGSYIPALKGMKIEYSDPVLFLDSGVWHPLAPCMYDD

VKEYLNWYGTRRDTSPKLKSSNAPIIGLVLQRSHIVTGDESHYVAVIMEMEARGAKVIPI

FAGGLDFSGPVEKYFIDPITKKPMVNAVVSLTGFALVGGPARQDHPRAVEALMKLNVPYM

VAVPLVFQTTEEWLNSTLGLHPIQVALQVALPELDGGMEPIVFAGRDPRTGKSHALHKRV

DQLCTRAIRWAELKRKTKADKKLAITVFSFPPDKGNVGTAAYLNVFASIFAVLSDLKKDG

YSIDGLPESAEELIEEVIHDKEAQFSSPNLNVAYKMSVREYQDLTPYATLLEENWGKAPG

NLNSDGENLLVYGKQYGNVFIGVQPTFGYEGDPMRLLFSKSASPHHGFAAYYSFVEKIFG

ADAVLHFGTHGSLEFMPGKQVGMSDACFPDSLIGNIPNVYYYAANNPSEATIAKRRSYAN

TISYLTPPAENAGLYKGLKQLSELISSYQSLKDTGRGQQIVSSIISTARQCNLDKDVDLP

DEGAEISAKDRDLVVGKVYSKIMEIESRLLPCGLHIIGEPPTALEAVATLVNIAALDRPE

EGISALPSILSETVGRNIEDLYRGNDKGILKDVELLKQITETTRGAVTAFVERATNDKGQ

VVNANDKLTSILGFGINEPWIQYLSNTKFYGADREKLRILFGFLGDCLKLIVADNELGAL

KQALEGRFVEPGPGGDPIRNPKVLPTGKNIHALDPQAIPTTAAMQSAIVVVDRLLERQKA

DNGGKFPETVALVLWGTDNIKTYGESLGQVLWMIGVRPVADTFGRVNRVEPVSLEELGRP

RIDVVVNCSGVFRDLFINQMNLLDRAIKMVAELDEPEEQNYVRKHALEQAKSLGVDVREA

ATRVFSNASGSYSSNINLAVENSSWNDEKQLQDMYLSRKSFAFDSDAPGAGMMEKRQVFE

MALSTADATFQNLDSSEISLTDVSHYFDSDPTNLVQGLRKDGKKPSAYIADTTTANAQVR

TLSETVRLDARTKLLNPKWYEGMLSTGYEGVREIEKRLTNTVGWSATSGQVDNWVYEEAN

TTFIQDEEMLKRLMNTNPNSFRKLLQTFLEANGRGYWETSEDNIEKLKQLYSEVEDKIEG

IDR

>A0A9Y1PUQ3

MASLTSTPFTLSTSEHLSSVFQKHTFLHSFLPRRQQQPNGGGRDLRVRCAAVGNGLFTQT

KPEVRRIVPPPDQTWGLPRVKIVYVVLEAQYQSSLSAAVRAMNADRRYASFEVVGYLVEE

LRDEETYATFCRDLADANVFIGSLIFVEELALKVKAAVEKERDRMDAVLVFPSMPEVMRL

NKLGSFSMSQLGQSKSPFFQLFKRKKQSAGFAESMLKLVRTLPKVLKYLPSDKAQDARLY

ILSLQFWLGGSPENLQNFLKMIAGAYVPALKGTKVKYDDPVLFLDSGIWHPLAPTMYEDV

KEYLNWYGTRKDANQKLKDPNAPVVGLILQRSHIVTGDEGHYVAVIMELEAKGAKVIPIF

SGGLDFSGPVERYLVDPITKKRFVHAVVSLTGFALVGGPARQDHPRAIEALRKLDVPYIV

ALPLVFQTTEEWLNSTLGLHPIQVALQVALPELDGGMEPIVFSGRDPRTGKAHALHKRVE

QLCTRAIRWAELRRRSTEEKKVAITVFSFPPDKGNVGTAAYLNVFSSIFSVLKDLKREGY

DVEGLPETAEELIEDVIHDKEAKFSSPNLNVAYKMSVREYQSLTPYAAALEDSWGKPPGN

LNSDGEHLLVYGKQYGNVFIGVQPTFGYEGDPMRLLFSKSASPHHGFAAYYSFVEKIFQA

DAVLHFGTHGSLEFMPGKQVGMSDACYPDSLIGNIPNIYYYAANNPSEATIAKRRSYANT

ISYLTPPAENAGLYKGLKQLAELISSYQSLKDTGRGPQIVSSIISTARQCNLDKDVELPE

EGAQLKPAERDLVVGRVYSKIMEIESRLLPCGLHVIGEPPSAMEAVATLVNIAALDRPEE

GIYSLPSILAETVGRGIEDVYRGNDRGILADVELLRQITEASRGAITAFVDRTTNKKGQV

VDVAEKLGSMLGFGQTEAWVQYLSRTQFLRADREKLRVLFEFLGECLKLVVADNELGSLK

QALKGKYVEPGPGGDPIRNPKVLPTGKNIHALDPQSIPTEAALQSAKVVVDRLLQRQKAD

NGGNFPETVALVLWGTDNIKTYGESLAQVMWMLGVRPVADTFGRVNRVEPVSLEELGRPR

IDVVVNCSGVFRDLFINQMNLLDRAVKMVAELDEPEEQNYVRKHALQQAAELGTTVRDAA

TRVFSNASGSYSSNVNLAVENSSWNDEKQLQDMYLSRKSFAFDCDAPGAGMMEKRKVFEM

ALATADATFQNLDSSEISLTDVSHYFDSDPTNLVKSLRKDGKKPGAYIADTTTANAQVRT

LAETVRLDARTKLLNPKWYEGMMSSGYEGVREIEKRLTNTVGWSATSGQVDNWVYEEANE

TFVKDEQMLKRLMDTNPNSFRKLVQTFLEASGRGYWDTSDENLERLRQLYAEVEDKIEGI

DR

>A0AAJ6UPX4

MASLVSSPFTLQSTKPNQLSSLSQKHYFLHSFLRKKINQTNFKSSLKVQCAAIGNGLFTQ

TTQEVRRIVPENNQNLPSVKIVYVVLEAQYQSSLTAAVQALNKSSKDASYEVVGYLVEEL

RDESTYKNFCKDLEDANIFIGSLIFVEELALKVKTAVEKERDRLDAVLVFPSMPEVMRLN

KLGSFSMSQLGQSKSPFFQLFKRKKQGAGFADSMLKLVRTLPKVLKYLPSDKAQDARLYI

LSLQFWLGGSPDNLQNFLKMISGSYVPALKGKRIDYSDPVLFLDSGIWHPLAPCMYDDVK

EYLNWYGTRRDANEKLKDPNAPVVGLVLQRSHIVTGDESHYVAVIMELEAKGAKVIPIFA

GGLDFSGPVERFFIDPVIKKPLVNSMVSLTGFALVGGPARQDHPRAVEALSKLDVPYIVA

LPLVFQTTEEWLNSTLGLHPIQVALQVALPELDGGMEPIVFAGRDPRTGKSHALHRRVEQ

LCTRAIRWAELKRKTKTEKKLAITVFSFPPDKGNVGTAAYLNVFSSIFSVLKELKRDGYN

VEGLPETSEALIEDIIHDKEAQFSSPNLNIAYKMGVREYQSLTPYATALEENWGKPPGNL

NSDGENLLVYGKQYGNIFIGVQPTFGYEGDPMRLLFSKSASPHHGFAAYYSFVEKIFKAD

AVLHFGTHGSLEFMPGKQVGMSDVCYPDSLIGNIPNVYYYAANNPSEATIAKRRSYANTI

SYLTPPAENAGLYKGLKQLSELISSYQSLKDTGRGPQIVSSIISTARQCNLDKDVELPDE

GMEISAKERDLVVGKVYSKIMEIESRLLPCGLHVIGEPPSAMEAVATLVNIAALDRPEDG

ISSLPAILAETVGRNIEEVYRGSDKGILMDVELLRQITEAARGAVSAFVQKTTNNKGQVV

DVADKLTSILGFGINEPWVDYLSNTKFYRADRVKLRTLFQFLGECLKLVVADNELGSLKQ

ALEGKYVEPGPGGDPIRNPKVLPTGKNIHALDPQSIPTTAAMQSAKVVVDRLIERQKADN

GGKYPETVALVLWGTDNIKTYGESLAQVLWMIGVMPVADTFGRVNRVEPVSLEELGRPRI

DVVVNCSGVFRDLFINQMNLLDRAAKMVAELDEPADQNYVRKHALEQAEALGVDIREAAT

RVFSNASGSYSSNINLAVENSSWNDEKQLQDMYLSRKSFAFDCDAPGAGMTEKRKVFEMA

LSTAEATFQNLDSSEISLTDVSHYFDSDPTNLVQNMRKDKKKPNAYIADTTTANAQVRTL

AETVRLDARTKLLNPKWYEGMMSSGYEGVREIEKRLTNTVGWSATSGQVDNWVYEEANST

FIQDEEMLNRLMSTNPNSFRKLVQTFLEANGRGYWETSQENIEKLRQLYSEVEDKIEGID

R

>A0AAJ6VJ31

MASLVSSPFTLQSTKSDQLSSLSQKHYFLHSFVPRKINQTSWKSSLKVKCAAIGNGLFTQ

TTQEVRRIVPENNQNLPTVKIVYVVLEAQYQSSLTAAVQALNKSSKDASYEVVGYLVEEL

RDESTYKTFCEDLEDANIFIGSLIFVEELALKVKTAVEKERDRLDAVLVFPSMPEVMRLN

KLGSFSMSQLGQSKSPFFQLFKKKKQGAGFADSMLKLVRTLPKVLKYLPSDKAQDARLYI

LSLQFWLGGSPDNLQNFLKMISGSYVPALKGKKIAYSDPVLFLDSGIWHPLAPCMYDDVK

EYLNWYGTRRDANEKLKDPNAPVVGLVLQRSHIVTGDESHYVAVIMELEARGAKVIPIFA

GGLDFSGPVERYLIDPVTKKPMVNSVISLTGFALVGGPARQDHPRAIEALNKLDVPYIVA

LPLVFQTTEEWLNSTLGLHPIQVALQVALPELDGGMEPIVFAGRDPRTGKSHALHKRVEQ

LCTRAIRWGELKRKSKTEKKLAITVFSFPPDKGNVGTAAYLNVFSSIFSVLKELERDGYN

VEGLPETSEALIEDILHDKEAQFSSPNLNIAYKMGVREYQSLTSYATALEENWGKPPGNL

NSDGENLLVYGKQYGNVFIGVQPTFGYEGDPMRLLFSKSASPHHGFAAYYSFVEKIFKAD

AVLHFGTHGSLEFMPGKQVGMSDACYPDSLIGNIPNVYYYAANNPSEATIAKRRSYANTI

SYLTPPAENAGLYKGLKQLSELISSYQSLKDTGRGPQIVSSIISTARQCNLDKDVELPEE

GEEISAKERDLVVGKVYSKIMEIESRLLPCGLHVIGEPPSAMEAVATLVNIAALDRPEDE

ISSLPSILAETVGRNIEDVYRESDKGILKDVELLRKITEASRGAVSAFVQKTTNKKGQVV

DVADKLSSILGFGINEPWVDYLSSTKFYQADRDKLRTLFRFLGDCLKLIVADNELGSLKQ

ALEGKYVEPGPGGDPIRNPKVLPTGKNIHALDPQSIPTTAAMQSAKVVVDRLIERQKADN

GGKYPETVALVLWGTDNIKTYGESLAQVLWMIGVMPVADTFGRVNRVELVSLEELGRPRI

DVVVNCSGVFRDLFINQMNLLDRAVKMVAELDEPADQNFVRKHALEQAEALGVDIREAAT

RIFSNASGSYSSNINLAVENSSWNDEKQLQDMYLSRKSFAFDCDAPGAGMAEKRKVFEMA

LSTAEATFQNLDSSEISLTDVSHYFDSDPTNLVQNLRKDKKKPNAYIADTTTANAQVRTL

SETVRLDARTKLLNPKWYEGMMSTGYEGVREIEKRLTNTVGWSATSGQVDNWVYEEANST

FIQDEEMLNKLMSTNPNSFRKMVQTFLEANGRGYWETSQDNIEKLRQLYSEVEDKIEGID

R

>C1KIZ6

MASLVSSPFTLPHTKADQLSSLSRKQYFLHSFLPKKVNQSSLKSSLKVKCAMGSYGLFTQ

TTQEVRRIVPENKQGLPTVKIVYVVLEAQYQSSLTAAVQALNSNSKYASFEVVGYLVEEL

RDAETYKMFCQDLEDANIFIGSLIFVEELAVKVRDAVEKERDRLDAVLVFPSMPEVMRLN

KLGSFSMSQLGQSKSPFFQLFKRKKPESAGFADSMLKLVRTLPKVLKYLPSDKAQDARLY

ILSLQFWLGGSPDNLQNFLKMISGSYVPALKGEKIPYSDPVLFLDSGIWHPLAPCMYDDV

KEYLNWYGTRKDANEKLKSPNAPVVGLILQRSHIVTGDESHYVAVIMELEARRAKVIPIF

AGGLDFSGPVERFLIDPVTKKPFIHSAISLTGFALVGGPARQDHPRAVEALMKLDVPYIV

ALPLVFQTTEEWLNSTLGLHPIQVALQVALPELDGGMEPIVFAGRDPRTGKSHALHKRVE

QLCTRAIRWGELKRKAKAEKKLAITVFSFPPDKGNVGTAAYLNVFSSIFAVLQELKRDGY

NVENLPETSEALIEDVIHDKEAQFSSPNLNVAYKMGVREYQSLTPYATALEENWGKPPGN

LNSDGENLLVYGKQYGNVFIGVQPTFGYEGDPMRLLFSKSASPHHGFAAYYSFVEKIFQA

DAVLHFGTHGSLEFMPGKQVGMSDACFPDSLIGNIPNVYYYAANNPSEATIAKRRSYANT

ISYLTPPAENAGLYKGLKQLSELISSYQSLKDTGRGSQIVSSIISTAKQCNLDKDVELPE

EGLEISAKERDLVVGKVYNKIMEIESRLLPCGLHVIGEPPTAMEAVATLVNIAALNRPEE

GITSLPDILAETAGRGIEDLYRGSDKGILKDVELLKQITDTSRGAISAFVERTTNEKGQV

VDVKDKLSSILGFGINEPWVQYLSNTKFYRADRDKLRTLFMFLGECLKLIVADNEIGSLK

QALEGKYVEPGPGGDPIRNPEVLPTGKNIHALDPQSIPTTAAMQSAKIVVERLIERQKID

NGGKYPETIALVLWGTDNIKTYGESLAQVLWMVGVMPVADAFGRVNRVEIVSLEELGRPR

IDVVVNCSGVFRDLFINQMNLLDRAVKMVAELDEPVEQNFIRKHALEQAETLGIGVREAA

TRIFSNASGSYSSNINLAVENSSWNDEKQLQDMYLSRKSFAFDSDAPGVGMAENRKVFEM

ALSTAEATFQNLDSSEISLTDVSHYFDSDPTNLVQSLRKDGKKPSAYIADTTTANAQVRT

LSETVRLDARTKLLNPKWYEGMLSSGHRVVREIEKRLTNTVGWSATSGQVDNWVYEEANT

TFIQDKEMLERLMKTNPNSFRKLVQTFLEANGRGYWDTAEENIEKLKELYQEVEDKIEGI

DR

>C6KF71

MASLVSSPFTLPQTKPDQLSSFTKKHYFLHSFLPKKTNQASSKTTLKVKCAMGSYGLFTQ

TTQEVRRIVPENKQNLPTVKVVYVVLEAQYQSSLTAAVQSLNASNKHASFSVVGYLVEEL

RDDDTYKTFCQDLQDANVFIGSLIFVEELALKVKQAVEKERDRMDAVLVFPSMPEVMRLN

KLGSFSMSQLGQSKSPFFQLFKRKKQGSAGFADSMLKLVRTLPKVLKYLPSDKAQDARLY

ILSLQFWLGGSPDNLQNFLKMIAGSYVPALKGEKIPYSDPVLFLDSGIWHPLAPCMYDDV

KEYLNWYGTRKDANEKLKSPNAPIVGLILQRSHIVTGDESHYVAVIMELEARGAKVIPIF

AGGLDFSGPVERFLIDPVTKKPFIHSAISLTGFALVGGPARQDHPRAIEALMKLDVPYIV

ALPLVFQTTEEWLNSTLGLHPIQVALQVALPELDGGMEPIVFAGRDPRTGKSCSFHKRVE

QLCTRAIRWGELKRKRLRRQKLAITVFSFPPDKGNVGTAAYLNVFSSIFSVLQELKRDGY

NVEGLPETSDALIEEVIHDKEAQFSSPNLNIAYKMGVREYQSLTPYAAALEENWGKPPGN

LNSDGENLLVYGKQYGNVFIGVQPTFGYEGDPMRLLFSKSASPHHGFAAYYSFVEKIFQA

DAVLHFGTHGSLEFMPGKQVGMSDACFPDSLIGNIPNVYYYAANNPSEATIAKRRSYANT

ISYLTPPAENAGLYKGLKQLSELIASYQSLKDTGRGQQIVSSIISTARQCNLDKDVDLPD

EGVEISAKERDLVVGKVYNKIMEIESRLLPCGLHVIGEPPTAMEAVATLVNIAALNRPEE

NIFSLPAILVETVGRDIEDLYRQSDKGILKDVELLKQITDASRGAVSSFVECTTNEKGQV

VDVKNKLTSILGFGINEPWIQYLSNTKFYRADREKLRTLFEYLGECLKLIVADNEIGSLK

QALEGKFVEPGPGGDPIRNPKVLPTGKNIHALDPQSIPTTAAMNSAKVVVERLIERQKLD

NGGKYPETIALVLWGTDNIKTYGESLAQVLWMVGVNPVADGLGRVNKVEVVPLEELGRPR

IDVVVNCSGVFRDLFINQMNLLDRAVKMVAELDEPLEQNFVRKHALEQAETLGIGVREAA

TRIFSASGLYPLHINLAVDNSSWNDEKQVQDMYLSRKSFAFEADAPGAGMAEYRKAFKMA

LSSDDATFQNLDSSEISLSDVSQYFDSDPTNLVQNLRKDGKKPSSYIADTTTANAQVRTL

SETVRLDARTKLLNPKWYEGMLSSGYEGVREIEKRLTNTVGWSATSGQVDNFVYEEANAT

FIKDEEMLNRLMKTNPNSFRKLLQTFLEANGRGYWDTDEENIERLKELYSEVEDKIEGID

R

>D5KXY0

MASLVSSPFTLPTSKVDQLSSFSQKHYFLHSFLPKKTNQANSKSCLRVKCAAIGSGLFTQ

TTPEVRRIVPDNDHGLPTVKVVYVVLEAQYQSALTAAVQTLNSKARYASFQVVGYLVEEL

RDEATYKTFCKGLEDANIFIGSLIFVEELALKVKAAVEKERDRLDAVLVFPSMPEVMRLN

KLGSFSMSQLGQSKSPFFQLFKKKKSSAGFADSMLKLVRTLPKVLKYLPSDKAQDARLYI

LSLQFWLGGSPDNLMNFLKMISGSYVPALKRTKIEYSDPVLFLDSGIWHPLAPCMYDDVK

EYLNWYGTRRDANEKLKGPNAPVIGLVLQRSHIVTGDESHYVAVIMELEARGAKVIPIFA

GGLDFSGPVERFLIDPVTKRPFVNSVVSLTGFALVGGPARQDHPRAVEALMKLDVPYIVA

LPLVFQTTEEWLNSTLGLHPIQVALQVALPELDGGMEPIVFAGRDPRTGKSHALHKRVEQ

LCIRAIRWAELKRKSKAEKKLAITVFSFPPDKGNVGTAAYLNVFDSIFSVLKELKRDGYN

VEGLPETSESLIEDVLHDKEAKFSSPNLNIAYKMGVREYQTLTPYATALEESWGKPPGNL

NSDGENLLVYGKQYGNVFIGVQPTFGYEGDPMRLLFSKSASPHHGFAAYYSFVEKIFKAD

AVLHFGTHGSLEFMPGKQVGMSDVCYPDSLIGNIPNVYYYAANNPSEATIAKRRSYANTI

SYLTPPAENAGLYKGLKQLSELISSYQSLKDTGRGPQIVSSIISTAKQCNLDKDVSLPDE

GEEISAKERDLVVGKVYSKIMEIESRLLPCGLHVIGEPPSAMEAVATLVNIAALNRPEEG

ISSLPAILAETVGRNIEDVYRGSDKGILKDVELLRQITDTSRGAISAFVERTTNKKGQVV

DVADKLTSVFGFGLNEPWVQYLSSTKFYQADREKLRTLFAFLGECLKLVVADNELRSLKQ

ALEGKYVEPGPGGDPIRNPKVLPTGKNIHALDPQSIPTAAALQSAMVVVDRLLERQKADN

GGKYPETVALVLWGTDNIKTYGESLAQVLWMIGVRPVADTFGRVNRVEPVSLEELGRPRI

DVVVNCSGVFRDLFINQMNLLDRAVKMVAELDEPADQNYVRKHALEQAQALGIEVRDAAT

RVFSNASGSYSSNINLAVENSSWNDEKQLQDMYLSRKSLAFDCDAPGAGMTEKRKVFEMA

LSTADATFQNLDSSEISLTDVSHYFDSDPTNLVQGLRKDGKKPNAYIADTTTANAQVRTL

SETVRLDARTKLLNPKWYEGMMSSGYEGVREIEKRLTNTVGWSATSGQVDNWVYEEANST

FIQDEEMLKRLMNTNPNSFRKLVQTFLEANGRGYWETSEDNIEKLRQLYSEVEDKIEGID

R

>M1CLS4

MASLVSSPFTLPNSKVEHLSSISQKHYFLHSFLPKKTNPTFSKSPKKFQCNAIGNGLFTQ

TTQEVRRIVPENLKGLTTVKIVYVVLEAQYQSALTAAVQTLNKNGEFASFEVVGYLVEEL

RDENAYKTFCKDLEDANIFIGSLIFVEELALKVKSAVEKERDRLNAVLVFPSMPEVMRLN

KLGSFSMSQLGQSKSPFFQLFKKKKSSAGFSDQMLKLVRTLPKVLKYLPSDKAQDARLYI

LSLQFWLGGSPDNLVNFLKMVSGSYVPALKGVKMDYSDPVLYLDSGIWHPLAPCMYDDVK

EYLNWYATRRDANEKLKSSNAPVIGLVLQRSHIVTGDESHYVAVIMELEARGAKVIPIFA

GGLDFSGPVERYFIDPITKKPFVNSVVSLTGFALVGGPARQDHPRAIEALMKLDVPYIVA

LPLVFQTTEEWLNSTLGLHPIQVALQVALPELDGGMEPIVFSGRDPRTGKSHALHKRVEQ

LCTRAIKWGDLKRKSKAEKKLAITVFSFPPDKGNVGTAAYLNVFASIYSVLKDLKKDGYN

VEGLPETSAELIEEVIHDKEAQFSSPNLNVAYKMNVREYQKLTPYATALEENWGKAPGNL

NSDGENLLVYGKQYGNVFIGVQPTFGYEGDPMRLLFSKSASPHHGFAAYYSFVEKIFKAD

AVLHFGTHGSLEFMPGKQVGMSDACFPDSLIGNIPNIYYYAANNPSEATIAKRRSYANTI

SYLTPPAENAGLYKGLKQLSELIASYQSLKDSGRGPQIVSSIISTARQCNLDKDVDLPDE

GQEIDAKERDLVVGKVYSKIMEIESRLLPCGLHIIGEPPTAMEAVATLVNIAALDRAEDD

ISSLPSILAATVGRNIEEIYRGNDNGVLRDVELLRQITEASRGATSAFVERSTNSKGQVV

DNSDKLTSLLGFGINEPWIQYLSNTQFYRADREKLRVLFQFLGECLKLIVANNEVGSLKQ

ALEGKYVEPGPGGDPIRNPKVLPTGKNIHALDPQSIPTTAALQSAKIVVERLLERQKVDN

GGKYPETVALVLWGTDNIKTYGESLAQVMWMIGVRPVADTLGRVNRVEPVSLEELGRPRV

DVVVNCSGVFRDLFINQMNLLDRGIKMVAELDEPEDQNFVRKHALEQAKTLGIDVREAAT

RVFSNASGSYSSNINLAVENSSWNDEKQLQDMYLSRKSFAFDCDAPGVGMMEKRKVFEMA

LSTADATFQNLDSSEISLTDVSHYFDSDPTNLVQNLRKDGKKPSAYIADTTTANAQVRTL

SETVRLDARTKLLNPKWYEGMLSTGYEGVREIEKRLTNTVGWSATSGQVDNWVYEEANTT

FIKDEEMLNRLMNTNPNSFRKLLQTFLEANGRGYWDTSEENIEKLKQLYSEVEDKIEGID

R

>N0DQQ0

MASLVSTPFTLPANKVDQLSSFSQKHYFLHSFLPKKSSNASTSRTQSLNVKCVVAGNGLF

TQTTQEVRRIVPENKQGLPVVKIVYVVLEAQYQSSLSAAVRTLNKSNKFASYEVVGYLVE

ELRDENNYKSFCLDLEDANIFIGSLIFVEELALKVKAAVEKQRDRMDAVLVFPSMPEVMR

LNKLGSFSMSQLGQSKSPFFQLFKNKKKSSAGFSDQMLKLVRTLPKVLKYLPSDKAQDAR

LYILSLQFWLGGSPDNLVNFVKMISGSYIPALKGMDIAYSDPVVFLDNGIWHPLAPCMYD

DVKEYLNWYDTRRDTNEKLKKRDAPVVGLILQRSHIVTGDESHYVAVIMELEAKGAKVIP

IFAGGLDFSGPIEKYLVDPITKKPFVNSVVSLTGFALVGGPAKQDHPRAIEALMKLDVPY

LCALPLVFQTTEEWLNSTLGLHPIQVALQVALPELDGGMELLFRWTDPRTGKSHALHKRV

EQLCTRAIRWADLKRKTKSEKRVAITVFSFPPDKGNVGTAAYLNVFASIFSVLQDLKRDG

YNVEGLPENSAELIEDVLHDKEAQFSSPNLNVVYKMGVREYQQLTPYSTALEENWGKPPG

NLNSDGENLLVYGKQYGNVFIGVQPTFGYEGDPMRLLFSKSASPHHGFAAYYSYVEKIFK

ADAVLHFGTHRSLEFMPGKQVGMSDACFPDSLIGNIPNVYYYAANNPSEATIAKRRSYAN

TISYLTPPAENAGLYKGLKQLSELIASYQSLKDTGRGQQIVSSIISTAKQCNLDKDVDLP

EEGVEISSKERDLVVGKVYSKIMEIESRLLPCGLHVIGEPPSAMEAVATLVNIAALDRPE

EGILSLPSILAETVGREIEDIYRSSDKGILKDVELLKQITDASRGAVSAFVQRSTNSKGQ

VVDMSGKLSSILGFGLNEPWIQYFSDTKFYRADREKLRVLFQFLGDCLKLIVADNELGSL

KQALEGKYVEPGPGGDPIRNPKVLPTGKNIHALDPQSIPTTAAMQSAMVVVDRLLERQKA

DNGGKFPETVALVLWGTDNIKTYGESLGQVLWMIGCRPVADSLGRVNRVEPVSLEELGRP

RIDVVVNCSGVFRDLFINQMNLLDRAVKMVAELDEPLEQNYVRKHALEQAETLGVDVREA

ATRIFSNASGSYSSNVNLAVENSSWNDEKQLQDMYLSRKSFAFDSDAPGTGMAEKRKVFE

MALSTAEATFQNLDSSEISLTDVSHYFDSDPTNLVGSLRKDGKKPNAYIADTTTANAQVR

TLSETVRLDARTKLLNPKWYEGMLSSGYEGVREIEKRLTNTVGWSATSGQVDNWVYEEAN

TTFIKNEEMLNRLMNTNPNSFRKLLQTFLEANGRGYWETSDDNIEKLRQLYSEVEDKIEG

IDR

>O22435

MASLVSSPFTLPNSKVEHLSSISQKHYFLHSFLPKKINPTYSKSPKKFQCNAIGNGLFTQ

TTQEVRRIVPENTQGLATVKIVYVVLEAQYQSSLTAAVQTLNKNGQFASFEVVGYLVEEL

RDENTYKMFCKDLEDANVFIGSLIFVEELALKVKSAVEKERDRLDAVLVFPSMPEVMRLN

KLGSFSMSQLGQSKSPFFELFKKKKPSSAGFSDQMLKLVRTLPKVLKYLPSDKAQDARLY

ILSLQFWLGGSPDNLVNFLKMISGSYVPALKGMKIDYSDPVLYLDNGIWHPLAPCMYDDV

KEYLNWYATRRDTNEKLKSSNAPVVGLVLQRSHIVTCDESHYVAVIMELEAKGAKVIPIF

AGGLDFSRPIERYFIDPITKKPFVNSVISLSGFALVGGPARQDHPRAIEALMKLDVPYIV

ALPLVFQTTEEWLNSTLGLHPIQVALQVALPELDGGMEPIVFAGRDPRTGKSHALHKRVE

QLCTRAIKWGELKRKTKAEKRLAITVFSFPPDKGNVGTAAYLNVFASIYSVLKDLKKDGY

NVEGLPETSAQLIEEVIHDKEAQFSSPNLNIAYKMNVREYQKLTPYATALEENWGKAPGN

LNSDGENLLVYGKQYGNVFIGVQPTFGYEGDPMRLLFSKSASPHHGFAAYYSFVEKIFKA

DAVLHFGTHGSLEFMPGKQVGMSDASFPDSLIGNIPNVYYYAANNPSEATIAKRRSYANT

ISYLTPPAENAGLYKGLKQLSELISSYQSLKDSGRGQQIVNSIISTARQCNLDKDVDLPE

EGEEISAKERDLVVGKVYSKIMEIESRLLPCGLHIIGEPPTAMEAVATLVNIATLDRPEE

GISALPSILAATVGRSIEEIYRGNDQGILRDVELLRQITEASRGAISAFVERTTNNKGQV

VNVNDKLTSILGFGINEPWIQYLSNTQFYRADRDKLRVLFQFLGECLKLIVANNEVGSLK

QALEGKYVEPGPGGDPIRNPKVLPTGKNIHALDPQAIPTIAAVQSAKIVVERLLERQKAD

NGGKYPETVALVLWGTDNIKTYGESLAQVMWMIGVRPVTDSLGRVNRVEPVSLEELGRPR

VDVVVNCSGVFRDLFINQMNLLDRAVKMVAELDEPEDQNYVRKHALEQAKTLGVDVREAA

TRIFSNASGSYSSNINLAVENSTWNDEKQLQDMYLSRKSFAFDCDAPGVGMTEKRKVFEM

ALSTADATFQNLDSSEISFTDVSHYFDSDPTNLVQNLRKDGKKPSAYIADTTTANAQVRT

LSETVRLDARTKLLNPKWYEGMLSTGYEGVREIEKRLTNTVGWSATSGQVDNWVDEEANT

TFIQDQEMLNRLMNTNPNSFRKLLQTFLEANGRGYWETSAENIEKLKQLYSEVEDKIEGI

DR

>O65808

MASLVSSPFTLPSSKPDQLHSLAQKHLYLHSFLPKKANYNGSSKSSLRVKCAVIGNGLFT

QTTQEVRRIVPENDQNLPTVKIVYVVLEAQYQSSITAAVIALNSKRKHASFEVVGYLVEE

LRDAATYKTFCKDLEDANIFIGSLIFVEELALKIKAAVEKERDRLDAVLVFPSMPEVMRL

NKLGSFSMSQLGQSKSPFFQLFKRKKPQSAGFADSMLKLVRTLPKVLKYLPSDKAQDARL

YILSLQFWLGGSPDNLQNFLKMISGSYIPALKGTKIEYSEPVLYLDVGIWHPLAPCMYDD

VKEYLNWYGTRRDANEKLKSPNAPVIGLVLQRSHIVTGDDGHYVAVIMELEARGAKVIPI

FAGGLDFSGPVEKFFIDPITKKPFVNSVVSLTGFALVGGPARQDHPRAVEALMKLDVPYI

VALPLVFQTTEEWLNSTLGLHPIQVALQVALPELDGGMEPIVFAGRDPKTGKSHALHKRV

EQLCIRAIRWAELKRKSKEEKKLAITVFSFPPDKGNVGTAAYLNVFASIYSVMKELKKDG

YNVDGLPETSEALIEDVLHDKEAQFSSPNLNIAYKMNVREYQNLTPYATALEENWGKPPG

NLNADGENLLVYGKQYGNVFIGVQPTFGYEGDPMRLLFSKSASPHHGFAAYYSFVEKIFK

ADAVLHFGTHGSLEFMPGKQVGMSDVCYPDSLIGNIPNVYYYAANNPSEATIAKRRSYAN

TISYLTPPAENAGLYKGLKQLSELISSYQSLKDTGRGAQIVSSIISTAKQCNLDKDVTLP

DEGEEIPPKERDLVVGQVYSKIMEIESRLLPCGLHIIGEPPSALEAVATLVNIAALDRPE

DGISSLPSILADTVGRDIEDVYRGSNKGILKDVELLRQITEASRGAITAFVERTTNNKGQ

VVDVADKLSSILGFGINEPWIQYLSNTKFYRADREKLRTLFVFLGECLKLIVADNEVGSL

KQALEGKYVEPGPGGDPIRNPKVLPTGKNIHALDPQSIPTTAAMQSAKIVVDRLIERQKA

ENGGKYPETIALVLWGTDNIKTYGESLAQVLWMIGVEPVADTFGRVNRVEPVSLEELGRP

RIDVVVNCSGVFRDLFINQMNLLDRAVKMVAELDEPAEQNYVKKHASEQAQALGVEVREA

ATRIFSNASGSYSSNINLAVENSSWNDEKQLQDMYLSRKSFAFDSDAPGAGMTEKRKVFE

MALSTADATFQNLDSSEISLTDVSHYFDSDPTNLVQNLRKDGKKPSAYIADTTTANAQVR

TLSETVRLDARTKLLNPKWYEGMLSTGYEGVREIEKRLTNTVGWSATSGQVDNWVYEEAN

TTFIQDEQMLNKLMSTNPNSFRKLVQTFLEANGRGYWETSEDNIEKLRQLYSEVEDKIEG

IDR

>Q07893

MASLVSSPFTLPNSKVENLSSISQKHYFLHSFLPKKLNQNNKSQKFKCVAIGNGLFTQTT

QEVRRIVPENLKGLPTVKIVYVVLEAQYQSSLTAAVQSLNQNGKYASFEVVGYLVEELRD

PNTYKSLCKDLEDANIFIGSLIFVEELALKVKDAVEKERERLDAVLVFPSMPEVMRLNKL

GSFSMSQLGQSKSPFFQLFKKNKSSAGFADSMLKLVRTLPKVLKYLPSDKAQDARLYILS

LQFWLGGSPDNLVNFLKMISGSYIPALKGTKIEYSDPVLYLDTGIWHPLAPCMYDDVKEY

LNWYGTRRDANEKLKSSKAPIVGLVLQRSHIVTGDESHYVAVIMELEARGAKVIPIFAGG

LDFSGPVEKYFIDPITKKPMVNSVISLTGFALVGGPARQDHPRAIEALMKLDVPYIVAVP

LVFQTTEEWLNSTLGLHPVQVALQVALPELDGGMEPIIFAGRDPRTGKSHALHKRVEQLC

TRAINWGNLTRKKKTEKRVAITVFSFPPDKGNVGTAAYLNVFASIFSVLKDLKKDGYNVE

GLPETAEALIEEIIHDKEAQFNSPNLNIAYKMNVREYQALTPYSAALEENWGKPPGNLNA

DGENLLVYGKQYGNVFIGVQPTFGYEGDPMRLLFSKSASPHHGFAAYYSFVEKIFKADAV

LHFGTHGSLEFMPGKQVGMSDACFPDSLIGNIPNMYYYAANNPSEATIAKRRSYANTISY

LTPPAENAGLYKGLKQLGELISSYQSLKDSGRGPQIVSSIISTARQCNLDKDVELPEEGA

EISAKERDLVVGKVYSKIMEIESRLLPCGLHIIGEPPTAMEAVATLVNIAALDRPEEGIS

ALTSILAETVGRSIEDVYRGSDKGILKDVELLRQITEASRGAITAFVERTTNDKGQVVDV

SNKLTSILGFGINEPWVQYLSNTKFYRADREKLRVLFQFLGECLKLVVANNEVGSLKQAL

EGKFVEPGPGGDPIRNPKVLPTGKNIHALDPQSIPTTAAMQSAMVVVDRLLERQKADNGG

KFPETVALVLWGTDNIKTYGESLAQVLWMIGVKPVSDTFGRVNRVEPVSLEELGRPRVDV

VVNCSGVFRDLFINQMNLLDRAVKMVAELDEPVEQNFVRKHALEQAKELGVEVREAASRI

FSNASGSYSSNINLAVENSSWNDEKQLQDMYLSRKSFAFDSDAPGVGMTEKRKIFEMALS

TADATFQNLDSSEISLTDVSHYFDSDPTNLVQNLRKDGKKPSAYIADTTTANSQVRTLSE

TVRLDARTKLLNPKWYEGMLSSGYEGVREIEKRLTNTVGWSATSGQVDNWVYEEANTTFI

EDEQMLNRLMNTNPNSFRKLLQTFLEANGRGYWETSAENIERLRQLYSEVEDKIEGIDR

>Q19PI2

MFTYVKPTVRHIKPDSLNGRSRLKVVYIVLEAQYQSALSAAVRAINEKNPNLAIEISGYL

IEELRDPENYENLKKDLAEANVFIASLIFIEDLADKVVEAVTPYRDRLDVAVVFPSMPQV

MRLNKMGSFSMAQLGQSKSAIAQFMKKRKEKSGSSFQDGMLKLLQTLPKVLKYMPIDKAQ

DARNFMLSFQYWLGGSPENLENFLLMLAEKYVFKGQEKLHFAEPVTYPDMGIWHPLAPKM

FEDVNDYLNWYNGREDIPDDTKDPLAPCVGLVLQRTHLVTGDDAHYVAMVQELEAMGARV

VAIFAGGLDFSKPVDTYFWDTPPKGIPAQPLVDIVVSLTGFALVGGPARQDHPKAIDSLK

RLNRPYMVALPLVFQTTEEWEESELGLHPIQVALQIAIPELDGAIEPIIVSGRDGATGKA

IALQDRIEAICQRALKWANLRKKPKLDKKVAITIFSFPPDKGNVGTAAYLDVFGSIYEVM

RALKNNGYDIPEIPESPSALMQQVIHDATAQYQSPELNIAYKMSVEQYERLTLYSERLHE

NWGPPPGHLNTDGENLLVYGKSFGNLFIGVQPTFGYEGDPMRLLFSRSASPHHGFAAYYT

YLEQVWGADAVLHFGTHGSLEFMPGKQMGMSGECYPDNLIGTIPNLYYYAANNPSGATIA

KRRSYAETISYLTPAAENAGLYKGLQELNELIGSYQTLKDSGRGVQIVSTIIEKSRMVNL

DQDAALPEQDPGELTQEERDNVVGQVYRKLMEIESRLLPCGLHVIGKPPSAEEAIATLVN

IASLDREEEGIVSLPRIIANSINRDIEELYRNSNQGILADVDLLQHITNATRAAIAALVQ

EQTDAEGRVSKISQLNFFNMGKKEPWLQALHDLGYTQIDPEPMKPLFEYLEFCLKQVCAD

NELGALLRALEGEYILPGPGGDPIRNPDVLPTGKNMHALDPQSIPTVAAVKSAQIVVDRL

LERQKMENGGQYPETIAVVLWGTDNIKTYSESLAQVMWMVGAKPVPDALGRVNKLELIPL

EKLGRPRIDVVISCSGVFRDLFINQMNLLDKAIKMAAEAGEPLEMNFVRKHALQQAAELG

INLRQAATRVFSNASGSYAANVNLAVENSTWESEAELQEMYLNRKSFAFNSDNPGMMGDN

RQMFEASLKTADATFQNLDSSEISLTDVSHYFDSDPTKTIASLRKDGKQPASYIADTTTA

SAQVRTLSETVRLDARTKMLNPKWYEGMLSHGYEGVRELSKRLVNTMGWSATAGAVDNWV

YEETNETFIKDEAMQQRLMSLNPHSFRKMVTTLLEVNGRGYWETSEENLDRLRELYQEVE

DRIEGIE

>Q40001

MSSLVSAPFATATGAQKKARGPRPAPLHSFLLTGRRGRRATIRCAVPGNGLFTQTNPDVH

RVVPAERDLPRVKVVYVVLEAQYQSSSRRRDAAQRRPRRAAEFEVVGYLVEELRDADTYA

AFCDDVAAANVFIGSLIFVEDVALKVRDAVAKHRDRMDAVLVFPSMPEVMRLNKLGSFSM

AQLGQSKSPFFQLFKRNKKDSSGFADSMLKLVRTLPKVLKYLPSDKAQDARLYILSLQFW

LGGSPDNLQNFLKMIAVSYVPALKGADIRYNDPVLFLDTGIWHPLAPTMYDDVKEYLNWY

GTRRDANDRLKNPEAPVIGLVLQRSHIVTGDDGHYVAVIMELEARGAKVIPIFAGGLDFS

GPIERYLVDPITKKPFVNAVVSLTGFALVGGPARQDHPKAIASLMKLDVPYIVALPLVFQ

TTEEWLNSTLGLHPIQVALQVALPELDGGMEPIVFAGRDPRSGKPLIRKSHALHKRVEQL

CTRAIRWAELKRKTKMDKKLAITVFSFPPDKGNVGTAAYLNVFSSIYSVLRDLKKDGYNV

EGLPETPEELIEEVIHDKEAQFNSPNLNVVYRMNVREYQALTPYANMLEENWGKPPGHLN

SDGENLLVYGKQYGNIFIGVQPTFGYEGDPMRLLFSKSASPHHGFAAYYTFVEKIFKADA

VLHFGTHGSLEFMPGKQVGMSDACFPDSLIGNIPNIYYYAANNPSEATVAKRRSYANTIS

YLTPPAENAGLYKGLKQLSELIASYQSLKDTGRGNQIVSSIISTAKQCNLDKDVALPDEG

EELPANERDLVVGKVYGKLMEIESRLLPCGLHVIGEPPTAVEAVATLVNIAALDRPEENI

FSLPGILAATVGRTIEDVYRGSDKGILADVELLKQITEASRGAVGAFVEKSTNSKGQVVD

VTSKLSSILGFGLSEPWVEYLSQTKFIRADRDKLRTLFGFLGECLKLIVADNELGALKTA

LEGSYVEPGPGGDPIRNPKVLPTGKNIHALDPQSIPTAAAMKSAKIVVERLLERQKADNG

GKYPETIALVLWGTDNIKTYGESLAQVMWMLGVEPVTDGLGRVNRVEPVSIEELGRPRID

VVVNCSGVFRDLFINQMNLLDRAVKMVAELDEPIEMNYVRKHAMEQAEELGVSVREAATR

IFSNASGSYSSNVNLAVENASWTDEKQLQDMYLSRKSFAFDSDAPGVGMLEKRKTFELAL

ATADATFQNLDSSEISLTDVSHYFDSDPTKLVQGLRKDGRAPSSSIADTTTANAQVRTLS

ETVRLDARTKLLNPRWYEGMMKSGYEGVREIEKRLTNTVGWSATSGQVDNWVYEEANTTF

IEDEEMRKRLMDTNPNSFRKLLQTFLEANGRGYWETSEDNLERLRELYSEVEDKIEGIDR

>Q93WE2

MQTSSLLGRRTAHPAAGATPKPVAPSPRVASTRQVACNVATGPRPPMTTFTGGNKGPAKQ

QVSLDLRDEGAGMFTSTSPEMRRVVPDDVKGRVKVKVVYVVLEAQYQSAISAAVKNINAK

NSKVCFEVVGYLLEELRDQKNLDMLKEDVASANIFIGSLIFIEELAEKIVEAVSPLREKL

DACLIFPSMPAVMKLNKLGTFSMAQLGQSKSVFSEFIKSARKNNDNFEEGLLKLVRTLPK

VLKYLPSDKAQDAKNFVNSLQYWLGGNSDNLENLLLNTVSNYVPALKGVDFSVAEPTAYP

DVGIWHPLASGMYEDLKEYLNWYDTRKDMVFAKDAPVIGLVLQRSHLVTGDEGHYSGVVA

ELESRGAKVIPVFAGGLDFSDPVNKFFYDPLGSGRTFVDTVVSLTGFALVGGPARQDAPK

AIEALKNLNVPYLVSLPLVFQTTEEWLDSELGVHPVQVALQVALPELDGAMEPIVFAGRD

SNTGKSHSLPDRIASLCARAVNWANLRKKRNAEKKLAVTVFSFPPDKGNVGTAAYLNVFG

SIYRVLKNLQREGYDVGALPPSEEDLIQSVLTQKEAKFNSTDLHIAYKMKVDEYQKLCPY

AEALEENWGKPPGTLNTNGQELLVYGRQYGNVFIGVQPTFGYEGDPMRLLFSKSASPHHG

FAAYYTFLEKIFKADAVLHFGTHGSLEFMPGKQVGMSGVCYPDSLIGTIPNLYYYAANNP

SEATIAKRRSYANTISYLTPPAENAGLYKGLKELKELISSYQGMRESGRAEQICATIIET

AKLCNLDRDVTLPDADAKDLTMDMRDSVVGQVYRKLMEIESRLLPCGLHVVGCPPTAEEA

VATLVNIAELDRPDNNPPIKGMPGILARAIGRDIESIYSGNNKGVLADVDQLQRITEASR

TCVREFVKDRTGLNGRIGTNWITNLLKFTGFYVDPWVRGLQNGEFASANREELITLFNYL

EFCLTQVVKDNELGALVEALNGQYVEPGPGGDPIRNPNVLPTGKNIHALDPQSIPTQAAL

KSARLVVDRLLDRERDNNGGKYPETIALVLWGTDNIKTYGESLAQVMMMVGVKPVADALG

RVNKLEVIPLEELGRPRVDVVVNCSGVFRDLFVNQMLLLDRAIKLAAEQDEPDEMNFVRK

HAKQQAAELGLQSLRDAATRVFSNSSGSYSSNVNLAVENSSWSDESQLQEMYLKRKSYAF

NSDRPGAGGEMQRDVFETAMKTVDVSFQNLDSSEISLTDVSHYFDSDPTKLVASLRNDGR

TPNAYIADTTTANAQVRTLGETVRLDARTKLLNPKWYEGMLASGYEGVREIQKRMTNTMG

WSATSGMVDNWVYDEANSTFIEDAAMAERLMNTNPNSFRKLVATFLEANGRGYWDAKPEQ

LERLRQLYMDVEDKIEGVE
